# The combined usage of Matrine and Osthole inhibited endoplasmic reticulum apoptosis induced by PCV2

**DOI:** 10.1186/s12866-020-01986-2

**Published:** 2020-10-12

**Authors:** Yinlan Xu, Panpan Sun, Shuangxiu Wan, Jianhua Guo, Xiaozhong Zheng, Yaogui Sun, Kuohai Fan, Wei Yin, Na Sun, Hongquan Li

**Affiliations:** 1 College of Veterinary Medicine, Shanxi Agricultural University, Taigu, 030801 Shanxi China; 2 Laboratory Animal Center, Shanxi Agricultural University, Taigu, 030801 Shanxi China; 3 Department of Veterinary Pathobiology, Schubot Exotic Bird Health Center, Texas A&M University, College Station, Texas, TX 77843 USA; 4 Medical Research Council (MRC) Centre for Inflammation Research, Queen’s Medical Research Institute, The University of Edinburgh, Edinburgh, EH16 4TJ UK

**Keywords:** Matrine, Osthole, PCV2, Cap, GRP78, Apoptosis

## Abstract

**Background:**

Porcine circovirus type 2 (PCV2) is an important and common DNA virus that infect pig and can cause immunosuppression and induce apoptosis in the infected cells. To escape the host immune system, PCV2 constantly builds up complex mechanisms or mutates genes, and that is why it is difficult to eradicate complex PCV2 infection by relying on vaccines and single compound. At present, there is few literature reports on the effective prevention and treatment of PCV2 infection by a combination of two or more compounds. Previously, we have demonstrated the anti-PCV2 effect of Matrine in vitro, but its mechanism has not been further evaluated. Literatures have proven that Osthole has a variety of pharmacological activities, and we tested the ability of Osthole to inhibit PCV2 replication in cell culture. Therefore, this study explored the synergistic antiviral effect of Matrine combined with Osthole and their synergistic anti-apoptotic mechanism.

**Results:**

Osthole alone had an anti-PCV2 effect, and then its synergistic anti-PCV2 effect of Osthole and Matrine was better than that of Matrine or Osthole alone as demonstrated by qRT-PCR, IFA and Western blotting results. The anti-apoptotic mechanism of these two compounds by inducing the PERK pathway by PCV2 was elucidated through Annexin V-FITC/PI, JC-1 and Western blotting. Matrine and Osthole combination could inhibit the expression of Cap in Cap-transfected PK-15 cells, thus inhibiting Cap-induced PERK apoptosis. Ribavirin was used as a positive control.

**Conclusions:**

The combination of Osthole and Matrine had the synergistic effect of anti-PCV2 infection by directly inhibiting the expression of PCV2 Cap protein. The combination of these two compounds also inhibited PERK apoptosis induced by PCV2 Cap protein, possibly by regulating the level of GRP78. The results formed a base for further studies on the mechanism of anti-PCV2 in vivo using Matrine and Osthole combination and developing new anti-PCV2 compounds with Cap and GRP78 as therapeutic targets.

## Background

Porcine circovirus type 2 (PCV2) is a main pathogen of porcine circovirus-associated disease with high infection and immunosuppressive properties in pig farms [1]. PCV2 is the smallest single-stranded cyclic DNA virus with an unencapsulated membrane in animal, and the capsid protein (Cap) encoded by the ORF2-encoded gene of PCV2 is considered as a major viral structural protein and a primary immunogen involved in the replication of PCV2 [2]. It has been reported that PCV2 could infect mice, piglets and can cause disease [3, 4], as well as can induce apoptosis [5, 6]. It has caused serious economic losses to the global pig industry for about 57 years.

Since 2007, the PCV2 vaccine has been widely used all over the world [7], and now which is still the main strategy in the prevention and control of porcine circovirus diseases [8, 9]. However, due to the short protection period and co-infection of different genotypes, it is not easy to eliminate the PCV2 from pigs by just vaccination [10, 11]. Therefore, the development of effective anti-PCV2 compound is becoming urgent. However, virus often constantly builds up complex mechanisms or mutates genes to escape recognition and clearance by the host immune system [12]. PCV2 can cause immunosuppression and it is difficult to effectively control the PCV2 infection by vaccination and application of one compound. Therefore, it is necessary to study the synergistic anti-PCV2 effect of two or more compound combinations through multiple targets or pathways. Keeping this in view, based on the guidance of traditional Chinese veterinary medicine theory, when two or more compounds with various pharmacological activities are combined reasonably, a component prescription with clear chemical composition and mechanism of action is formed, which is conducive to exerting synergistic pharmacological effects through multi-targets and multi-pathway, and will provide a new concept for the future research and development of Chinese veterinary drugs.

Matrine has anti-inflammatory [13], anti-viral [14, 15], anti-cancer [16], anti-oxidative [17] and anti-apoptotic activities [18]. We found Matrine inhibited the proliferation of PCV2 in PK-15 cells [19], but still its mechanism of action is not evaluated. Osthole has a broad spectrum of clinical applications in the fields, which is mainly due to its anti-viral [20], anti-inflammatory [21], anti-cancer [22, 23] and anti-apoptotic effects [24]. We speculated that Osthole has an anti-PCV2 effect, and tested the ability of Osthole to inhibit PCV2 replication in cell culture.

Therefore, the synergistic antiviral effect and anti-apoptotic mechanism of Matrine combined with Osthole at the targets and pathways were explored in this study. Ribavirin has proved to have a broad antiviral activity and was thus used as a positive control. This study has provided the theoretical basis for the further studies on the mechanism of anti-PCV2 in vivo using Matrine combined with Osthole and developing new antiviral compounds.

## Results

### Cytotoxicity of compounds on PK-15 cells

**Fig. 1:**
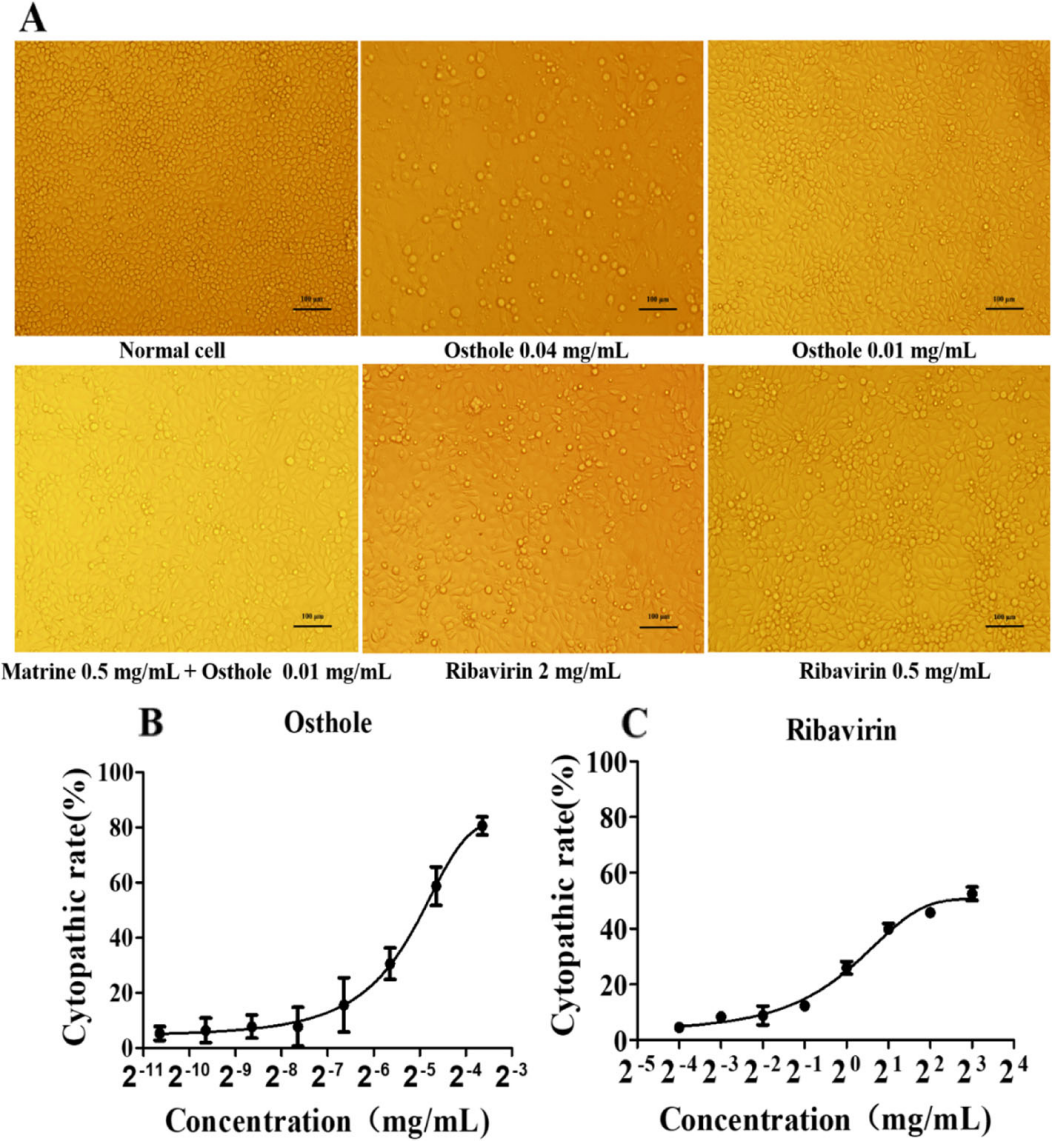
Cytotoxicity of compounds on PK-15 cells detected by CCK8. When the monolayer of PK-15 cells was formed, the compounds were added to the cells and then incubated for 60 h. The morphological changes were observed in the wells treated with Osthole, Matrine combined with Osthole, and Ribavirin. According to the experimental design, the composite image is the product of two time points. **a** when a low concentration (0.01 mg/mL Osthole, 0.5 mg/mL Matrine + 0.01 mg/mL Osthole, and 0.5 mg/mL Ribavirin) was applied, cells were highly refractive and healthy. **b** and **c** CC_50_ curves of Osthole and Ribavirin. The direct correlation of the cytopathic rate with Osthole and Ribavirin is shown. Clearly indicating that the cytotoxicity increased with a higher concentration of Osthole or Ribavirin

The cytopathologic effect (CPE) was monitored under a microscope. Ribavirin was chosen as a positive control. Cells were detached, exhibited round and elongated morphology, when they were treated with 0.04 mg/mL Osthole and 2 mg/mL Ribavirin. (Fig. 1a, for the original images, see Additional file 1). No obvious change in cell morphology was detected when 0.01 mg/mL Osthole, 0.01 mg/mL Osthole + 0.5 mg/mL Matrine, and 0.5 mg/mL Ribavirin were applied. The cytopathic rates at the applied concentrations were 13.68, 16.45 and 12.37%, respectively, which were chosen as the maximum non-toxic concentration (MNTC) used for PK-15 cells. The optical density (OD) value was measured using a microplate reader, and the cytopathic ratio was calculated. The charts for CC_50_ (Fig. 1b and c) and CC_50_ values were generated by GraphPad Prism™ 5.0. CC_50_ was 0.02338 ± 0.003331 mg/mL for Osthole and 0.7119 ± 0.0004273 mg/mL for Ribavirin.

### Reproductive characteristics of PCV2

**Fig. 2:**
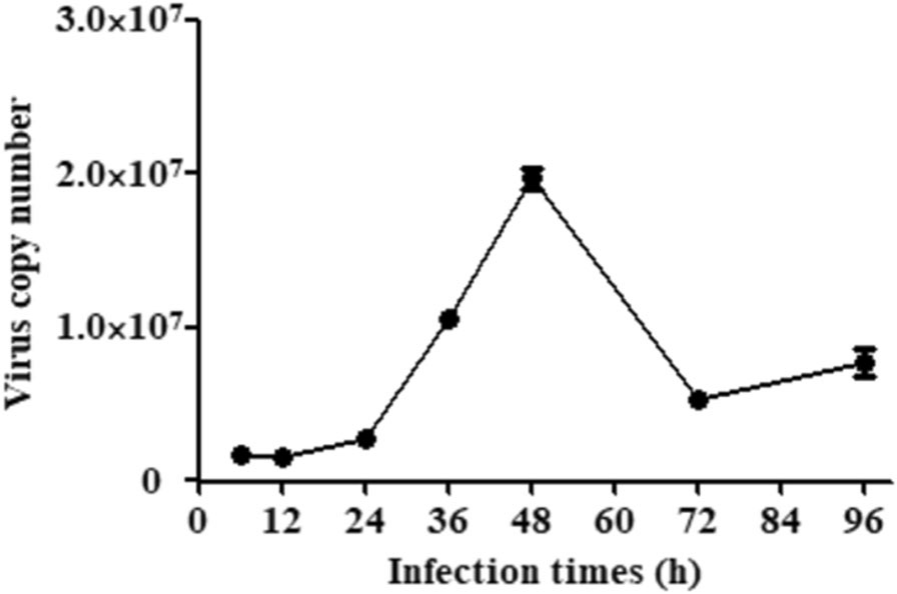
Replication of PCV2 in PK-15 cells. PK-15 cells were infected with 10^4.4^ TCID_50_ of PCV2. Virus copy numbers were detected by qRT-PCR from the samples collected at 6, 12, 24, 48, 72 and 96 h after the initial infection. The chart plotted with virus copy number against the time points after the infection was generated, which showed that the virus replication reached a plateau at 48 h after infection. The virus number was very low in the first 24 h of infection and then dramatically increased between 24 h and 48 h after infection, reaching a plateau at 48 h. Therefore, all samples were collected at 48 h after infection to analyze the anti-viral effect and anti-apoptotic mechanisms of the compounds

To evaluate the viral load at different time points after the cells was infected with 10^4.4^ TCID_50_ of PCV2. The copy of *Cap* gene was determined by quantitative real-time polymerase chain reaction (qRT-PCR). As shown in Fig. 2, the expression of *Cap* gene was increased gradually and reached its peak at 48 h. The expression was then gradually down-regulated. Therefore, the incubation time point of 48 h after PCV2 infection was considered and adopted in the subsequent experiments.

### Synergistic effects of antiviral compounds

**Fig. 3:**
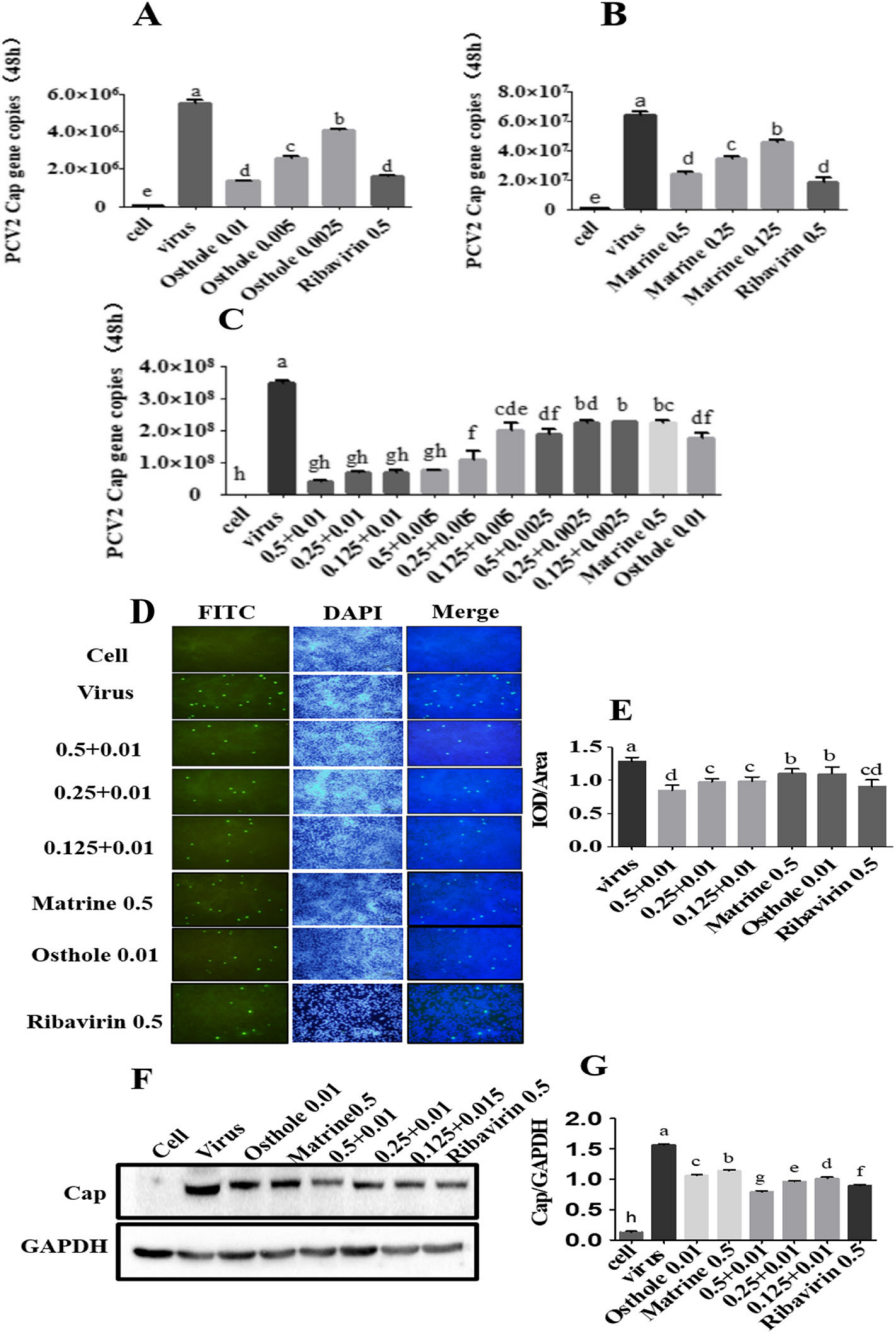
Anti-PCV2 activities of Matrine combined with Osthole detected by qRT-PCR, IFA and Western blotting. **a**-**c** Expression of the *Cap* gene was detected by qRT-PCR in cells treated with compounds; **d** and **e** Expression of Cap protein was detected by IFA; **e** and **g** Expression of Cap was detected by Western blotting and ImageJ was used for the quantification of cap protein bands in Western blotting. GraphPad Prism™ 5.0 was used for the statistical analysis. Data are expressed in the form of mean ± SEM, with different letters (a, b, c, d, etc.) indicating a significant difference from other groups (*p* < 0.05)

To explore the anti-PCV2 effect of compounds, the expression levels of *Cap* gene was detected by qRT-PCR. The concentrations were designed as listed in Table 2. The expression of the *cap* gene in the Osthole group was significantly lower than that of the PCV2 infection group (*p* < 0.05) (Fig. 3a), and the anti-PCV2 effects of Matrine were also verified as shown in (Fig. 3b). The synergistic antiviral effects of Matrine and Osthole were investigated by qRT-PCR (Fig. 3c), the results showed that, compared to the PCV2 infection group, the expression of the *cap* gene in the combined treatment group were significantly decreased (*p* < 0.05). When 0.01 mg/mL Osthole was combined with Matrine at 0.5, 0.25 and 0.125 mg/mL, respectively, the expression level of *Cap* gene was significantly lower than the groups treated with 0.5 mg/mL Matrine and 0.01 mg/mL Osthole alone (*p* < 0.05) (Fig. 3c). However, when 0.5 mg/mL Matrine was combined with 0.01, 0.005 and 0.0025 mg/mL Osthole, respectively, the expression level of *Cap* gene in groups treated with 0.5 + 0.01 and 0.5 + 0.005 was significantly lower than that in groups treated with 0.5 mg/mL Matrine and 0.01 mg/mL Osthole alone (*p* > 0.05), and the expression level of *Cap* gene in groups treated with 0.5 + 0.0025 was significantly lower than that in groups treated with 0.5 mg/mL Matrine (*p* > 0.05), but no significant difference was found in groups treated with 0.01 mg/mL Osthole (*p* < 0.05) (Fig. 3c). The results showed that Osthole may plays a crucial role in the anti-PCV2 effect of combined use. Therefore, 0.01 mg/mL Osthole combined with 0.5, 0.25 and 0.125 mg/mL Matrine were used in the follow up experiments.

The antiviral effects of combined were also verified by inhibition the expression of Cap protein detected by indirect immunofluorescence assay (IFA) and Western blotting. The results showed that the expression level of Cap protein with two compounds treated was significantly lower than the use of Matrine or Osthole alone (*p* < 0.05) (Fig. 3d and f; For the original fluorescence images of Fig. 3d, see Additional file 2; For the original blot images of Fig. 3f, see Additional file 3).

### Inhibition of PCV2-induced apoptosis by compounds combination

**Fig. 4:**
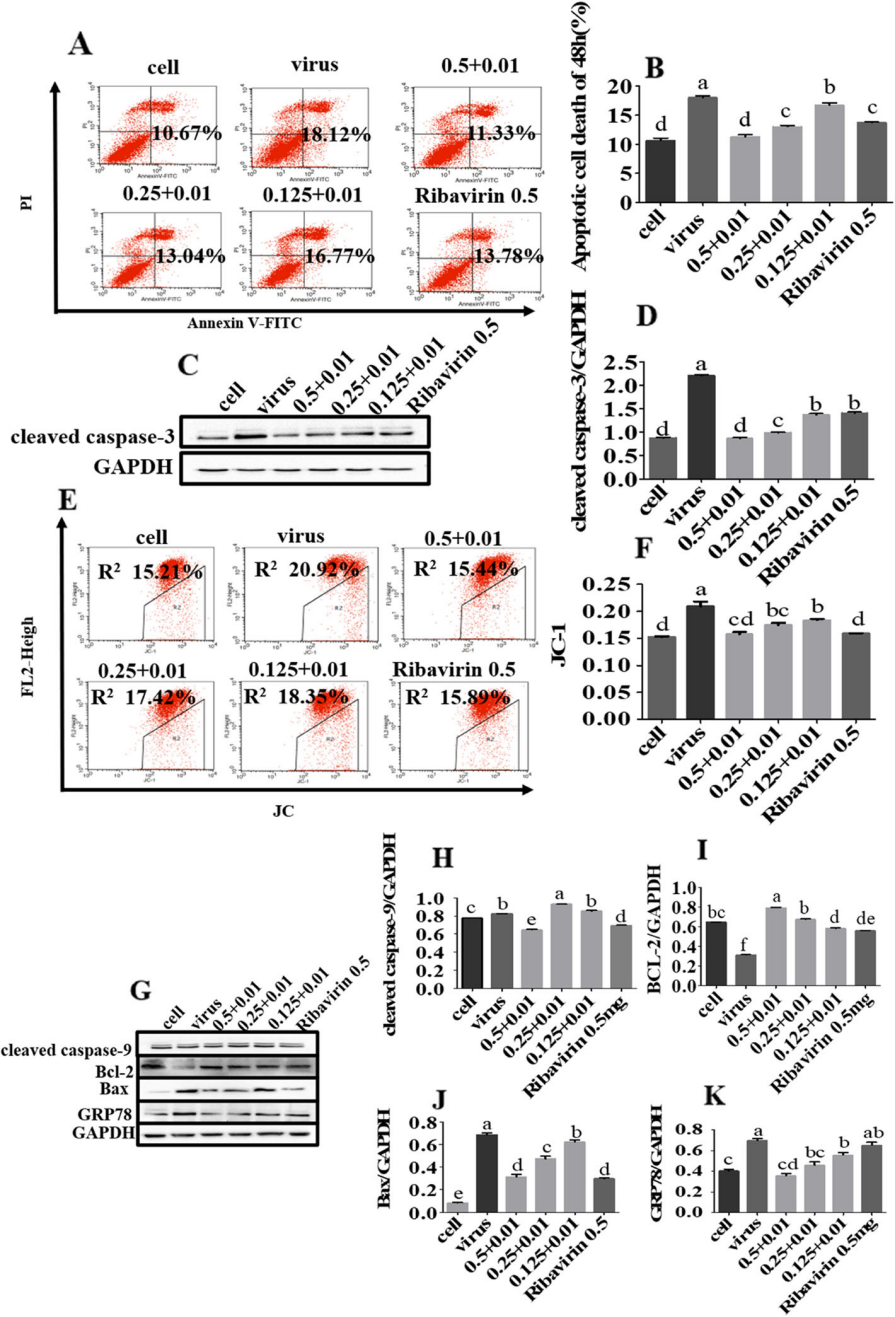
PCV2-induced PK-15 cell apoptosis inhibited by Matrine combined with Osthole. After the PK-15 cells were infected with 10^4.4^ TCID_50_ of PCV2, the cell samples were harvested after 48 h incubation with Matrine combined with Osthole. **a** and **b** The apoptotic rate was analyzed by flow cytometry. Data indicate the sum of the late apoptosis of the right upper quadrant and the early apoptosis of the right lower quadrant. **c** and **d** The level of cleaved caspase-3 was down-regulated significantly in the Western blotting. **e** and **f** Changes in MMP after treatment for 48 h with a combination of drugs were analyzed by JC-1. R2 represents the changes in the MMP. **g**-**k** Expression of the key apoptins were analyzed by Western blotting and grayscale analysis. Data are expressed in the form of mean ± SEM, with different letters (a, ,b, c, d, etc.) indicating a significant difference from other groups (*p* < 0.05)

To investigate the anti-apoptotic effects of compounds combination, the samples were stained with an apoptosis detection kit and analyzed by flow cytometry. The apoptosis rate in the group treated with combined use was significantly lower than that in the PCV2 infection group (*p* < 0.05) (Fig. 4a and b), the expression level of cleaved caspase-3 was significantly down-regulated (Fig. 4c and d), and the mitochondrial membrane potential (MMP) was significantly reduced than in the PCV2 infection group (*p* < 0.05) (Fig. 4e and f). The expression of cleaved caspase-9 expression levels was significantly reduced in the 0.5 + 0.01 combined concentration group (*p* < 0.05), but not in the 0.25 + 0.01 and 0.125 + 0.01 combined concentration groups, compared to the PCV2 control. (*p* > 0.05). However, those of cleaved caspase-3, B-cell lymphoma-2-associated x protein (Bax), and glucose-regulated protein 78 (GRP78) were significantly down-regulated and the expression of B-cell lymphoma-2 (Bcl-2) was significantly elevated (*p* < 0.05) (Fig. 4g-k). As shown in Fig. 4e to k, the down-regulation of MMP was independent on the caspase family but dependent on the Bcl-2 family. It was speculated that the combined use might up-regulate the expression of Bcl-2 in the protein kinase RNA-endoplasmic reticulum kinase (PERK) pathway induced by GRP78 and then down-regulate MMP. For all the original blot images, see Additional file 4.

### Inhibition of PCV2-induced cell apoptosis by combined use via the ER pathway

**Fig. 5:**
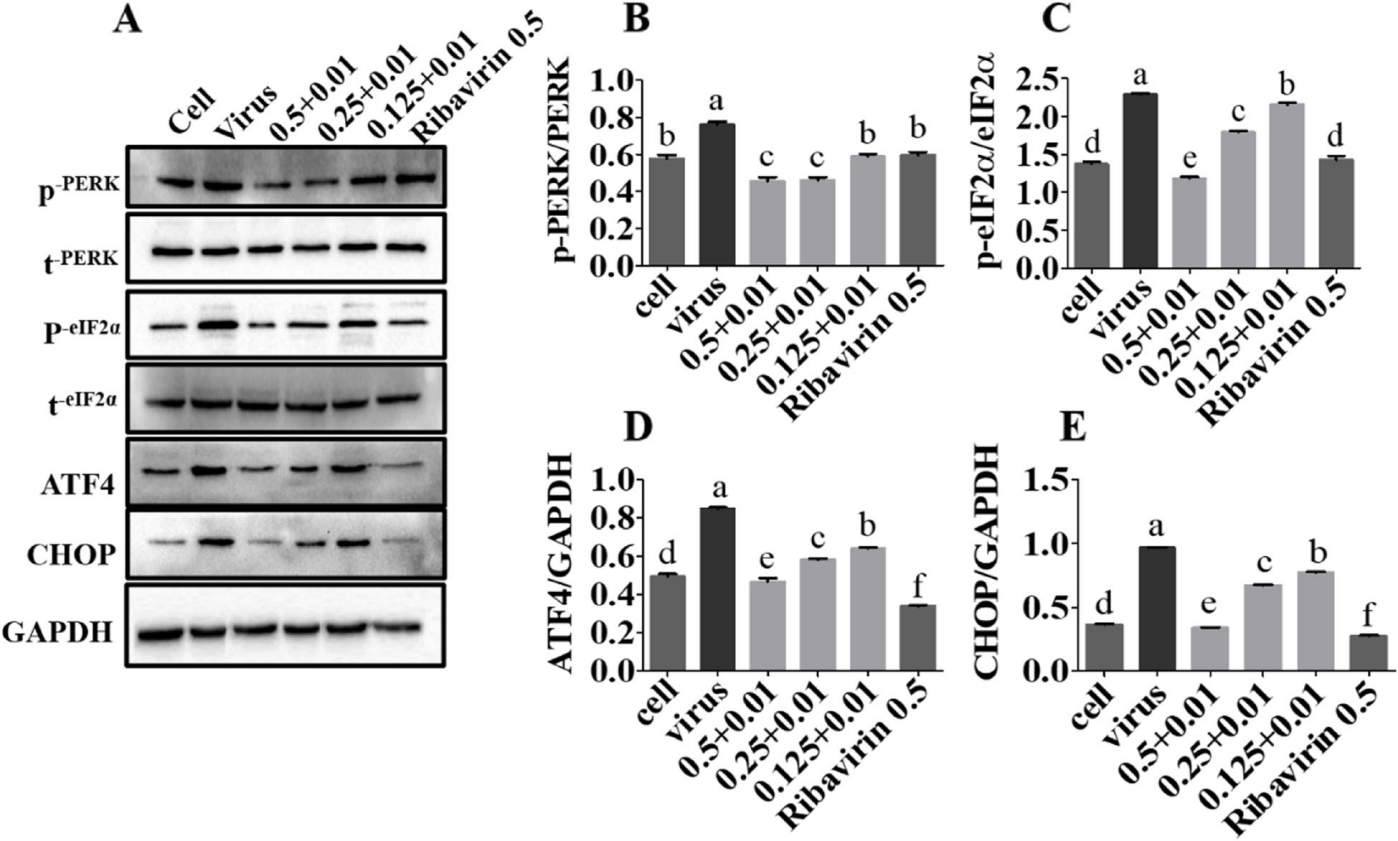
Inhibition of PCV2-induced cell apoptosis by Matrine combined with Osthole via the ER pathway. After the PK-15 cells were infected with10^4.4^ TCID_50_ of PCV2 for 2 h, cells were treated with Matrine and Osthole combination for 48 h, and cell samples were collected. **a**-**e** The expression levels of each apoptin in the PERK pathway were analyzed. After treatment with Matrine and Osthole combination, the levels of p-PERK, p-eIF2α, ATF4 and CHOP were down-regulated. Data are expressed in the form of mean ± SEM, with different letters (a, b, c, d, etc.) indicating a significant difference from other groups (*p* < 0.05)

The results shown that (Fig. 5a-e), compared to the PCV2 infection group, the expression of phosphor-PERK (p-PERK), phosphor-eukaryotic translation initiation factor 2α (p-eIF2α), activating transcription factor 4 (ATF4), and C/EBP homologous protein (CHOP) in the group treated with Matrine combined with Osthole at different concentrations were significantly decreased (*p* < 0.05), indicating that the combined use could inhibit the PCV2-induced endoplasmic reticulum (ER) apoptosis through the PERK pathway. For all the original blot images, see Additional file 5.

### Intervention of combined use on cell apoptosis through the PERK pathway in cells transfected with cap

**Fig. 6:**
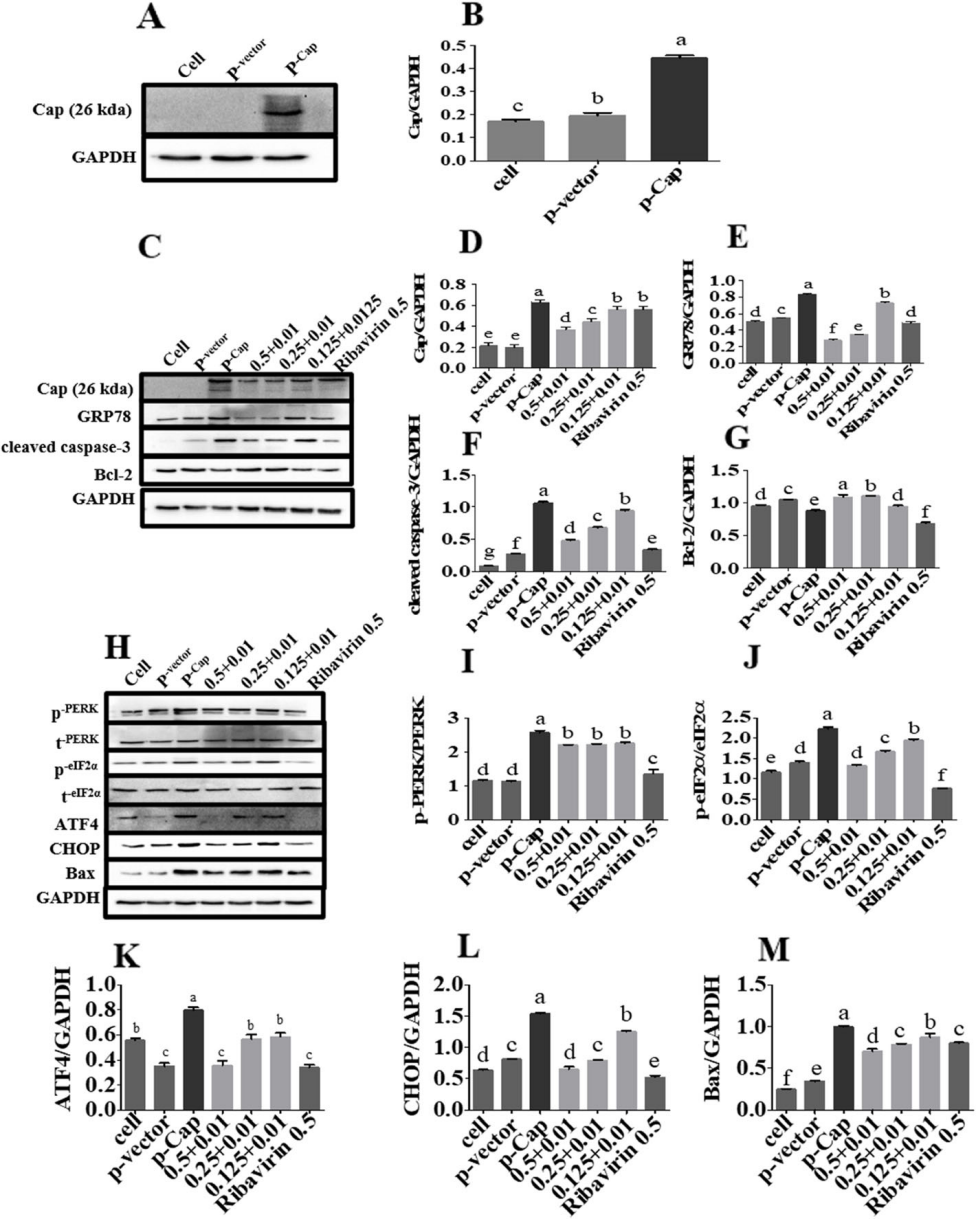
Matrine combined with Osthole inhibits via the PERK pathway apoptosis in the cells transfected with Cap. After the PK-15 cells were transfected with p-Cap for 6 h, the cells were treated with Matrine and Osthole combination for 48 h and the samples were harvested. **a** and **b** The level of Cap protein in the group transfected with p-Cap was markedly elevated in the Western blotting. **c**-**g** As revealed by Western blotting, Matrine combined with Osthole down-regulated the marker protein GRP78 in the ER pathway, as well as the expression levels of the cleaved caspase-3 and Bcl-2 in the PERK apoptosis pathway. **h**-**o** Levels of apoptins in the PERK pathway were determined by Western blotting. Data are expressed in the form of mean ± SEM, with different letters (a, b, c, d, etc.) indicating a significant difference from other groups (*p* < 0.05)

The expression of Cap protein was detected in the plasmids carrying the *Cap* gene transfected to cells by Western blotting, but the expression was not detected in cells transfected with plasmids without carrying the *cap* gene (Fig. 6a and b). The expression level of Cap, GRP78 and cleaved caspase-3 in the combined treated groups were significantly down-regulated than in the Cap transfection group (*p* < 0.05), whereas the expression level of Bcl-2 showed a significant upward trend (*p* < 0.05) (Fig. 6c-g). However, the expression level of the apoptins p-PERK, p-eIF2α, ATF4, CHOP and Bax in the PERK pathway were significantly elevated than in the Cap transfection group (*p* < 0.05) (Fig. 6h-m). In summary, Matrine combined with Osthole could inhibit the Cap-induced apoptosis through the PERK pathway. For all the original blot images involved, see Additional file 6.

## Discussion

We have preliminarily screened and demonstrated the anti-PCV2 effect of Matrine from a variety of compounds, but the anti-PCV2 mechanism of Matrine is still unknown [19]. Osthole has anti-hepatitis B viral effect [20] and which is also a DNA virus. We speculated that Osthole has an anti-PCV2 effect, and confirmed the ability of Osthole to inhibit PCV2 replication in cell culture by qRT-PCR. From the level of protein and gene, we have confirmed that Matrine combined with Osthole has a synergistic and better anti-PCV2 effect than Matrine and Osthole alone. This conclusion provides a theoretical basis for our further studying the mechanism of Matrine combined with Osthole against PCV2 induced apoptosis.

Literature has reported that Matrine has anti-apoptotic effect through the ER pathway and Osthole has an anti-apoptotic effect as well [18, 25, 26]. In this study, the synergistic inhibitory effect of Matrine and Osthole on PCV2-induced apoptosis was demonstrated, and the result was consistent with the conclusion that Matrine combined with Osthole have stronger anti-apoptosis effects. PCV2 has been reported to induce mitochondrial apoptosis in PK-15 cells [27, 28]. MMP is recognized as a marker of mitochondrial statement. Our results showed that Matrine combined with Osthole decreased the level of MMP. MMP can induce apoptosis by the Bcl-2 family, caspase family or by the mitochondrial membrane itself [29]. The proteins in caspase and Bcl-2 families were analyzed by Western blotting. Only the expression of cleaved caspase-9 in the group treated with 0.5 mg/mL Matrine + 0.01 mg/mL Osthole showed a downward trend while the expression of cleaved caspase-3 and Bax were down-regulated and the expression level of Bcl-2 was up-regulated. Thus, it is speculated that combined usage may down-regulate MMP and thus inhibit the occurrence of mitochondrial apoptosis by regulating Bcl-2 and Bax proteins of the Bcl-2 family, but the mechanism of inhibiting mitochondrial apoptosis requires further investigation.

ER is an important site for viral replication and maturation. The up-regulation of the molecular chaperone GRP78 is an ER stress marker [30]. GRP78 can activate IRE1, PERK, and ATF6 pathways [17]. Zhou et al. [31] reported that PCV2 could selectively activate the PERK pathway but not the ATF6 and IRE1a/XBP1 pathways. Cleaved caspase-3, Bax, and Bcl-2 are also downstream proteins of the PERK apoptosis pathway. To further explore the implication of the changes in cleaved caspase-3, Bcl-2, and Bax in the ER apoptosis pathway, GRP78 and apoptotic proteins of the PERK pathway were analyzed by Western blotting. The result showed that the expression of GRP78, p-PERK, p-eIF2α, ATF4, and CHOP showed a downward trend, indicating that Matrine combined with Osthole splayed an anti-PCV2 effect by inhibiting the expression of proteins in the PERK pathway with GRP78 as the target.

Cap is a major viral structural protein and major immunogen involved in viral replication [2]. Matrine combined with Osthole directly inhibited the expression level of PCV2 Cap protein to suppress apoptosis induced by PCV2. To further study the effect of Matrine combined with Osthole on PCV2-induced apoptosis by directly inhibiting the expression of PCV2 Cap or by inhibiting apoptosis of the cells to indirectly play the role of anti-PCV2, the cell model transfected with *Cap* gene was established to study the apoptosis. The results of Western blotting showed that Matrine combined with Osthole directly inhibit the expression level of Cap protein and thus inhibited Cap-induced apoptosis through the PERK pathway.

## Conclusion

**Fig. 7:**
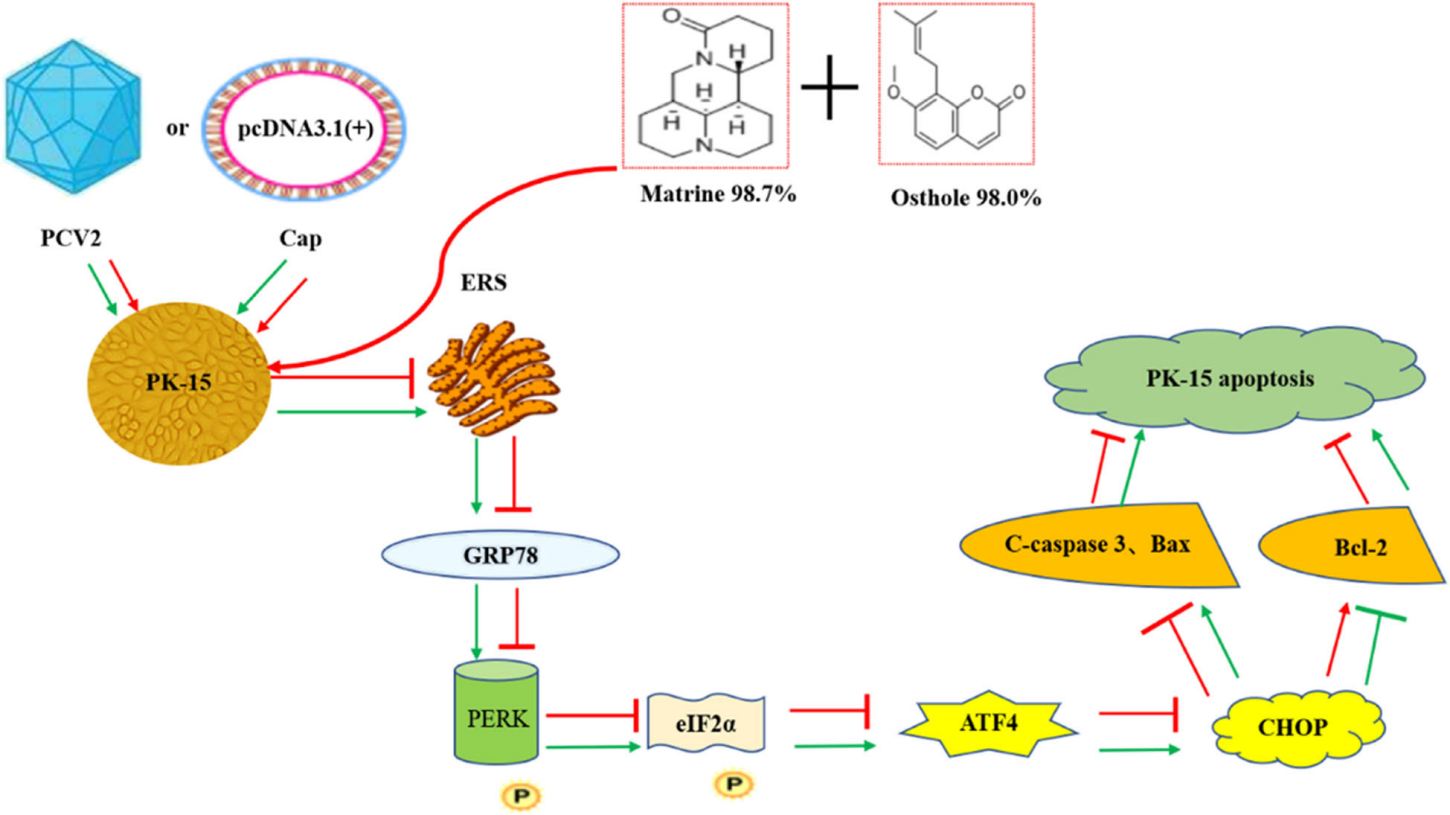
Mechanism of anti-apoptosis induced by PCV2 or Cap using Matrine combined with Osthole through by ER pathway. The ‘arrow’ indicates a promoting effect, whereas red ‘T-shape’ indicates an inhibiting effect. Virus replication is inhibited by inhibiting the expression of apoptins in the PERK pathway of ER, further down-regulating the expression of downstream pro-apoptins cleaved caspase-3 and Bax, and up-regulating the expression of anti-apoptins Bcl-2

In summary, the synergistic anti-PCV2 effect of Matrine and Osthole was demonstrated. And the expression of PCV2 Cap protein was directly inhibited by Matrine combined with Osthole and further inhibited the apoptosis induced by PCV2 infection through the PERK/eIF2α/ATF4/CHOP/Bcl-2 pathway using Cap and GRP78 as the possible anti-PCV2 targets (Fig. 7). The results provide a theoretical basis for further study on the anti-PCV2 mechanism of multi-targets and multi-pathways in vivo combined with Matrine and Osthole, the development of new anti-PCV2 compounds targeting Cap and GRP78.

## Methods

### Cells, viruses, compounds and antibodies

Non-PCV-infected pig kidney epithelial cell lines (PK-15) was cultured, passaged, and frozen in liquid nitrogen.

The PCV2-SH strain was gifted by Professor Jiang Ping of Nanjing Agricultural University. PK-15 cells were used for virus propagation. 10^6.4^ TCID_50_/mL of virus titer was determined by IFA.

**Fig. 8:**
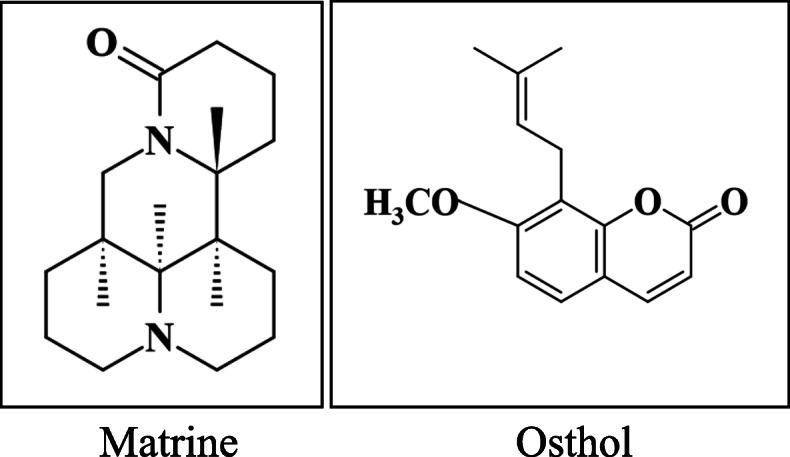
Chemical structure of Matrine and Osthole

Matrine and Osthole were purchased with a defined plant origin, chemical structure, concentration, and specific biological activity. Matrine (98.7% purity) was purchased from the National Institutes for Food and Drug Control (lot no. 110805–201,709). Osthole (98.0% purity) was purchased from Nanjing Zelang Biotechnology Co., Ltd., China (lot no. ZL20171212SCZS). Ribavirin with 99.0% purity was purchased from Beijing Solarbio Technology Co., Ltd., China (CAS: 36791–04-5). The chemical structures of Matrine and Osthole are shown in Fig. 8.

Antibodies against Cap were respectively purchased from Biorbyt LLC. (San Francisco, CA, USA). GRP78, PERK, eIF2α, p-eIF2α, ATF4, CHOP, Bcl-2, Bax and cleaved caspase-3 were purchased from Abcam (Cambridge, MA, USA). Cleaved caspase-9 and p-PERK were purchased from Bioworld Technology Co., Ltd. (Beijing, China) and Immunology Biology Technology Co., Ltd. (Beijing, China), respectively; Glyceraldehyde 3-phosphate dehydrogenase (GAPDH) and horseradish peroxidase-conjugated secondary antibodies were purchased from Wuhan Sanying Biology Technology Co., Ltd. (Wuhan, China) and ComWin Biotech Co., Ltd. (Beijing, China), respectively.

### Cytotoxicity assay

PK-15 cells were seeded onto a 96-well plate at 1 × 10^6^ cells/mL and cultured with Dulbecco’s modified Eagle’s medium (DMEM; Gibco, Waltham, MA, USA) containing 10% fetal calf serum (FCS; 10% DMEM). When cell confluency reached 80–90%, the proper concentration of compounds was added. Osthole was dissolved in 1% dimethyl sulfoxide (Solarbio, China), and then diluted in DMEM containing 2% FCS (2% DMEM), and eight dilutions of the compounds in twofold serial dilutions were prepared. Ribavirin (positive control) was dissolved in 2% DMEM and then prepared in serial dilutions. In this laboratory, Sun et al. [19] confirmed that 0.5 mg/mL of Matrine has an anti-PCV2 replication effect in PK-15 cells. Keeping this in view, the maximum dose of Matrine was set to 0.5 mg/mL in the study. The dilution of Matrine combined with Osthole is shown in Table 1. The equipment used for cytopathic image acquisition was Olympus IX81 (objective lenses: LUCPLANFLN20X/PH1). These micrographs were photographed at a resolution of 4080 × 3072 with the cameras of DP71, and the image acquisition was obtained using CELLsens software, which enhanced the images to 300 dpi. The halogen lamp used for transmitted light Phase- contrast imaging was 12 V, 100 W.

**Table 1:** The final concentration design of Matrine combined with Osthole

Test number	Matrine (mg/mL)	Osthole (mg/mL)
1	0.5	0.01
2	0.25	0.01
3	0.125	0.01
4	0.5	0.005
5	0.25	0.005
6	0.125	0.005
7	0.5	0.0025
8	0.25	0.0025
9	0.125	0.0025
10	0.5	–
11	0.25	–
12	0.125	–
13	–	0.01
14	–	0.005
15	–	0.0025

Cell were cultured for 60 h, 100 μL of the fresh DMEM with 10% Cell Counting Kit-8 (Boster, Wuhan, China) was added and cells were then incubated for another hour at 37 °C. The OD at 450 nm was measured using a microplate reader (Spectra Max M5, Molecular Devices, San Jose, CA, USA). The cytopathic ratio was calculated based on the OD value with the following formula: [(*A* - *B*)/*A* × 100], in which *A* and *B* were the OD value of control and treated cells, respectively. Then, the MNTC and CC_50_ of PK-15 cells were calculated using GraphPad Prism™ 5.0 (GraphPad, Inc., LaJolla, CA, USA).

### qRT-PCR

The PCV2 propagation was determined by qRT-PCR. When cell confluency reached 80–90% in the 24-well plate, cells were infected with 10^4.4^ TCID_50_ of PCV2 for 2 h. The virus was removed from the wells and the wells were washed with PBS for two times, following by addition of 2% DMEM. Cells were incubated for 6, 12, 24, 48, 72 and 96 h. Samples were collected and the DNA was extracted from the PK-15 cell. The copy number of the *Cap* gene was detected by qPCR. Primers 5′- TAC ATT TCC AGC AGT TTG and 5′- CTC CCG CCA TAC CAT AA were used to amplify the PCR products with 148 bp.

The inhibition of PCV2 replication by compounds was also detected by qRT-PCR. The proper concentration of Matrine, Osthole, Matrine combined with Osthole, and Ribavirin was added to the cells after PCV2 infection for 2 h. DNA was extracted and the *Cap* gene was detected after incubation for 48 h. Standard curve of generate by recombinant plasmid vectors containing PCV2 *Cap *gene fragments was used. The concentrations used are shown in Table 2.

**Table 2:** The Final concentration design of different compounds (mg/mL)

	Matrine	Osthole	Matrine + Osthole	Ribavirin
1	0.5	–	–	–
2	0.25	–	–	–
3	0.125	–	–	–
4	–	0.01	–	–
5	–	0.005	–	–
6	–	0.0025	–	–
7	–	–	0.5 + 0.01	–
8	–	–	0.25 + 0.01	–
9	–	–	0.125 + 0.01	–
10	–	–	0.5 + 0.005	–
11	–	–	0.25 + 0.005	–
12	–	–	0.125 + 0.005	–
13	–	–	0.5 + 0.0025	–
14	–	–	0.25 + 0.0025	–
15	–	–	0.125 + 0.0025	–
16	–	–	–	0.5

### IFA

Cells were infected with 10^4.4^ TCID_50_ of PCV2 for 2 h. Then, Matrine (0.5 mg/mL), Osthole (0.01 mg/mL), Matrine combined with Osthole (0.5 + 0.01, 0.25 + 0.01 and 0.125 + 0.01), and Ribavirin (0.5 mg/mL) were added. Cells were cultured for 48 h at 37 °C. A mixture of ice-cold acetone and methyl alcohol (1:1) was used to fix the cells for 30 min at − 20 °C and cells were washed twice with phosphate-buffered saline (PBS). PCV2 IFA kit (China Institute of Veterinary Drug Control, Beijing, China) was used to stain the cells. The specific information on IFA image acquisition and processing was the same as the cytopathic test. Besides, fluorescein isothiocyanate (FITC)-labeled cells were sequentially excited with a WU excitation block and a WB excitation. Mercury lamp was used for reflection fluorescence imaging.

### Annexin V/propidium iodide (PI) staining for apoptosis

Different concentrations of Matrine combined with Osthole (high, medium, and low) were added after infection with 10^4.4^ TCID_50_ of PCV2 for 2 h. Cells were cultured for 48 h and then collected for staining with Annexin V-FITC/PI detection kit (Keygen Biotech, Nanjing, China). The samples were analyzed by flow cytometry (BD Biosciences, Franklin Lakes, NJ, USA).

### Western blotting

The anti-PCV2 activity and antiapoptotic mechanisms of the combined compounds were determined by Western blotting. Cells in six-well plates were infected with 10^4.4^ TCID_50_ PCV2 for 2 h and then treated with the compounds for 48 h. The total protein was extracted and was determined using the BCA protein concentration detection kit (Beyotime Biotechnology, Jiangsu, China). The protein samples were separated by sodium dodecyl sulfate-polyacrylamide gel electrophoresis (SDS-PAGE) and then transferred to a polyvinylidene fluoride (PVDF) membrane. The membrane was blocked for 2 h with 5% skim milk and then incubated with the primary antibody at 4 °C overnight. After washing three times with TBS-Tween 20 (TBST), the membrane was incubated with the secondary antibody at room temperature for 1.5 h and then the bands were detected using the eECL Western Blotting detection kit (Cwbio, Beijing, China) and chemiluminescence imaging system (BIO-RAD, Hercules CA, USA).

### Effects of the drugs combination on MMP detected by JC-1

Cells in six-well plates were incubated with 10^4.4^ TCID_50_ of PCV2 for 2 h and then treated with the compounds for 48 h. The harvested cells were treated with JC-1 MMP detection kit (Beyotime Biotechnology, Jiangsu, China) and analyzed by flow cytometry.

### Cap recombinant plasmid construction, cell transfection, and its mechanism on apoptosis as analyzed by Western blotting

The optimizing *Cap* gene sequence was inserted into plasmid pCDNA3.1 at the *KpnI* and *XhoI* cutting sites. The plasmid was transformed into the *Escherichi coli*. Bacteria with the right Cap sequence were used for the plasmid extraction with GoldHi EndoFree Plasmid Maxi Kit (CW2104M, Cwbio, China). The plasmid concentration was determined using a biological mass spectrometer (D30, Eppendorf, Hamburg, Germany).

When cells confluency in six-well plates reached 70–90%, the pCDNA3.1-Cap (p-Cap) and pCDNA3.1-vector (p-vector) alone were transfected using 7.5 μL Lipofectamine 2000 (Invitrogen, Carlsbad, CA, USA), respectively, and 2.5 μg p-Cap were mixed with 125 μL Opti-MEM. The mixture was incubated for 15 min at room temperature. Then, 50 μL of the mixture were added dropwise to the cells, and cells were incubated for 6 h. Matrine combined with Osthole were added and cultured for 48 h, p-vector and Ribavirin were used as control. The proteins expression levels were analyzed by western blotting for the Cap protein and the key apoptins of the PERK pathway.

### Statistical analysis

CC_50_ was calculated using nonlinear regression. The results of “log (inhibitor) vs. response-variable slope” and the data of generated by qRT-PCR, IFA, Western blotting, Annexin V-FITC/PI, and JC-1 were all analyzed by GraphPad Prism™ 5.0. ImageJ (National Institutes of Health, Bethesda, MD, USA) was used to measure the grey intensity of protein bolts. All data are expressed as the mean ± standard error of the mean (SEM) of at least 3 repeated experiments. The statistical significance threshold was set at *p* < 0.05.

## Supplementary information

**Additional file 1.** Original microscopic images of Fig. 1a. (a) Normal cell group, (b) 0.04 mg/mL Osthole, (c) 0.01 mg/mL Osthole, (d) 0.5 mg/mL Matrine + 0.01 mg/mL Osthole, (e) 2 mg/mL Ribavirin, and (f) 0.5 mg/mL Ribavirin.

**Additional file 2.** Original IFA images of Fig. 3d. (a) Cell group, (b) virus group, (c) 0.5 mg/mL Matrine + 0.01 mg/mL Osthole, (d) 0.25 mg/mL Matrine + 0.01 mg/mL Osthole, (e) 0.125 mg/mL Matrine + 0.01 mg/mL Osthole, (f) 0.5 mg/mL Matrine, (g) 0.01 mg/mL Osthole, and (h) 0.5 mg/mL Ribavirin.

**Additional file 3.** Original blot images of Fig. 3f. (a and b) Original blot images of Cap and GAPDH, respectively.

**Additional file 4.** Original blot images of Fig. 4c and g. (a and b) Original blot images of cleaved caspase-3 and GAPDH in the Fig. 4c, respectively. (c and g) Original blot images of cleaved caspase-3 and GAPDH, respectively. (c-g) Original blot images of cleaved caspase-9, Bcl-2, Bax, GRP78, and GAPDH in the Fig. 4g, respectively.

**Additional file 5.** Original blot images of Fig. 5a. (a-g) Original blot images of p^-PERK^, t^-PERK^, p^-eIF2α^, t^-eIF2α^, ATF4, CHOP and GAPDH, respectively.

**Additional file 6.** Original blot images of Fig. 6a, c and h. (a and b) Original blot images of Cap and GAPDH in the Fig. 6a, respectively. (C and g) Original blot images of Cap, GRP78, cleaved caspase-3, Bcl-2 and GAPDH in the Fig. 6c, respectively. (h-o) Original blot images of p^-PERK^, t^-PERK^, p^-eIF2α^, t^-eIF2α^, ATF4, CHOP, Bax and GAPDH in the Fig. 6c, respectively.

## Data Availability

All data generated or analyzed during this study are included in this published article and the datasets are provided from the corresponding author on reasonable request.

## Abbreviations

ATF4: Activating transcription factor 4
Bax: Bcl-2-associated x protein
Bcl-2: B-cell lymphoma-2
CHOP: C/EBP-homologous protein
eIF2α: Eukaryotic translation initiation factor 2α
GRP78: Glucose-regulated protein 78
MNTC: Maximum non-cytotoxic concentration
PCV2: Porcine circovirus type 2
PERK: Protein kinase RNA-like endoplasmic reticulum kinase
qRT-PCR: Quantitative real-time polymerase chain reaction
