# Supplementary material for: The combined usage of Matrine and Osthole inhibited endoplasmic reticulum apoptosis induced by PCV2
Source: BMC Microbiol. 2020 Oct 12;20:303. doi: 10.1186/s12866-020-01986-2 (PMC7549248; doi:10.1186/s12866-020-01986-2)

**The original cell images of Fig. 2A**

**(a)** Normal cell


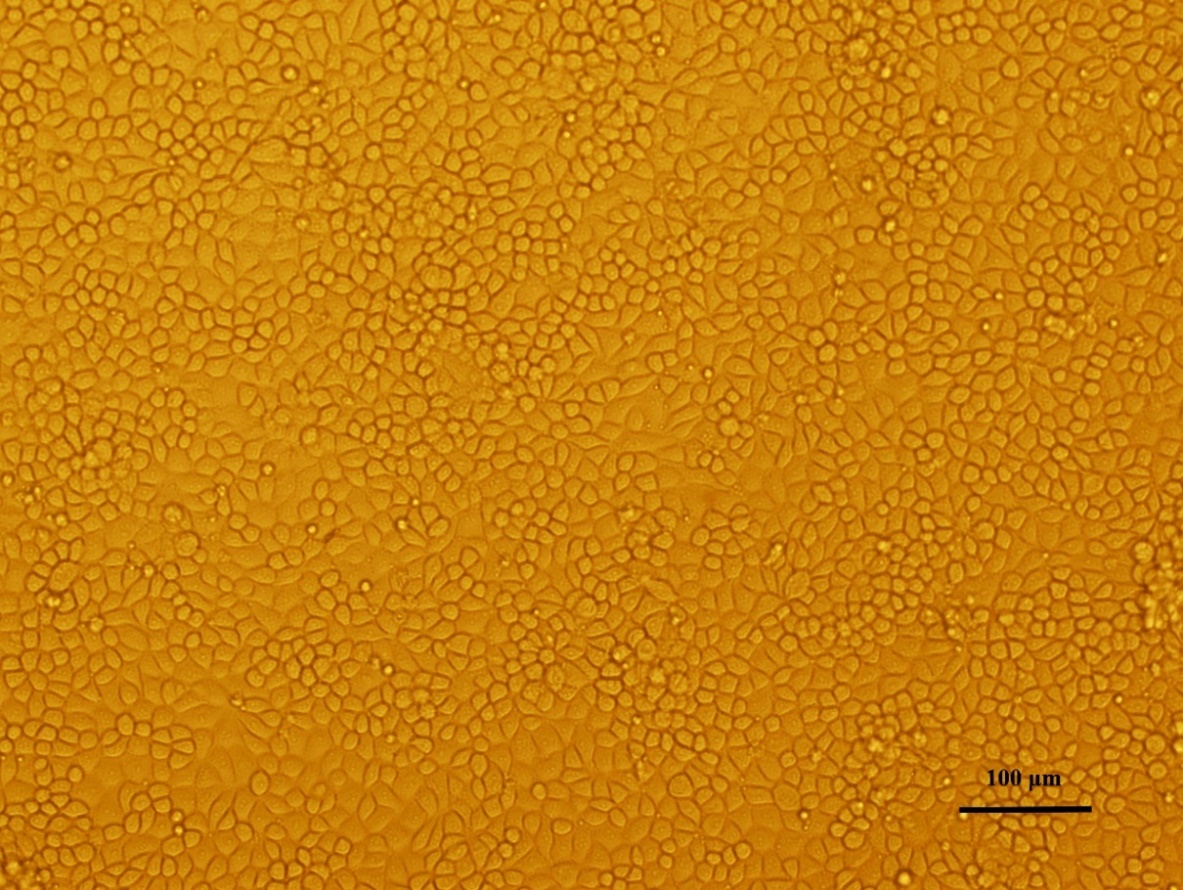


**(b)** Osthole 0.04 mg/mL


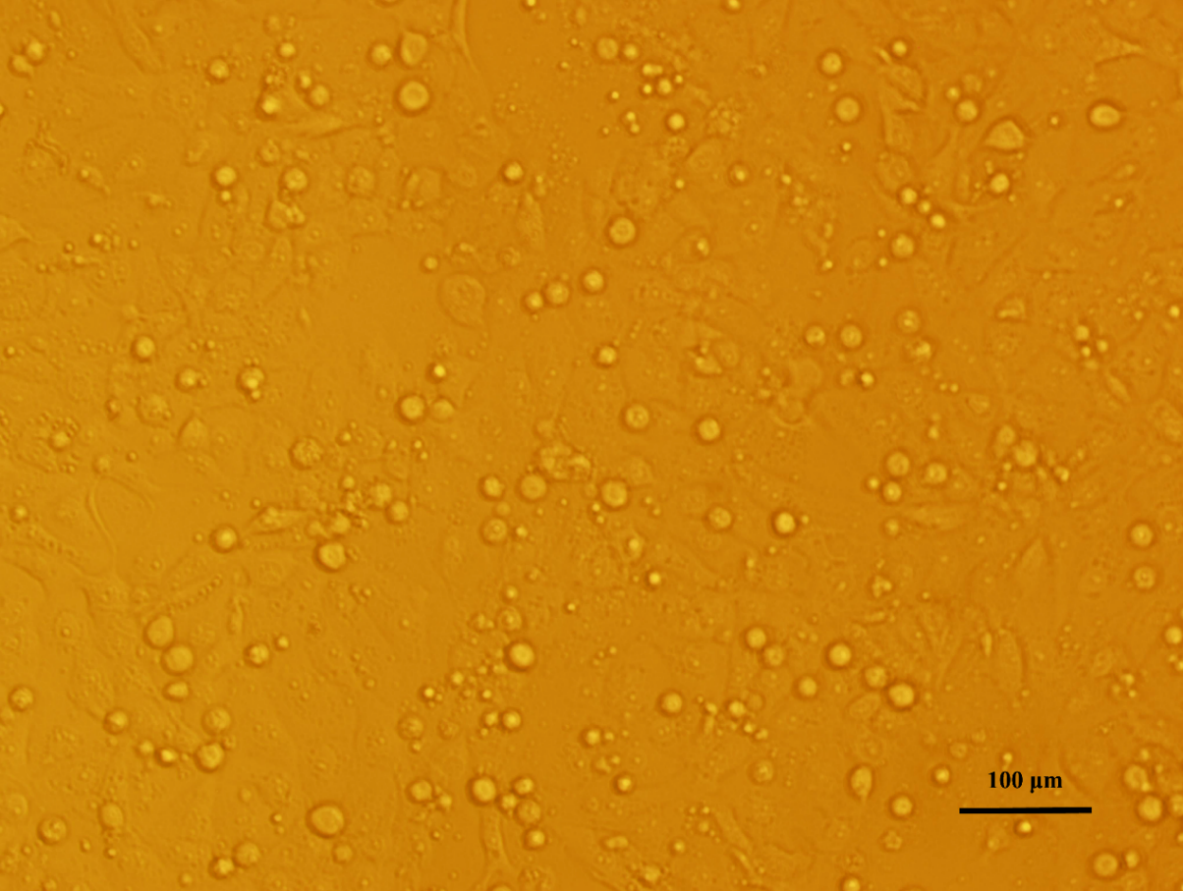


**(c)** Osthole 0.01 mg/mL


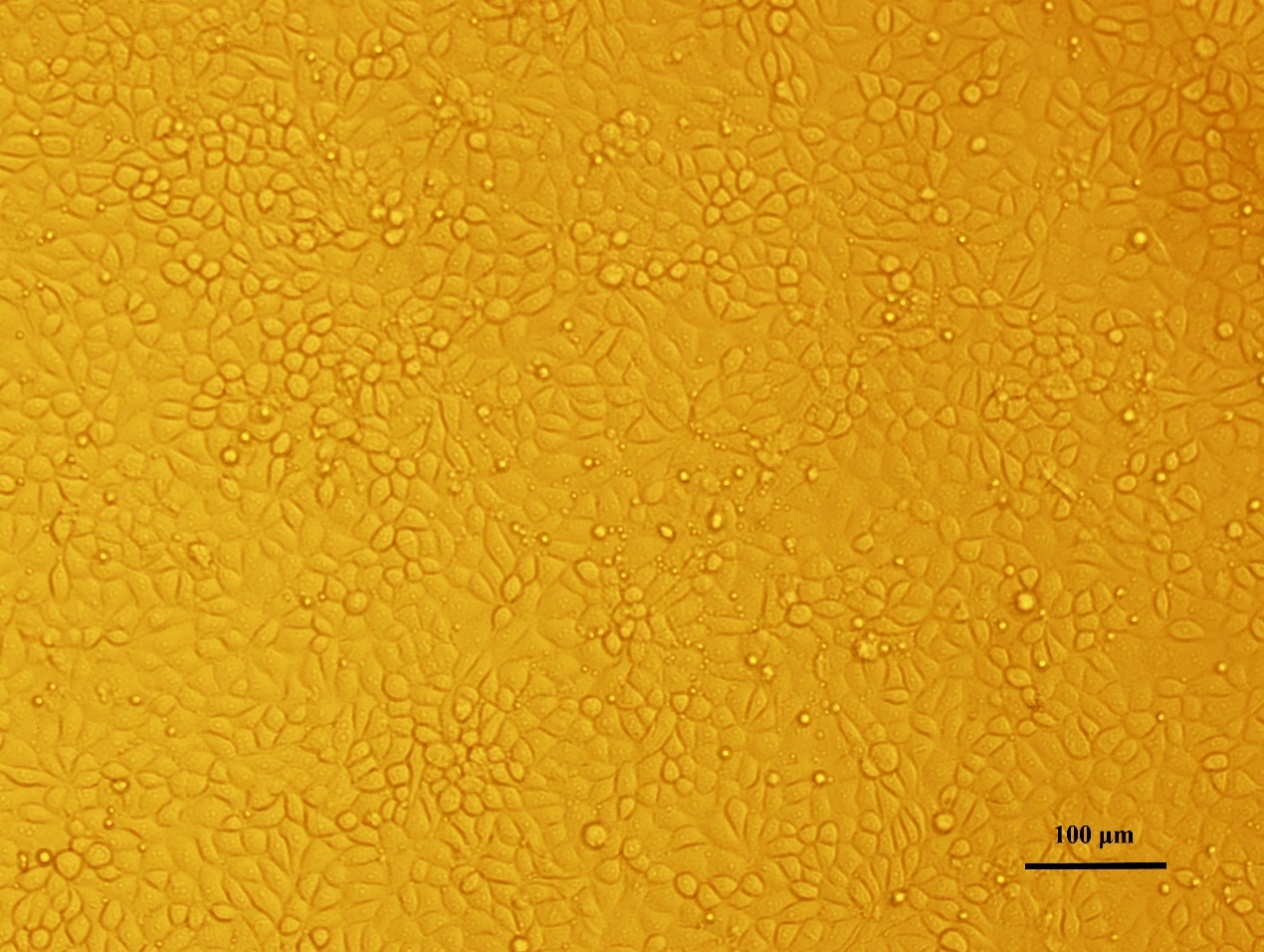


**(d)** Matrine 0.5 mg/mL + Osthole 0.01 mg/mL


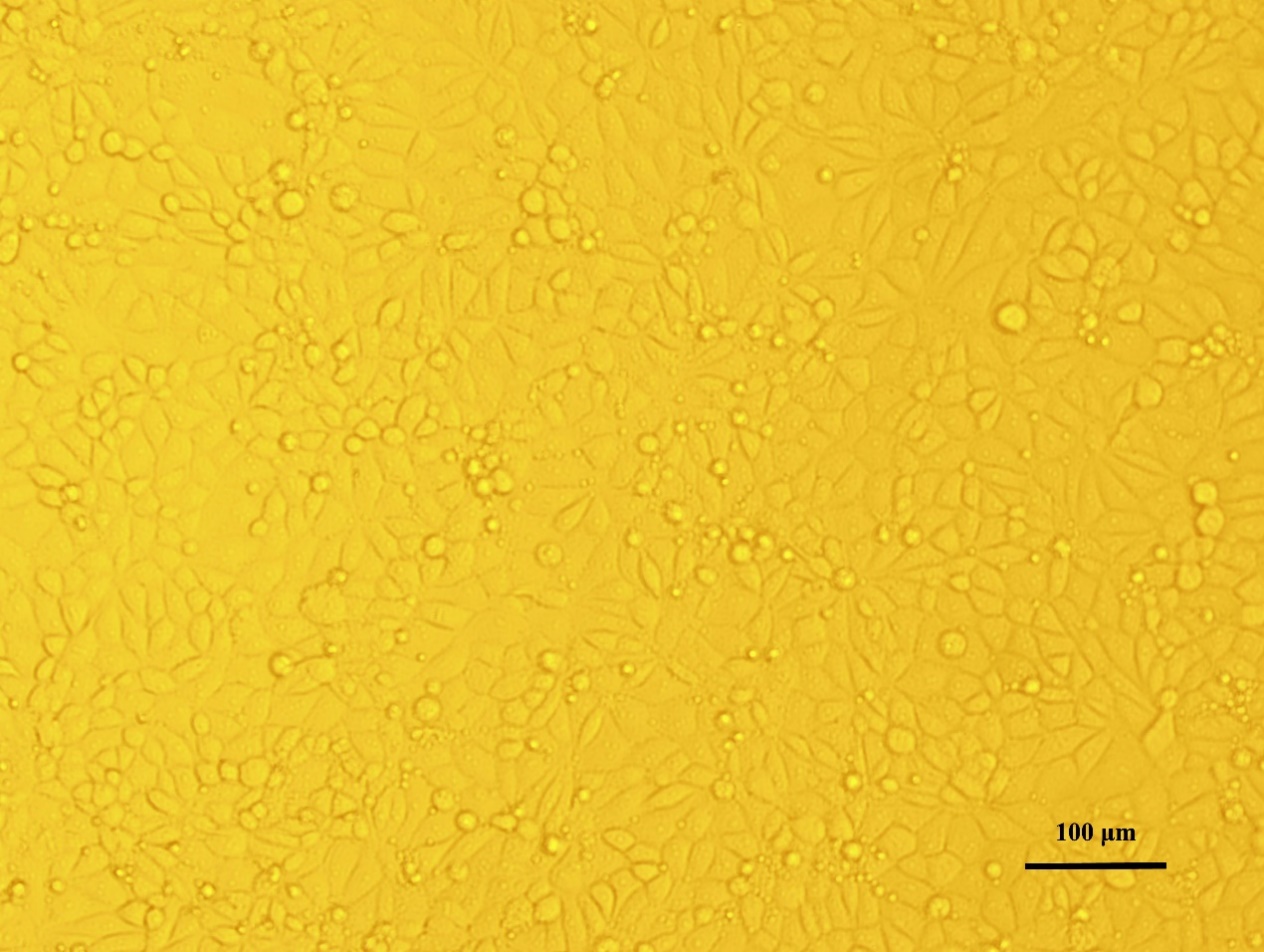


**(e)** Ribavirin 2 mg/mL


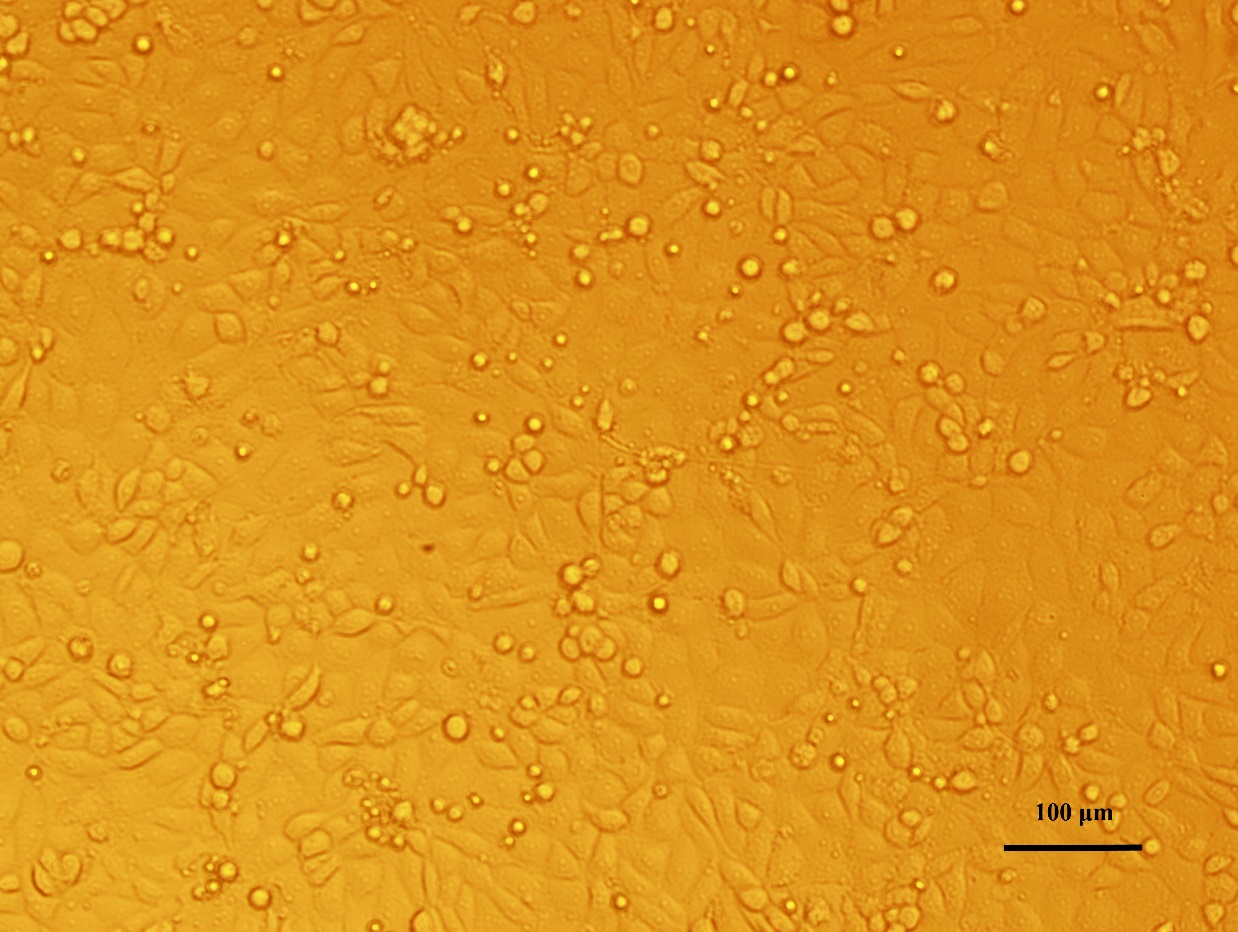


**(f)** Ribavirin 0.5 mg/mL


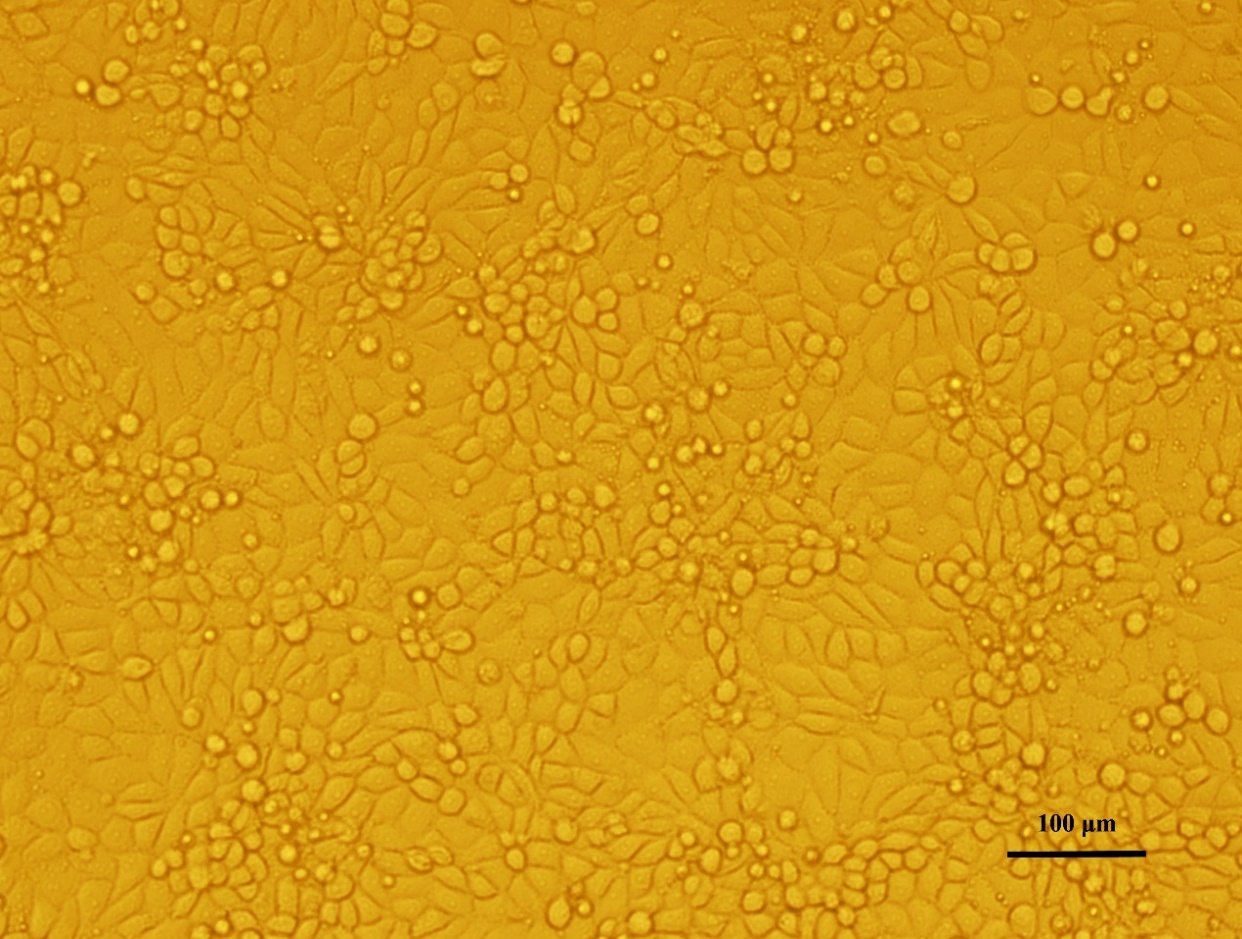

Supplement: Supplementary file 1 — Additional file 1. Original microscopic images of Fig. 1a. (a) Normal cell group, (b) 0.04 mg/mL Osthole, (c) 0.01 mg/mL Osthole, (d) 0.5 mg/mL Matrine + 0.01 mg/mL Osthole, (e) 2 mg/mL Ribavirin, and (f) 0.5 mg/mL Ribavirin. [file 12866_2020_1986_MOESM1_ESM.docx]
