# Supplementary material for: The combined usage of Matrine and Osthole inhibited endoplasmic reticulum apoptosis induced by PCV2
Source: BMC Microbiol. 2020 Oct 12;20:303. doi: 10.1186/s12866-020-01986-2 (PMC7549248; doi:10.1186/s12866-020-01986-2)

**The original IFA images of Fig. 4D**

**
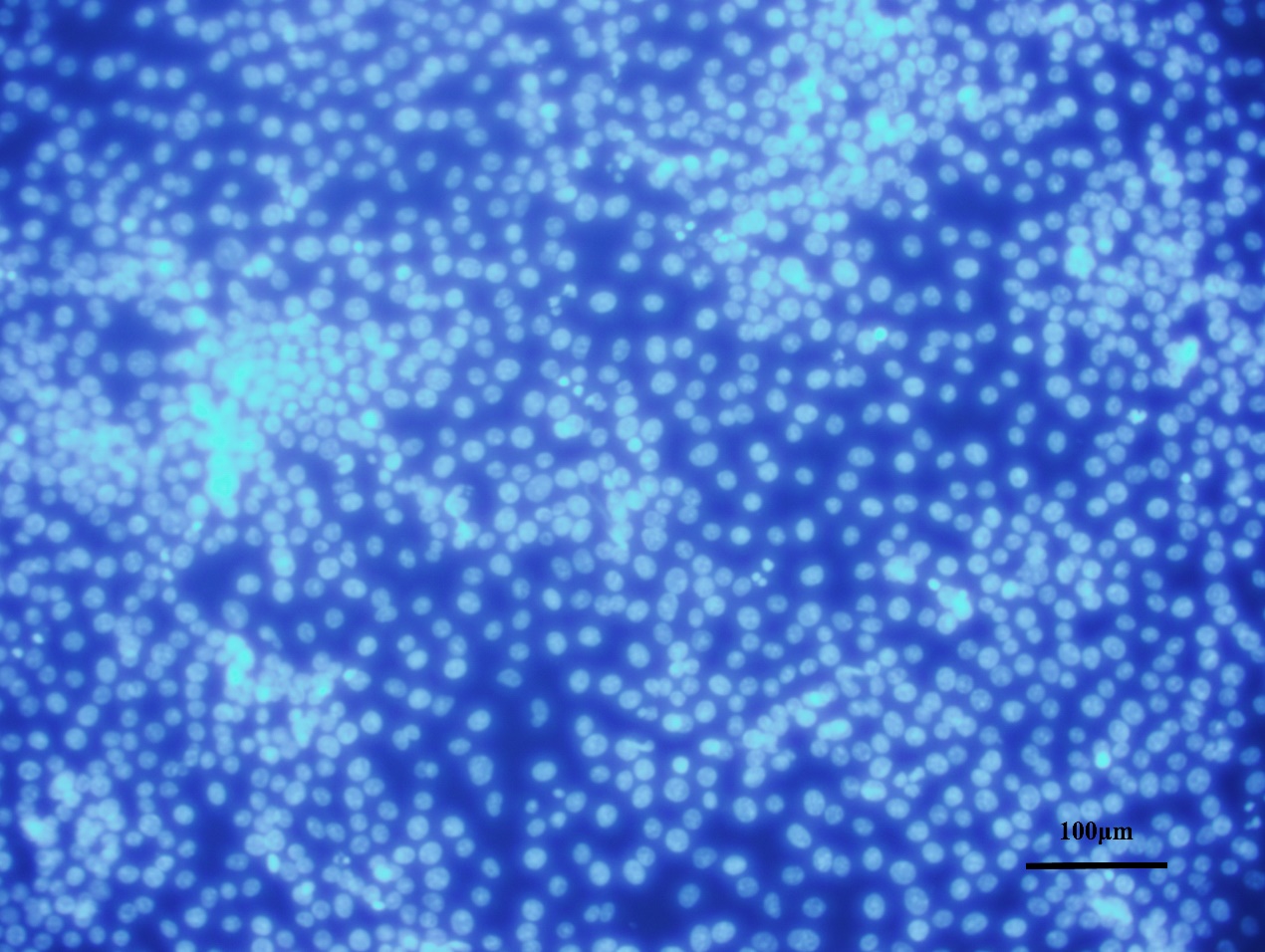
(a)** Cell group

**
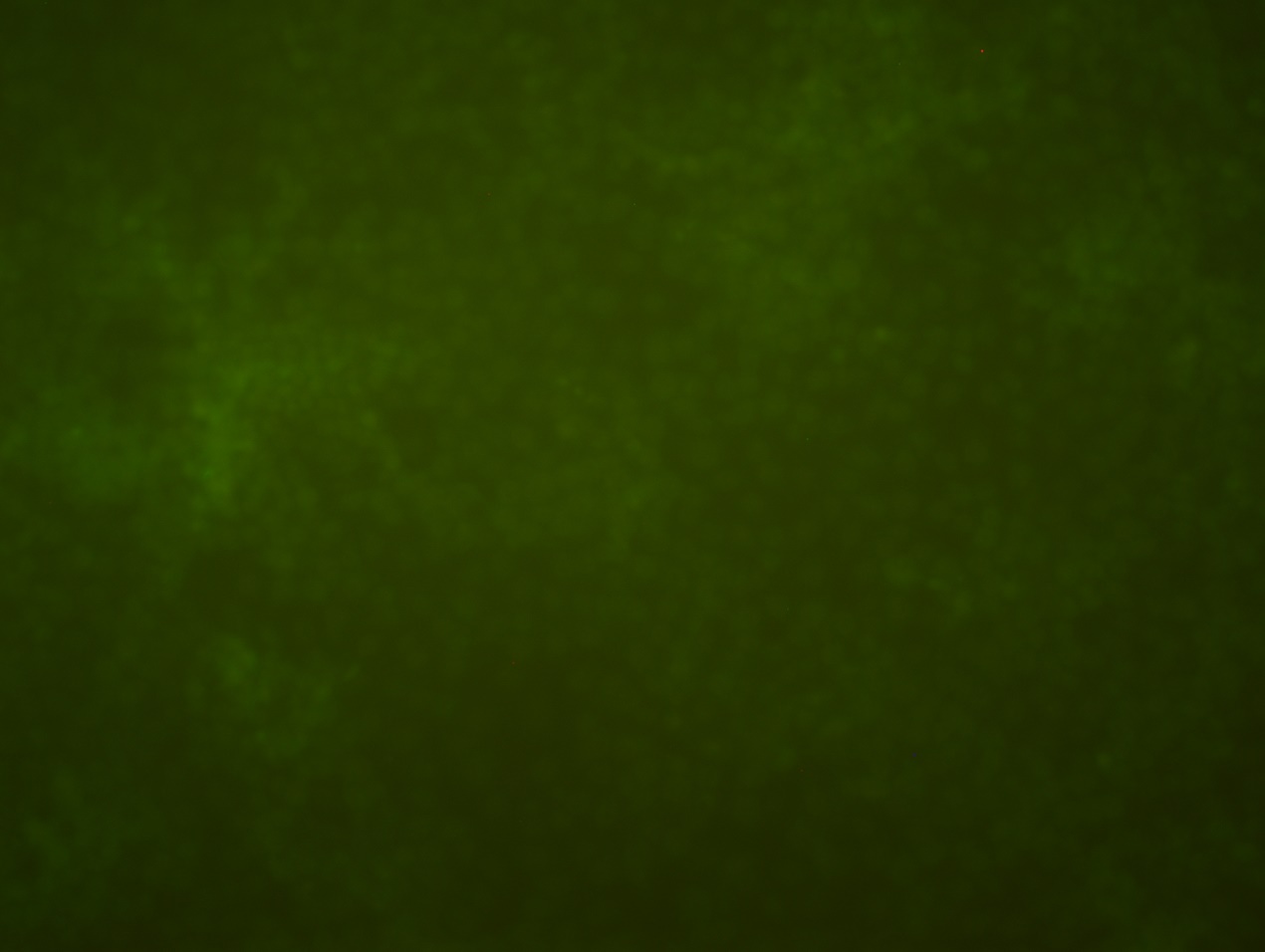

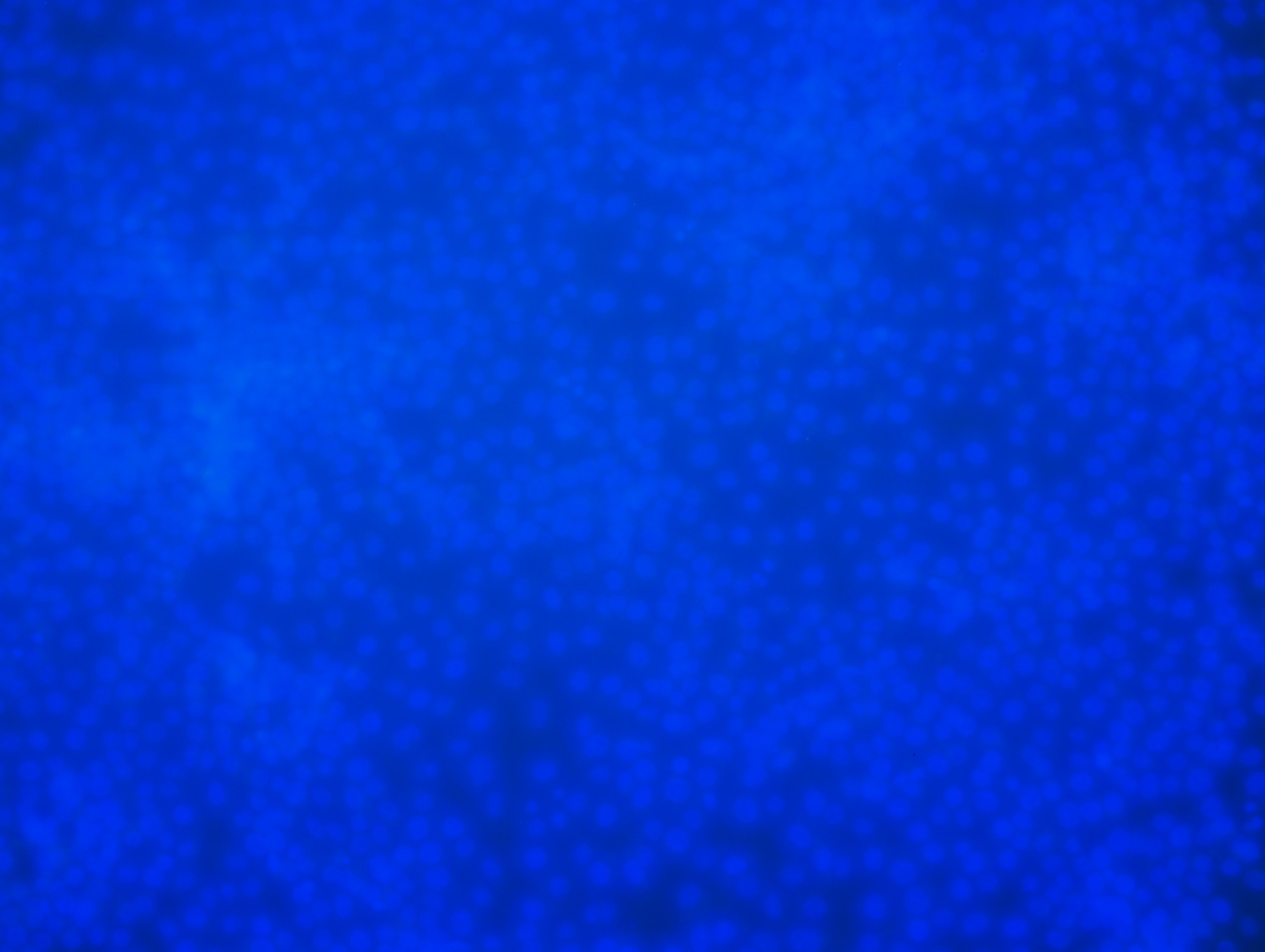
**

**(b)** Virus group


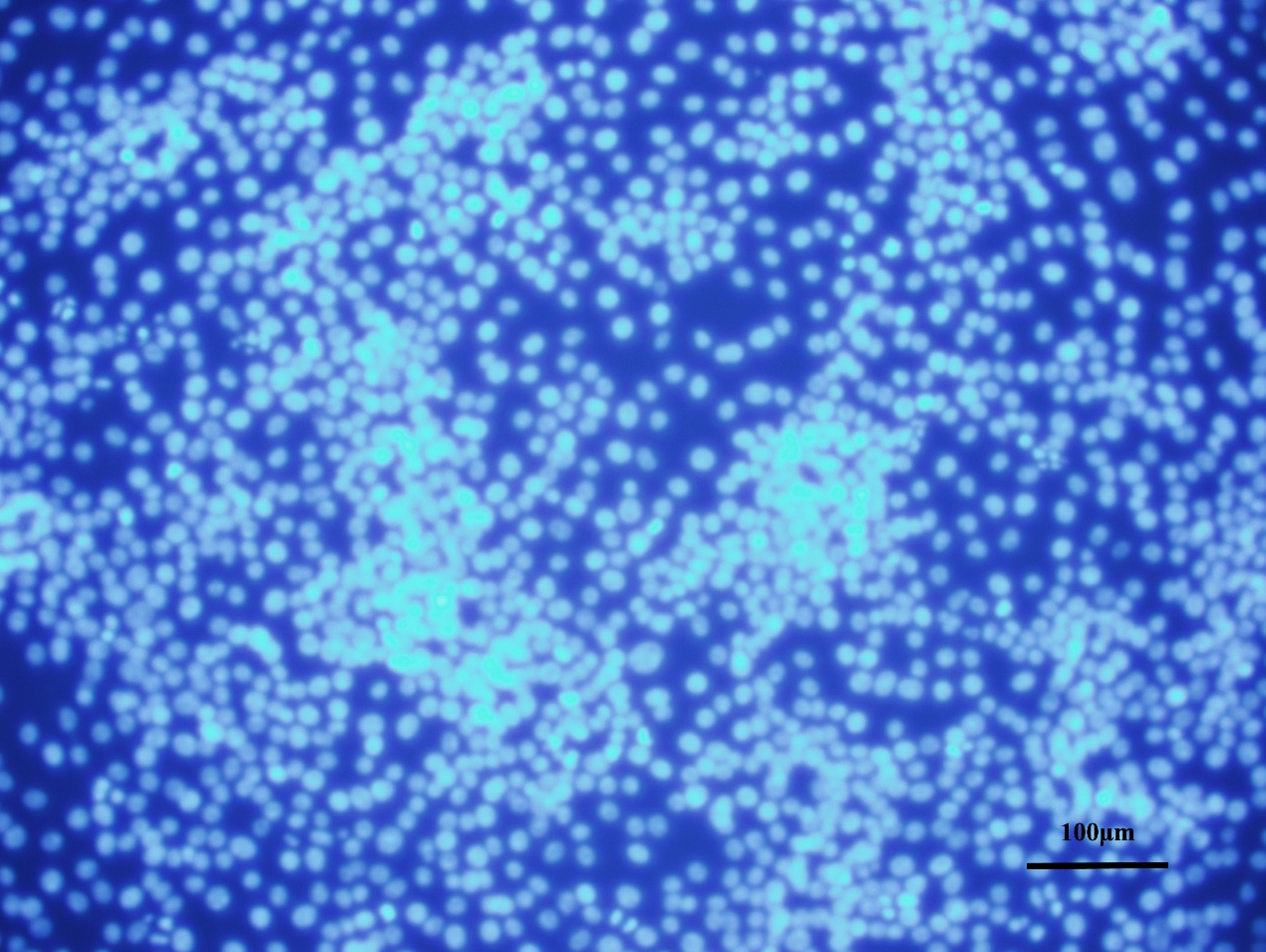


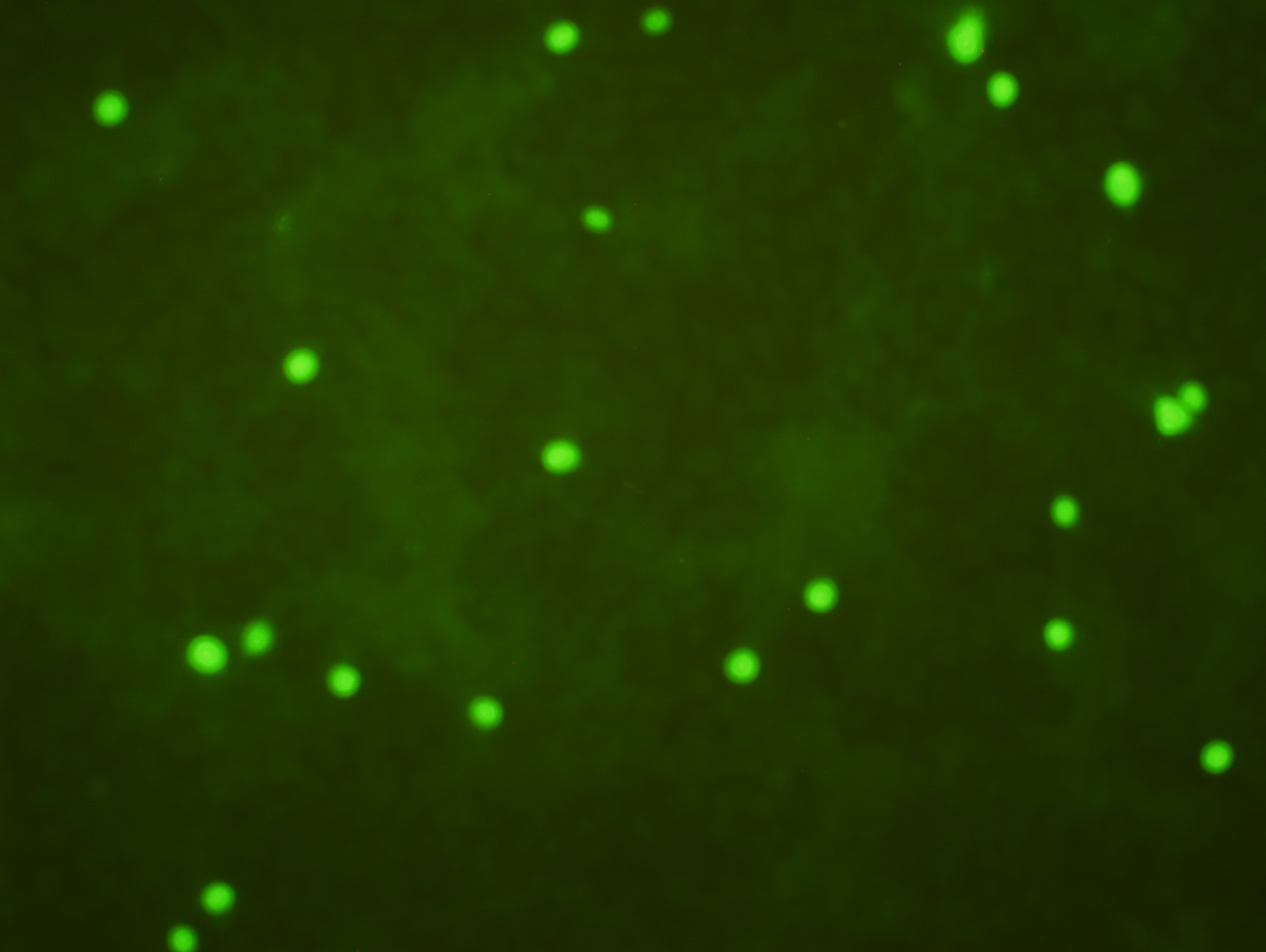


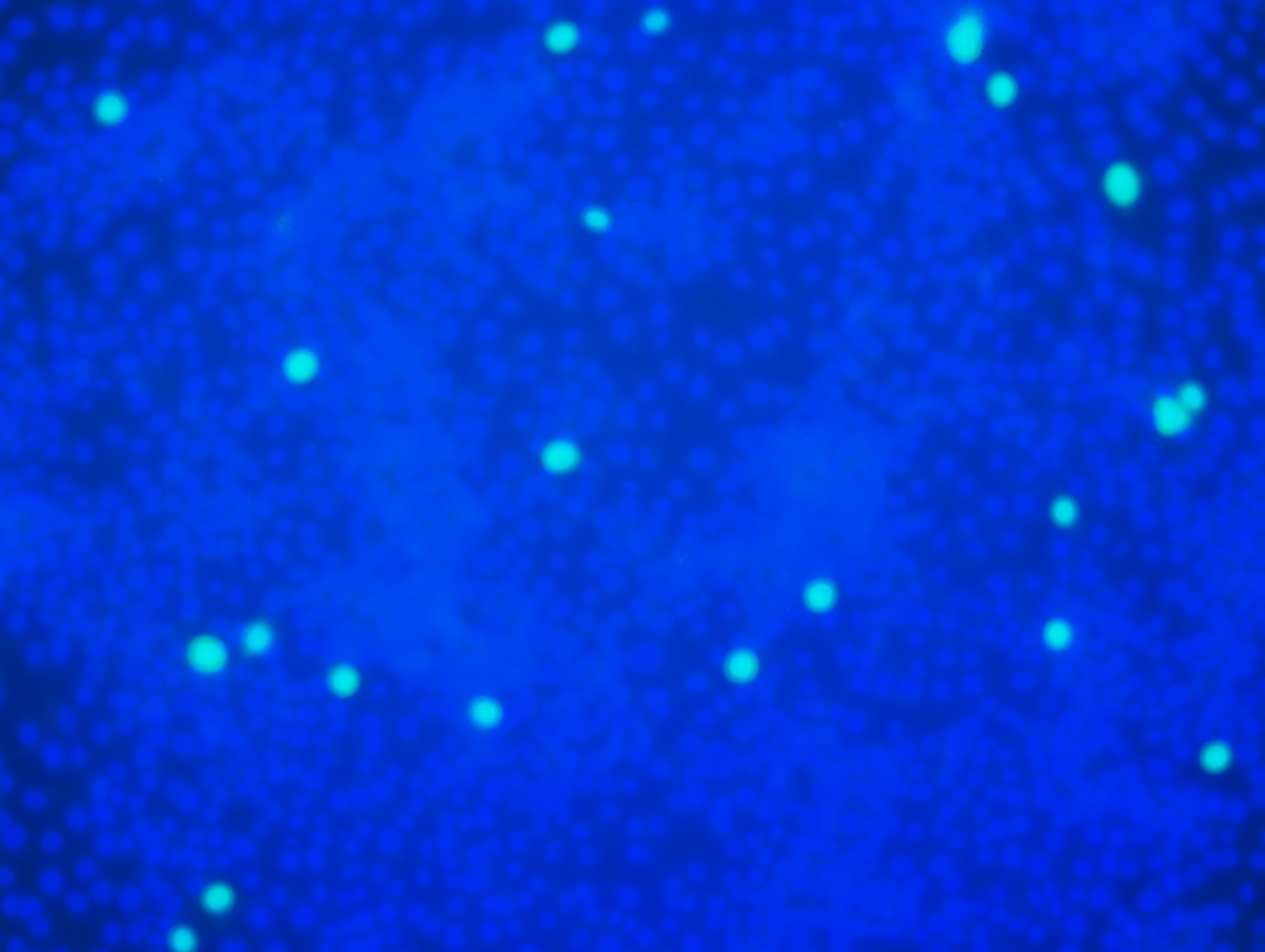


**(c)** Matrine 0.5mg/mL+ Osthole 0.01mg/mL


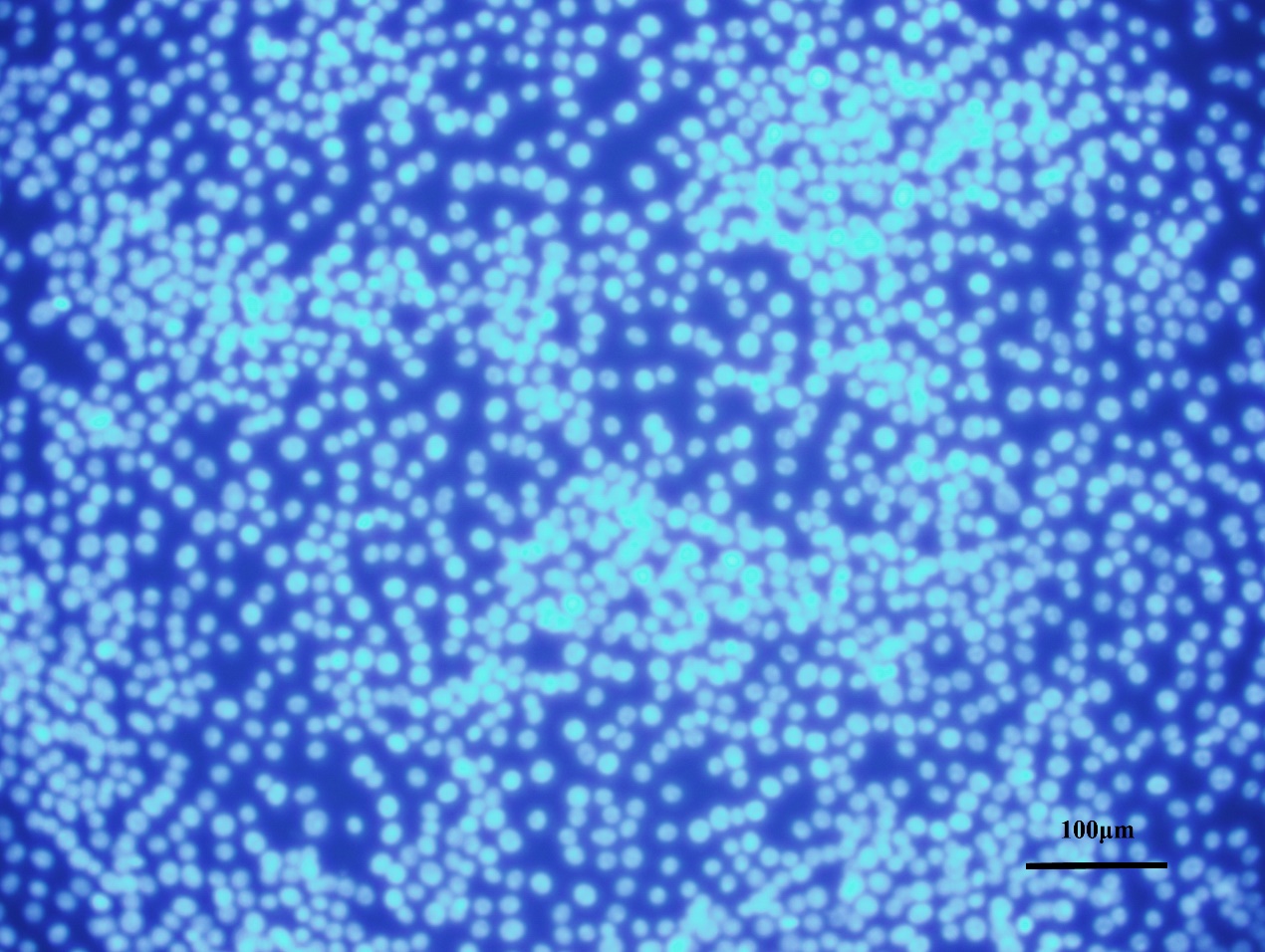

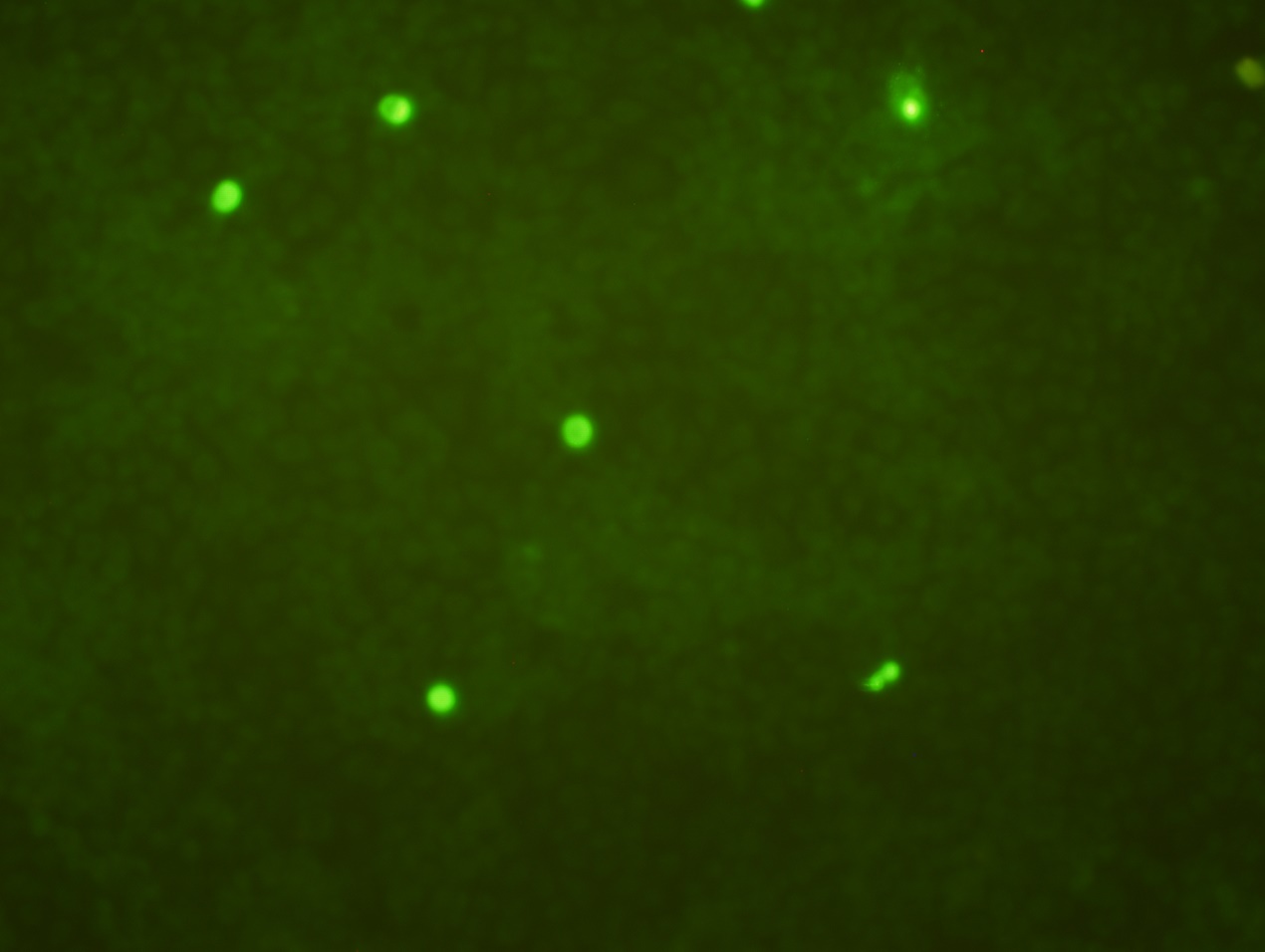

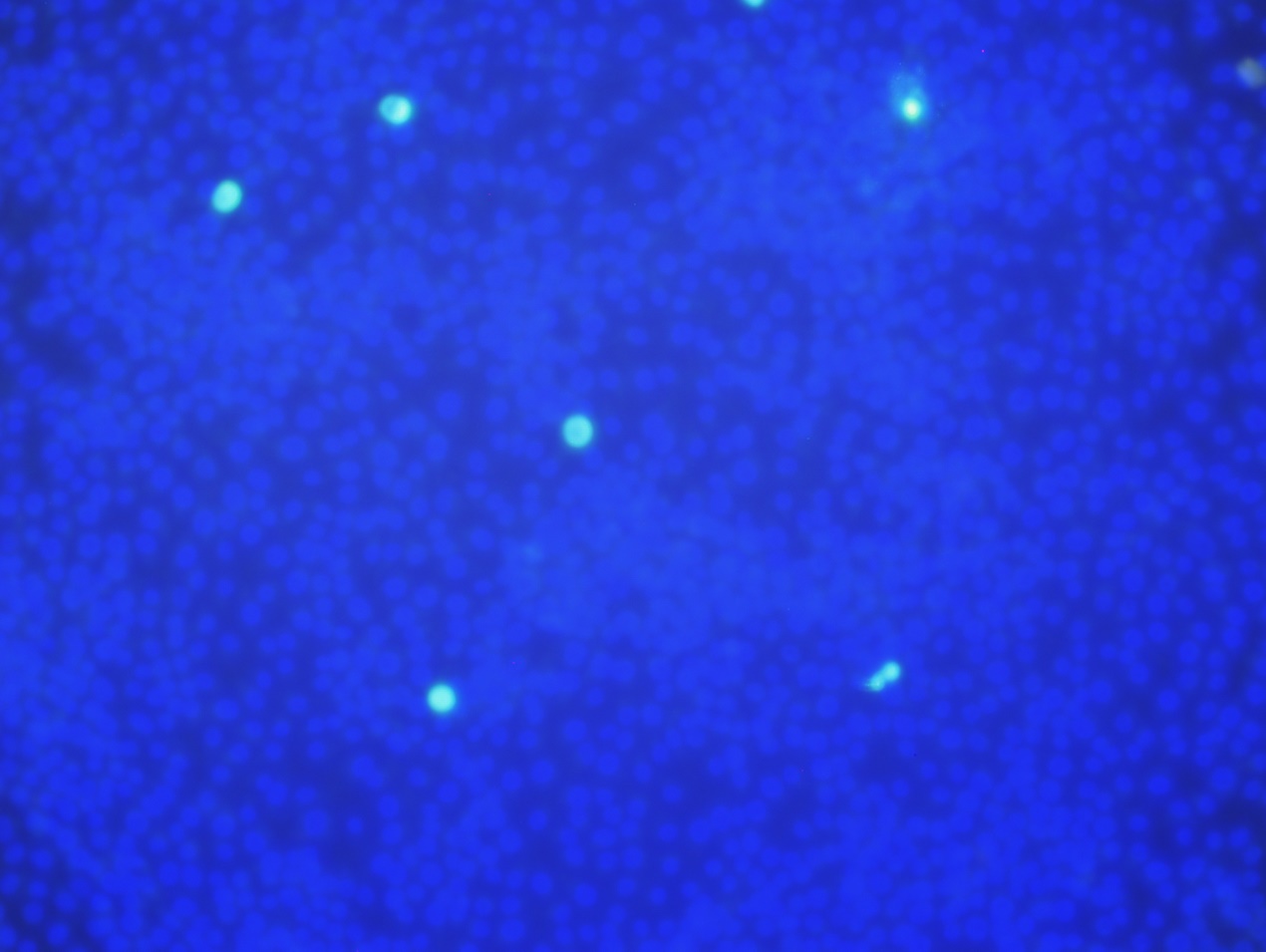


**(d)** Matrine 0.25mg/mL + Osthole 0.01mg/mL


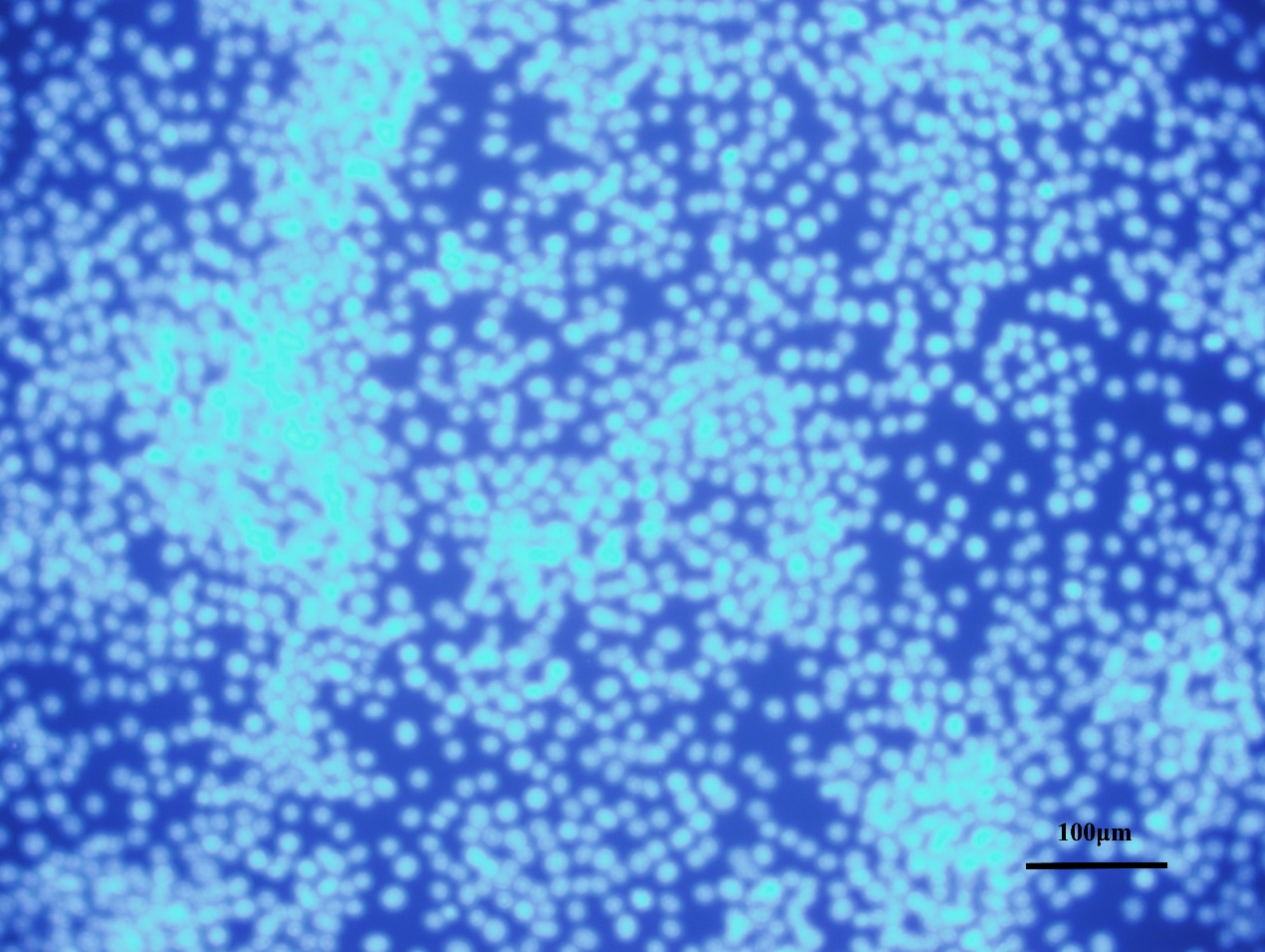

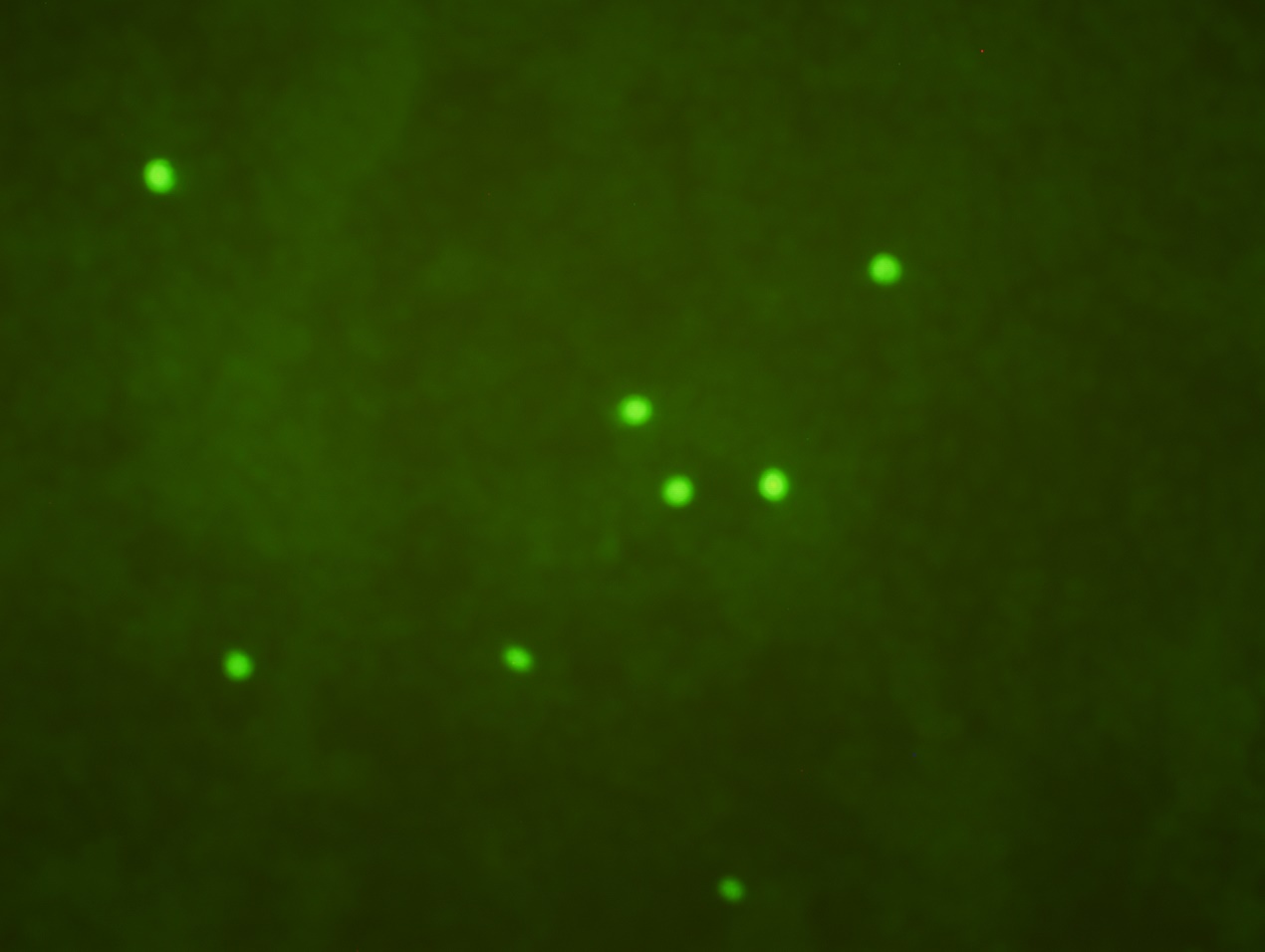

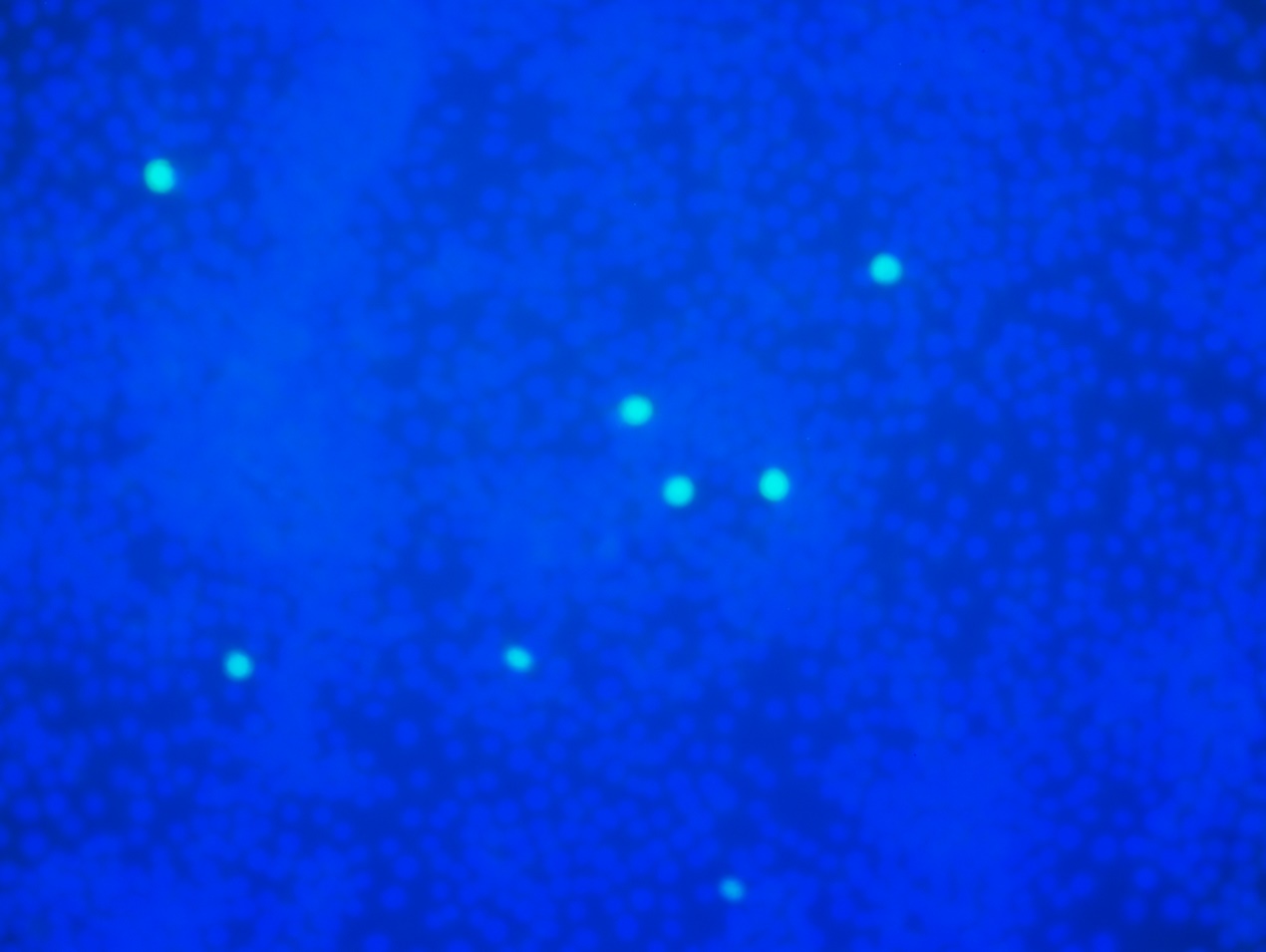


**(e)** Matrine 0.125mg/mL + Osthole 0.01mg/mL


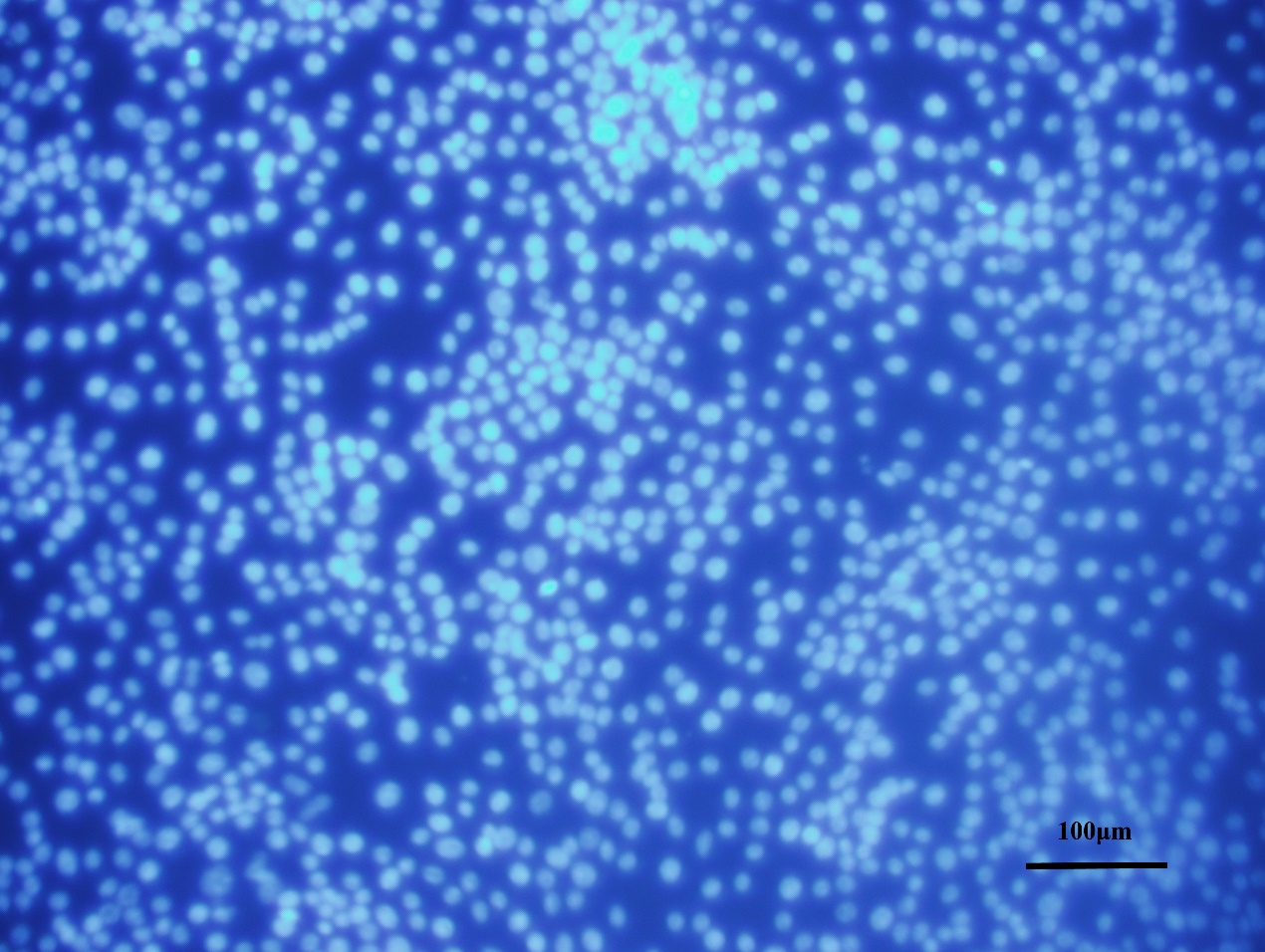

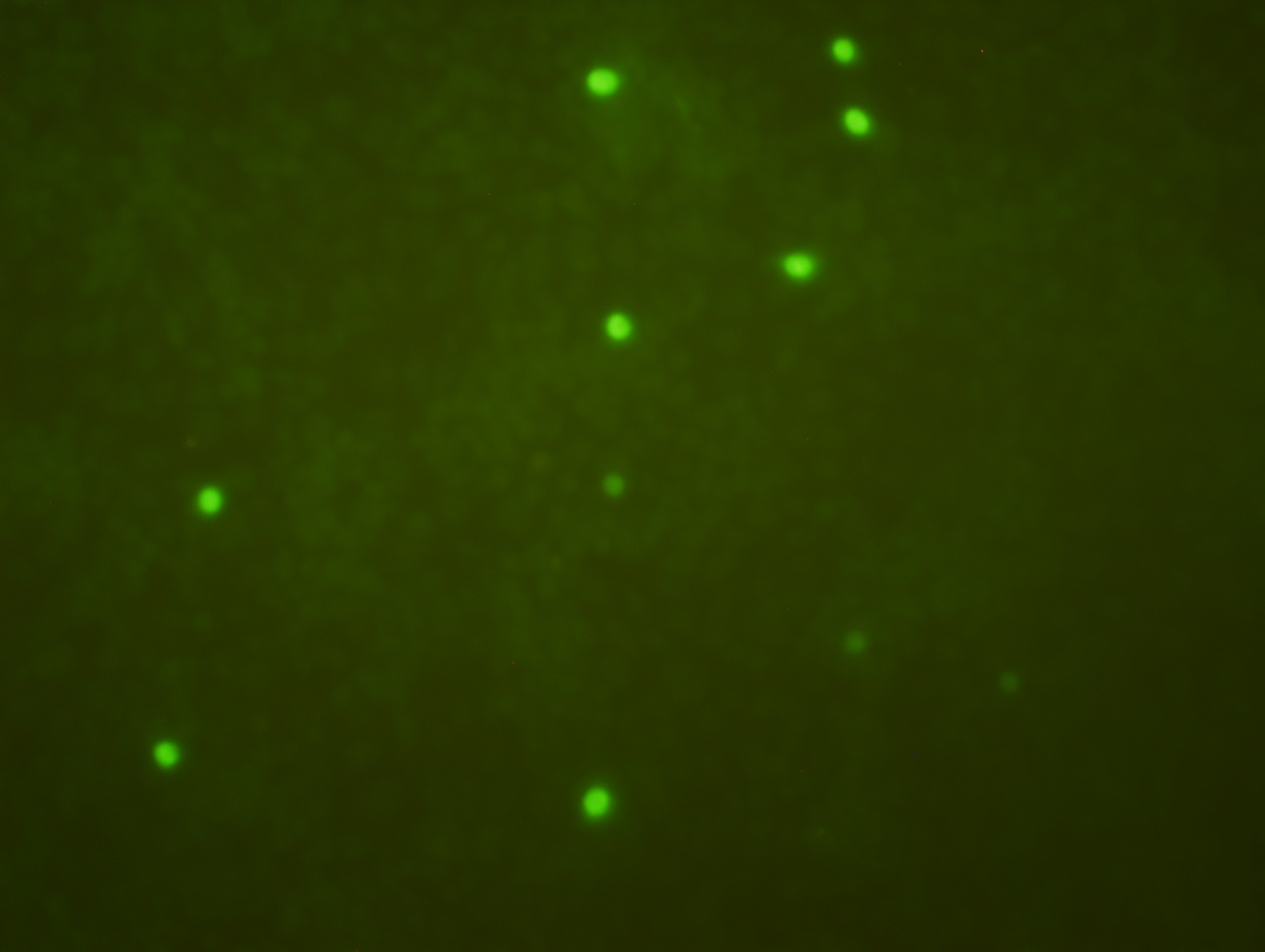

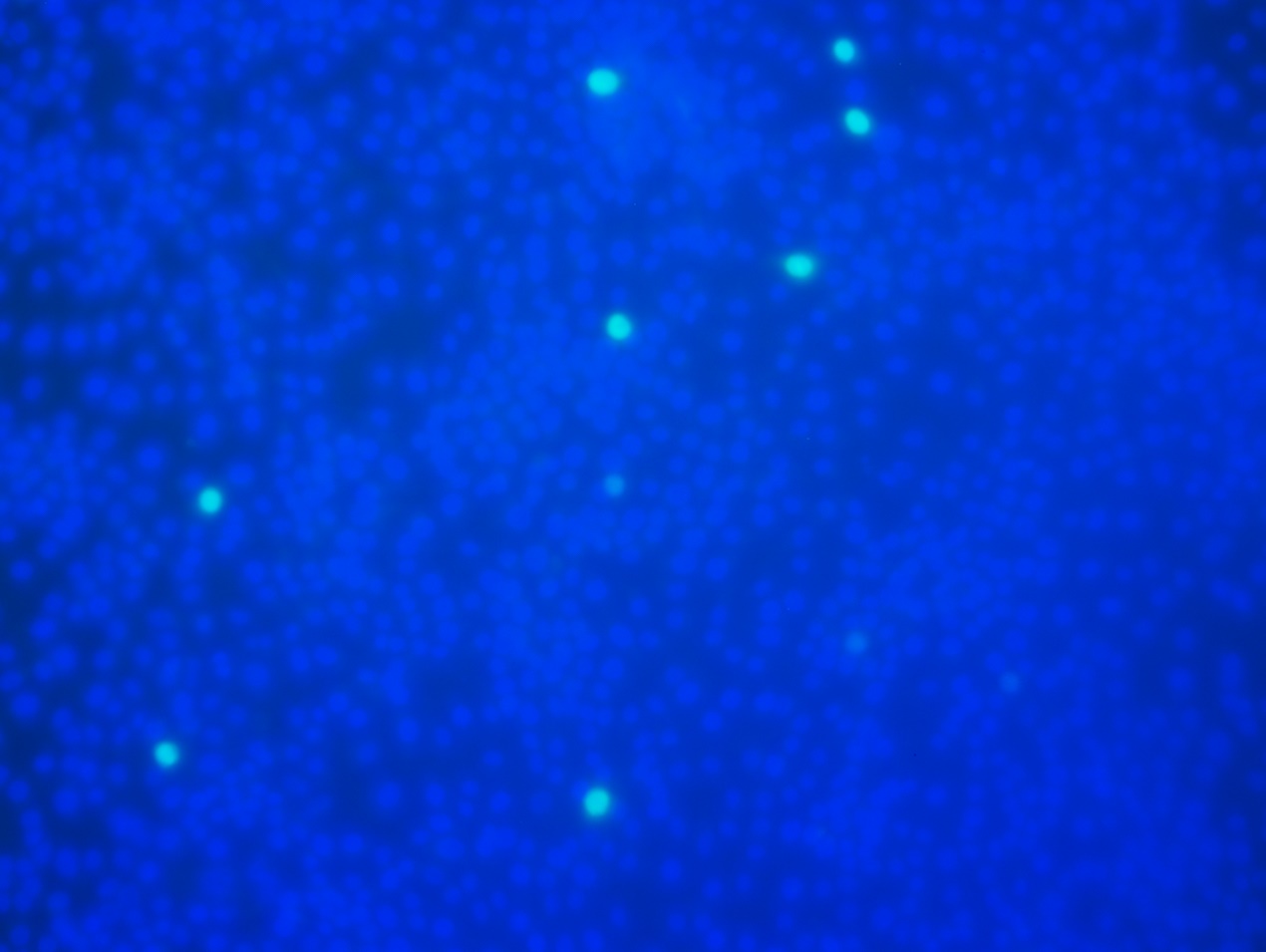


**(f)** Matrine 0.5 mg/mL


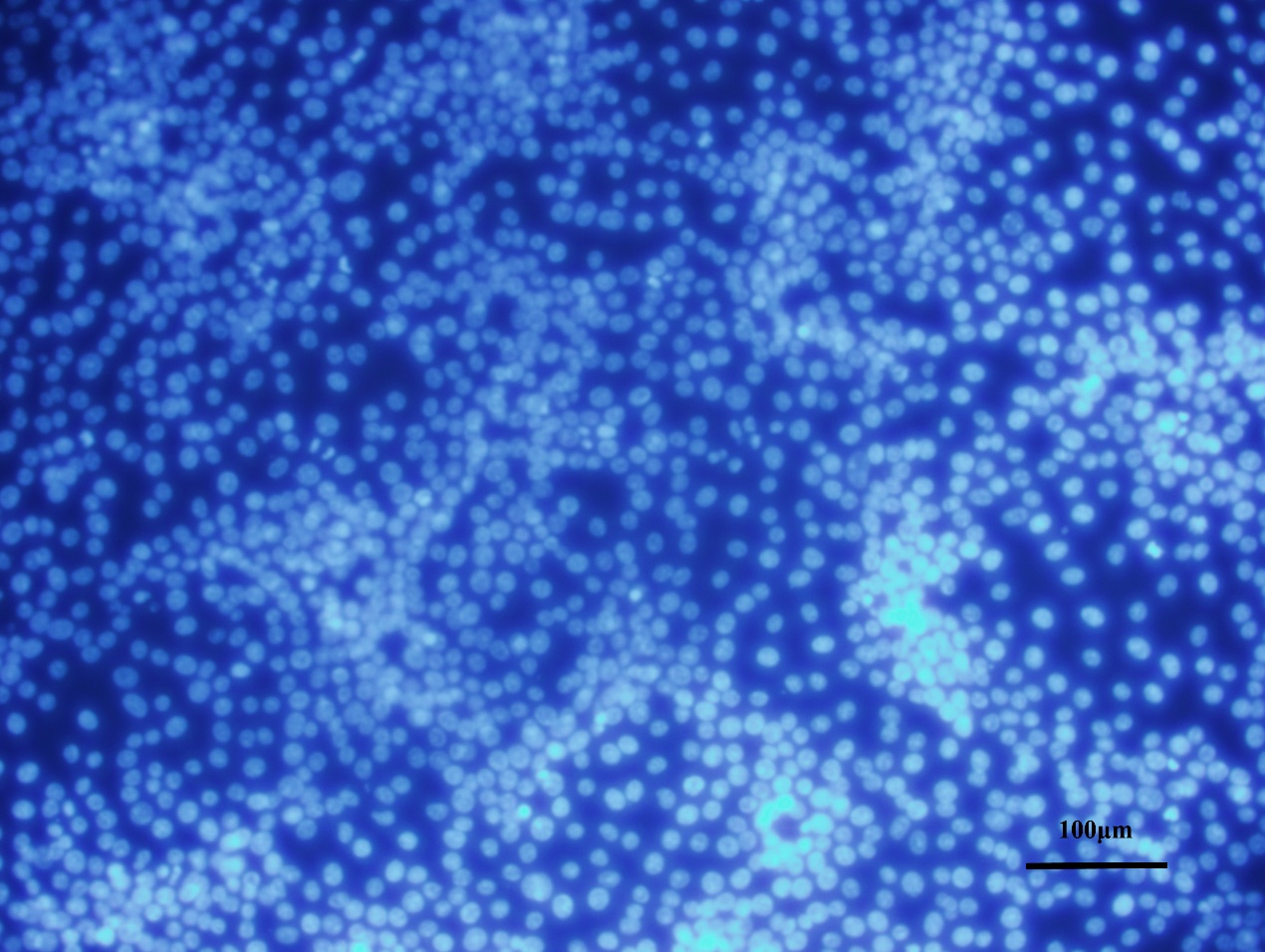

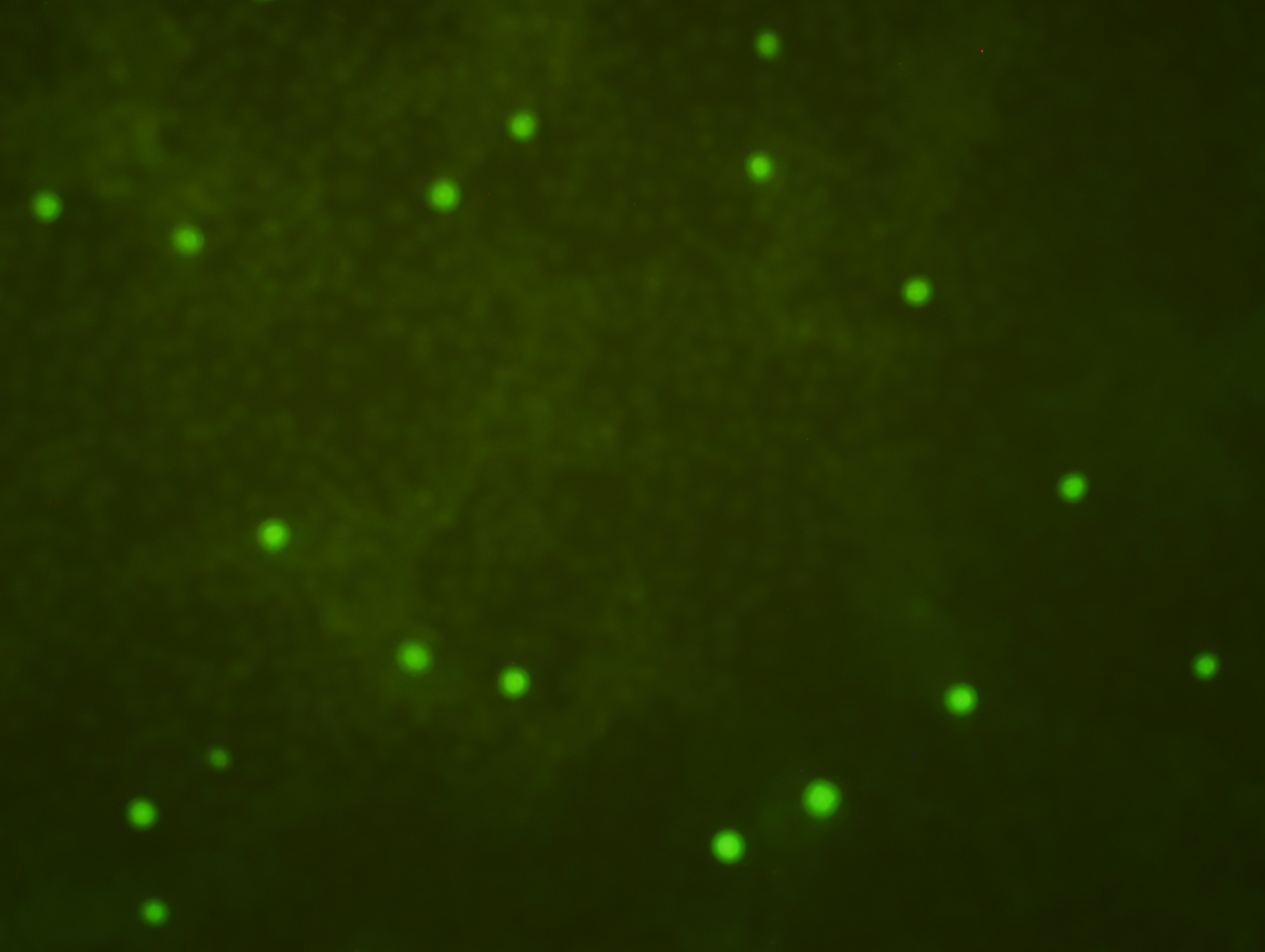

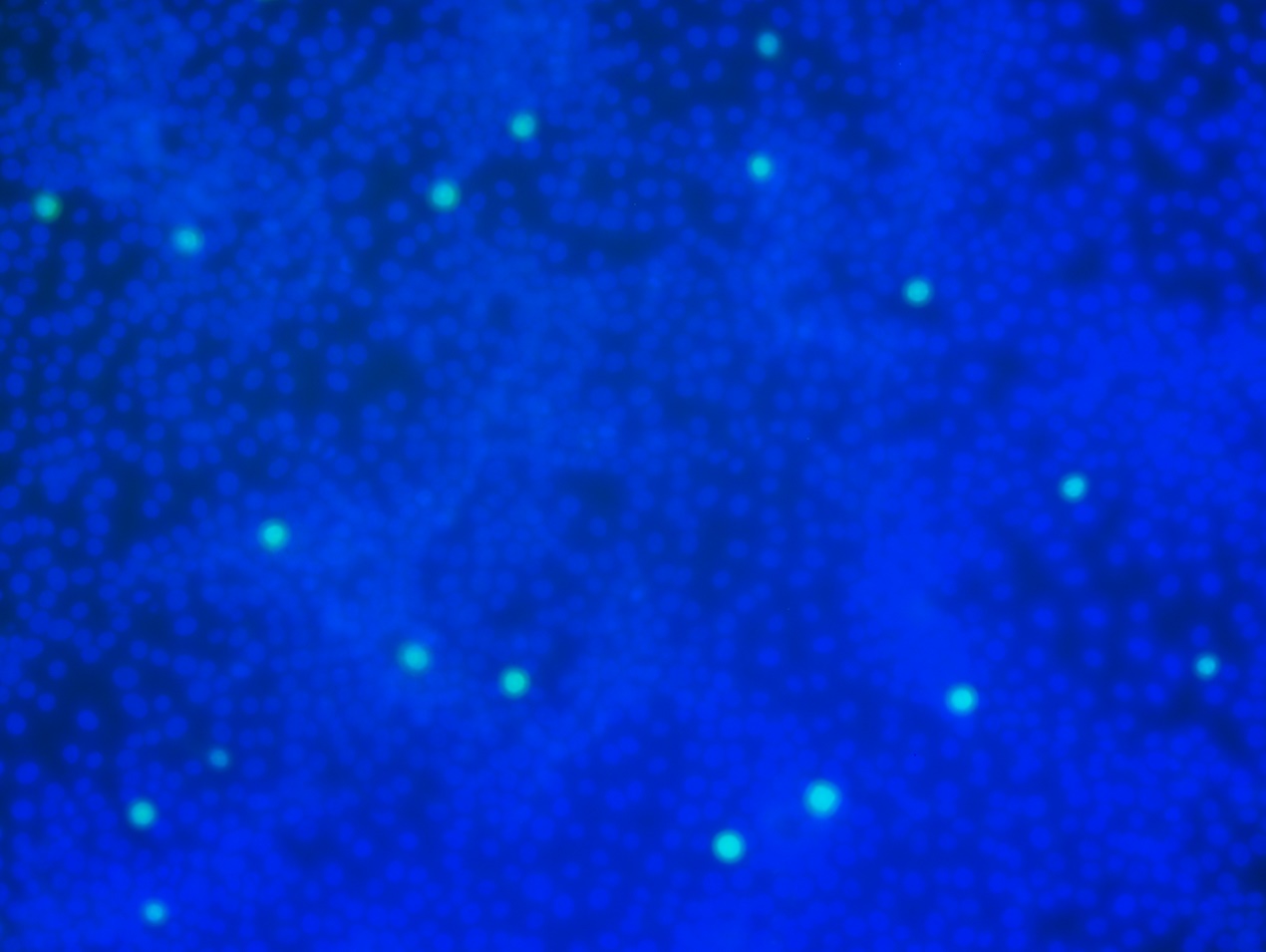


**(g)** Osthole 0.01 mg/mL


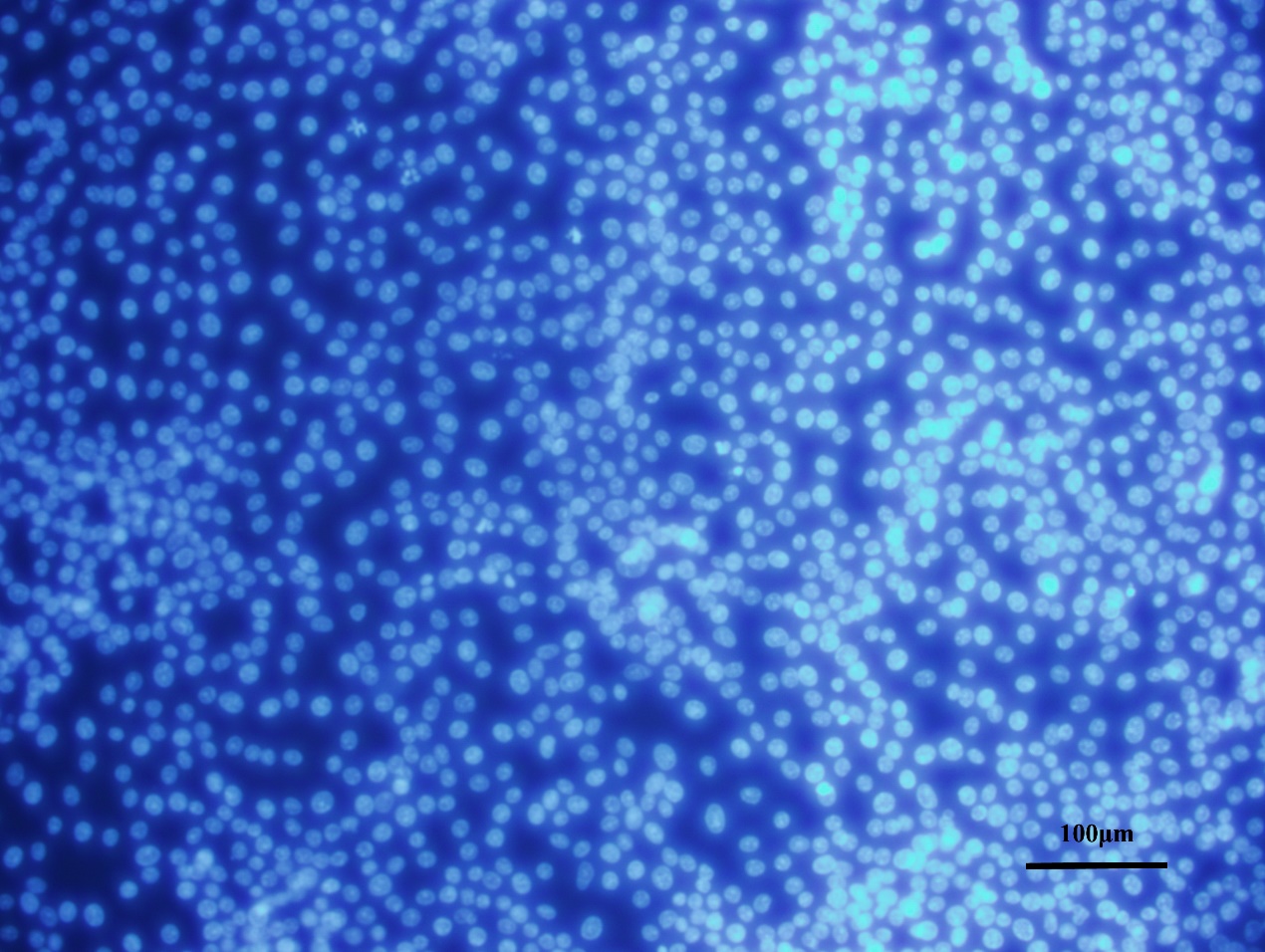

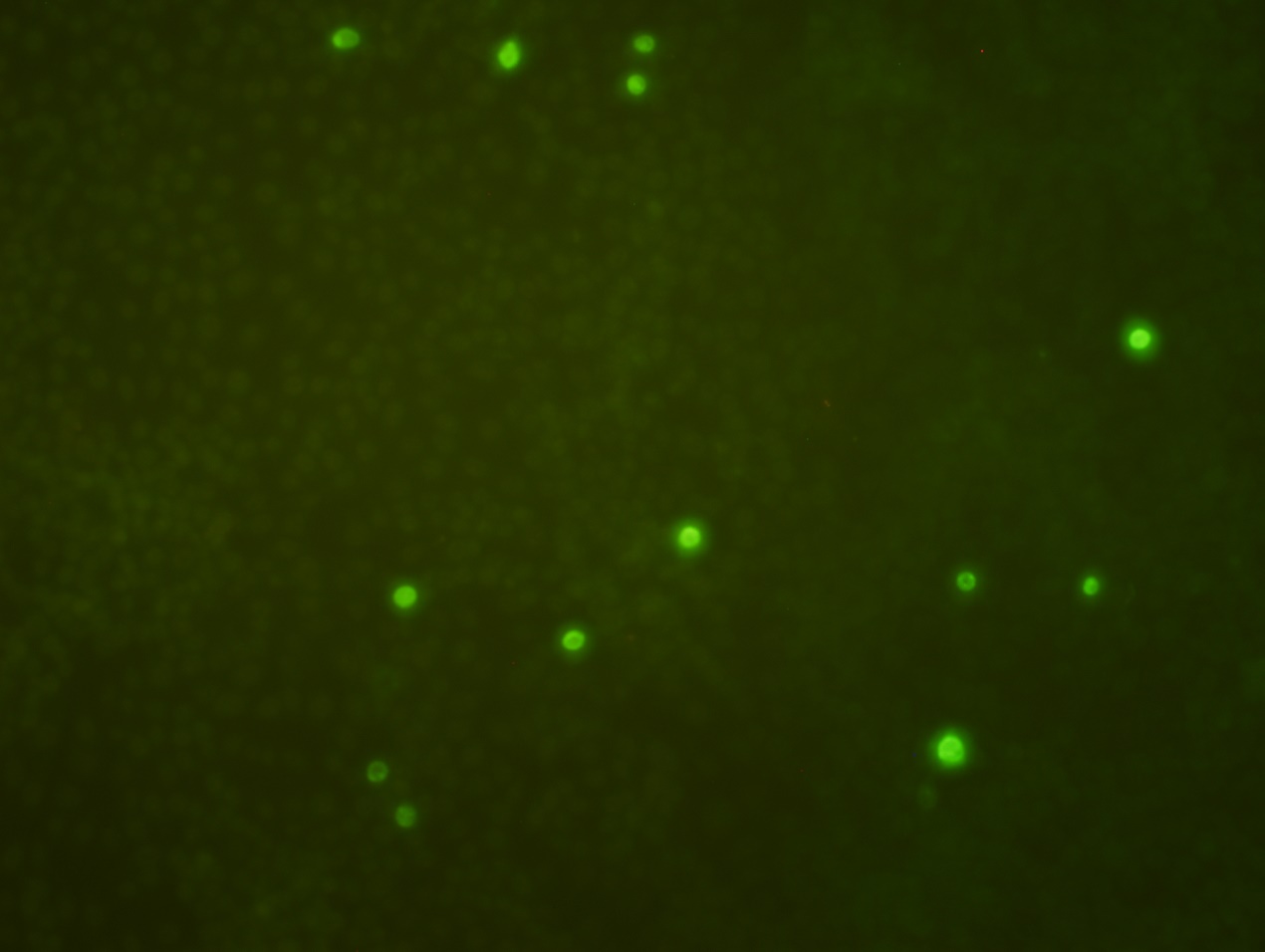

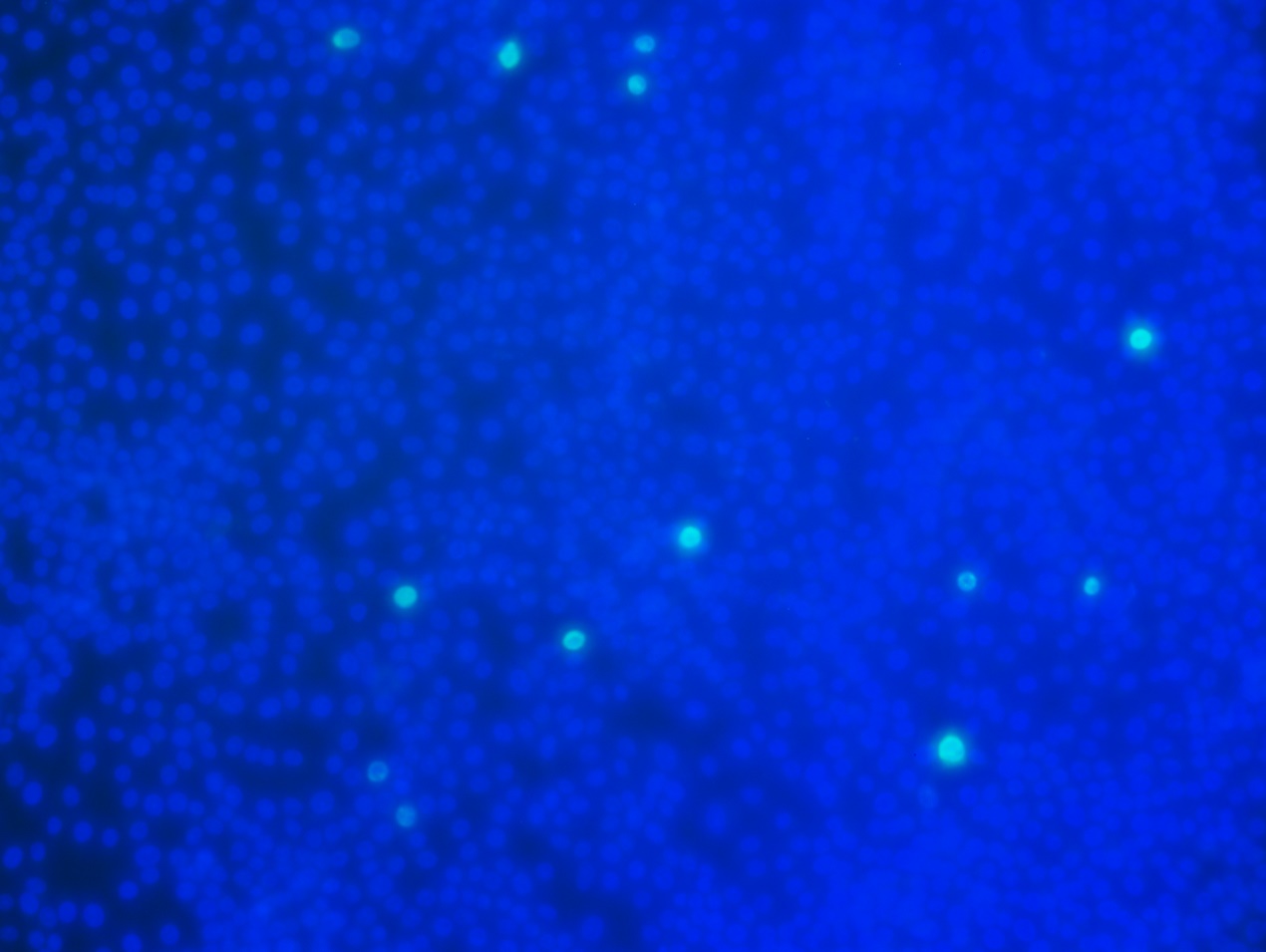


**(h)** Ribavirin 0.5 mg/mL


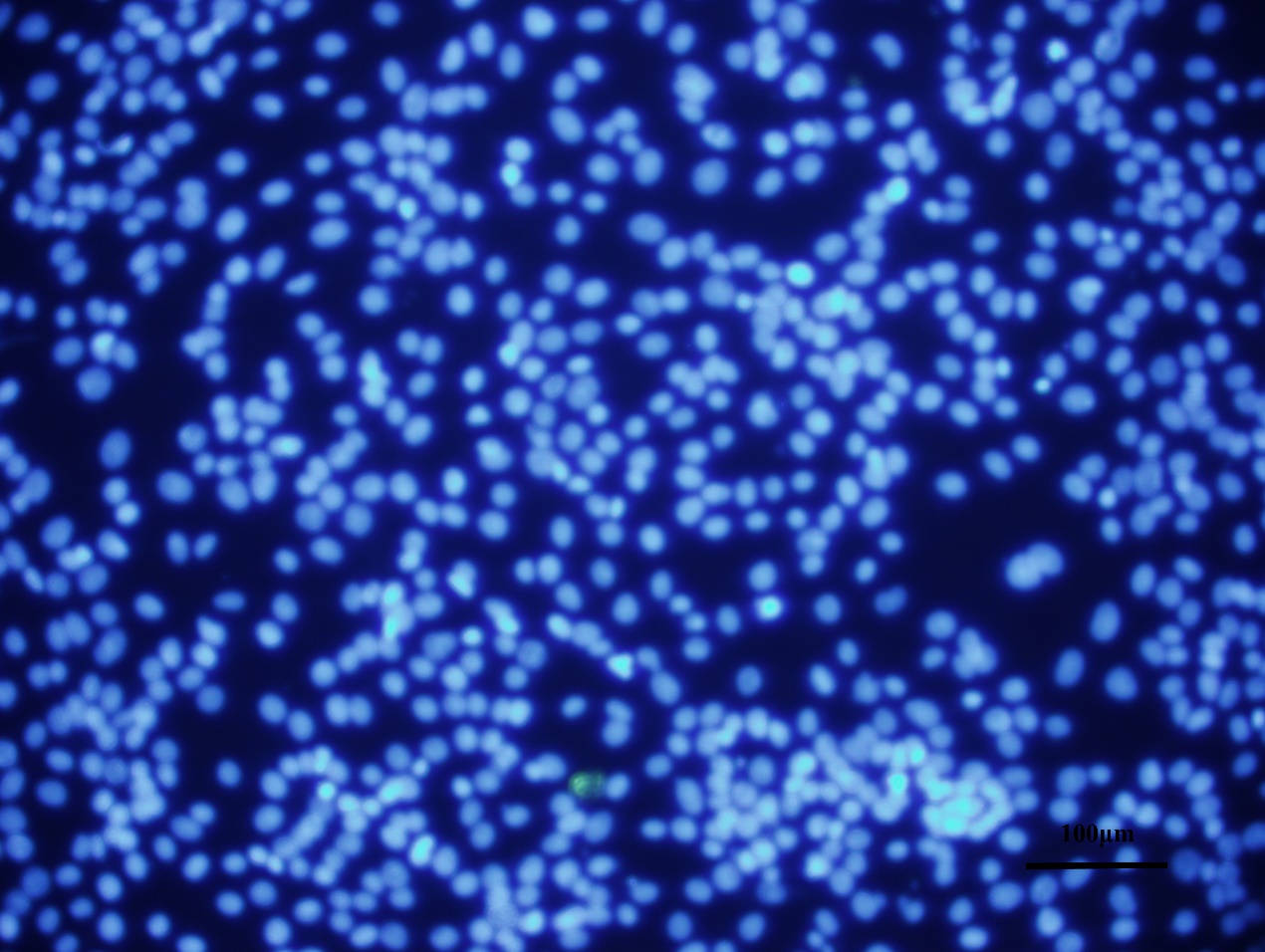

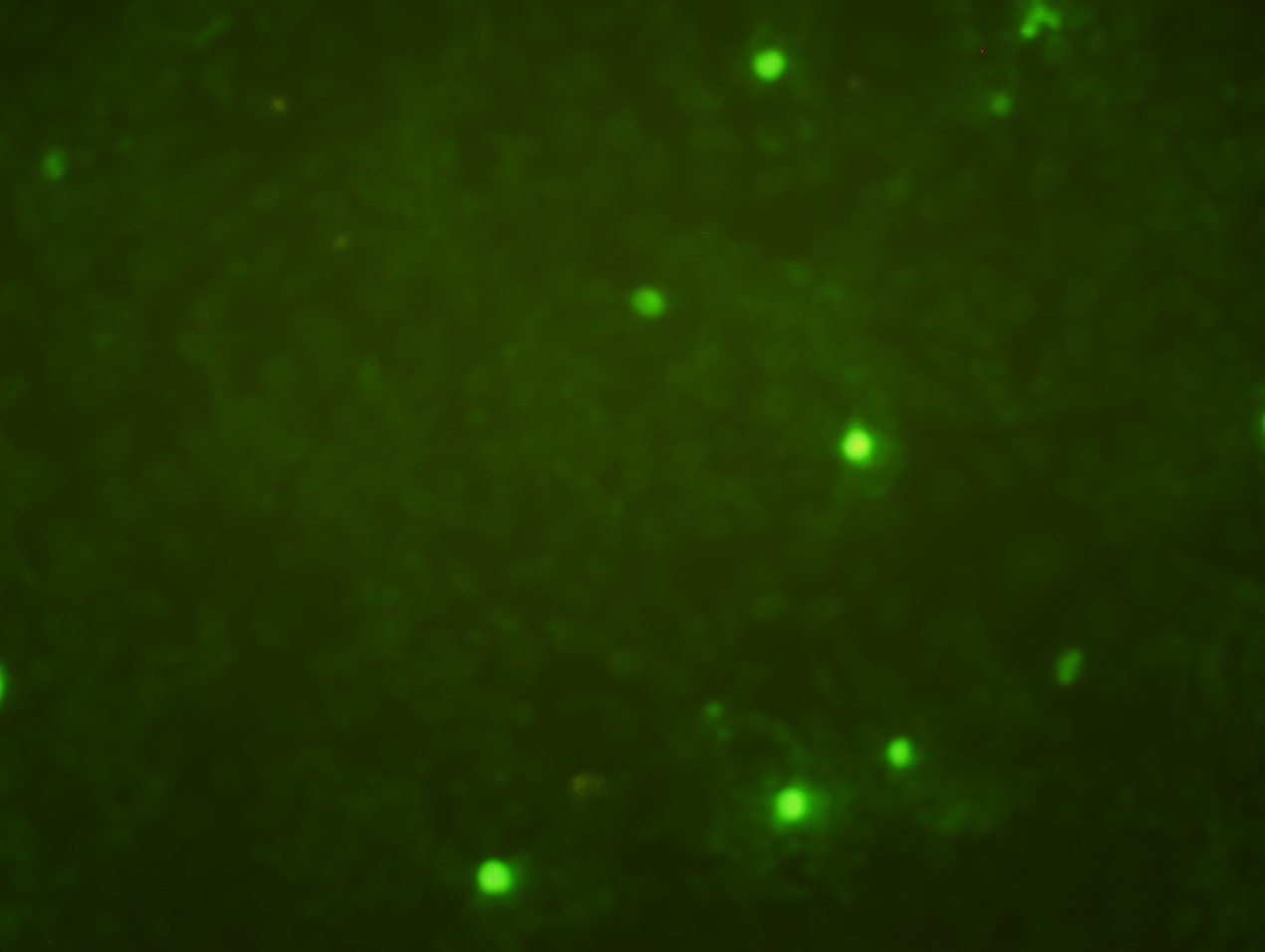

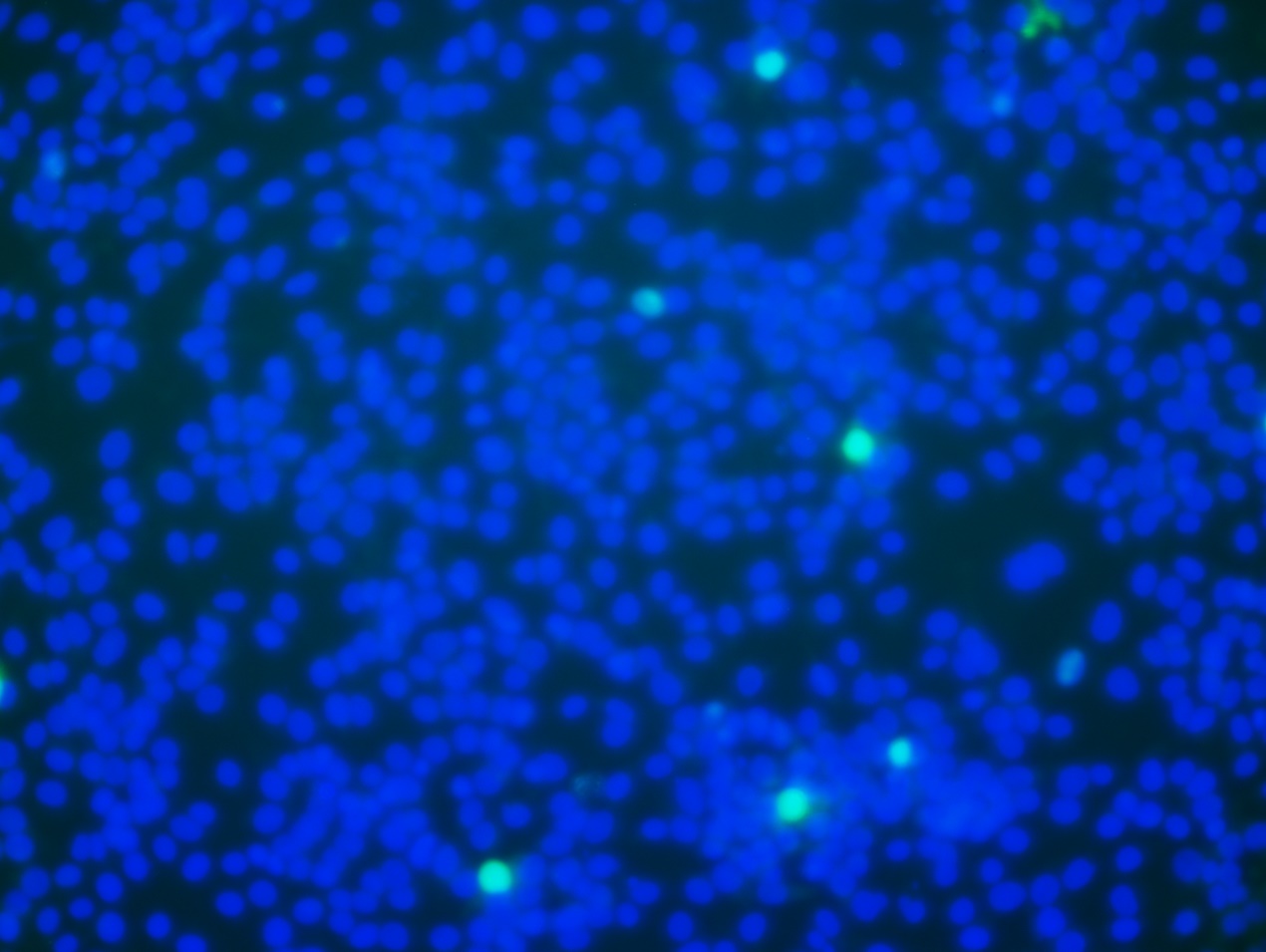

Supplement: Supplementary file 2 — Additional file 2. Original IFA images of Fig. 3d. (a) Cell group, (b) virus group, (c) 0.5 mg/mL Matrine + 0.01 mg/mL Osthole, (d) 0.25 mg/mL Matrine + 0.01 mg/mL Osthole, (e) 0.125 mg/mL Matrine + 0.01 mg/mL Osthole, (f) 0.5 mg/mL Matrine, (g) 0.01 mg/mL Osthole, and (h) 0.5 mg/mL Ribavirin. [file 12866_2020_1986_MOESM2_ESM.docx]
