# Supplementary figures and images for: The combined usage of Matrine and Osthole inhibited endoplasmic reticulum apoptosis induced by PCV2
Source: BMC Microbiol. 2020 Oct 12;20:303. doi: 10.1186/s12866-020-01986-2 (PMC7549248; doi:10.1186/s12866-020-01986-2)

**Original blot image of Fig. 4F**

**(a)** Cap


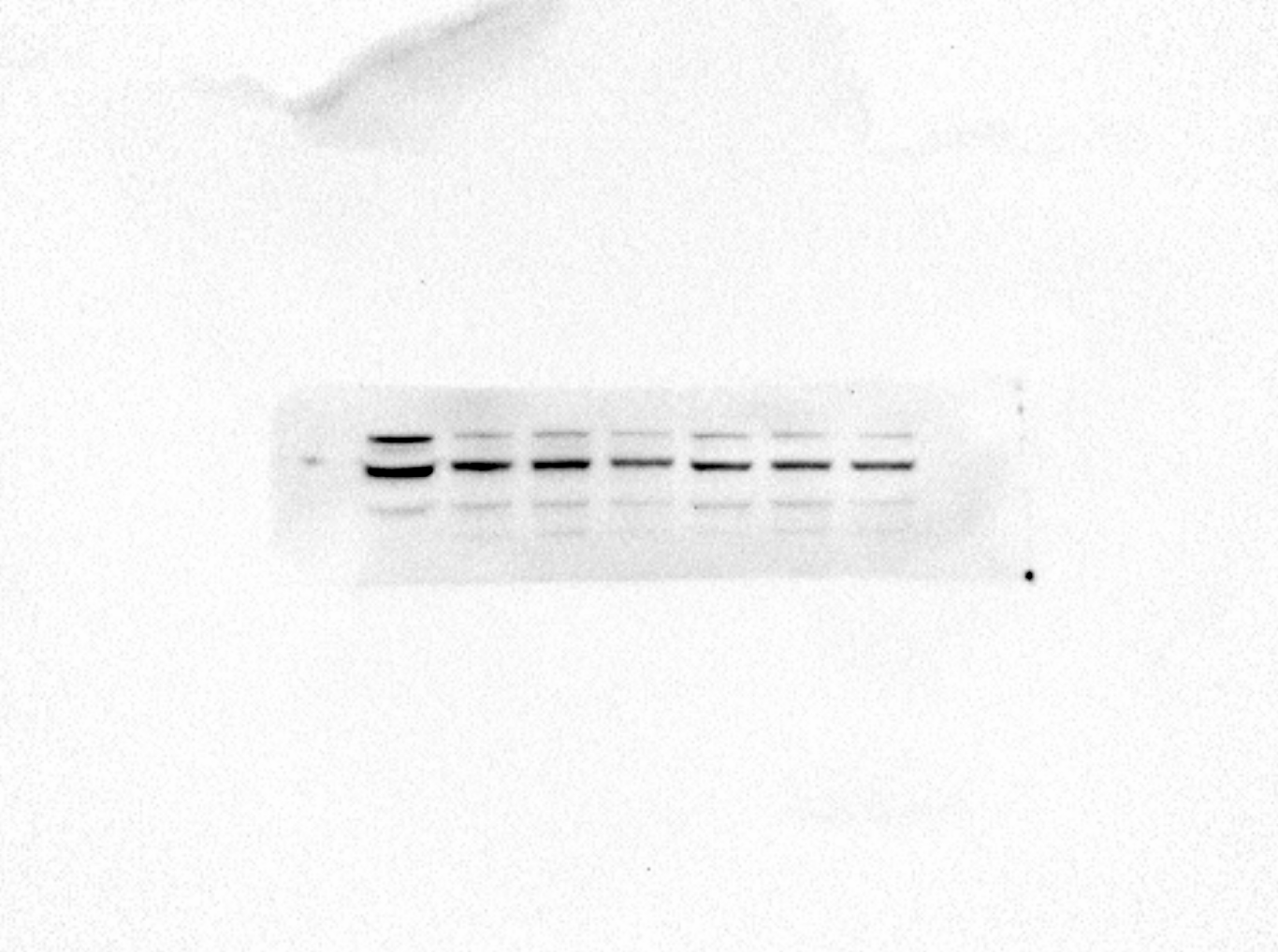
**(b)** GAPDH


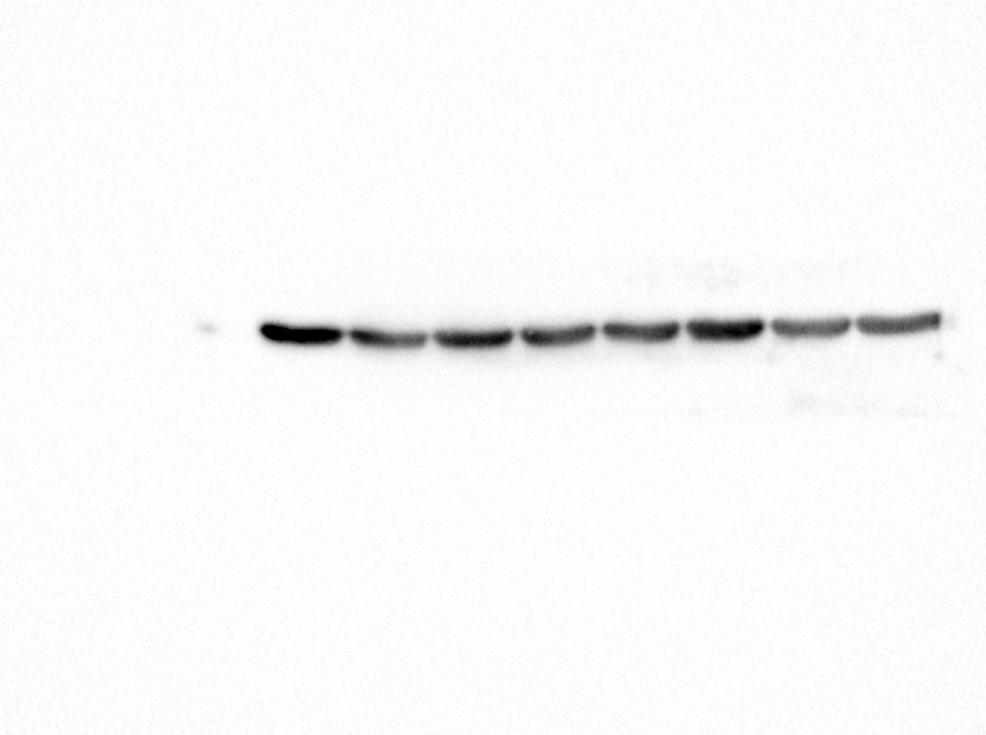

Supplement: Supplementary file 3 — Additional file 3. Original blot images of Fig. 3f. (a and b) Original blot images of Cap and GAPDH, respectively. [file 12866_2020_1986_MOESM3_ESM.docx]

**Original blot image of Fig. 6A**

**(a) p-PERK**


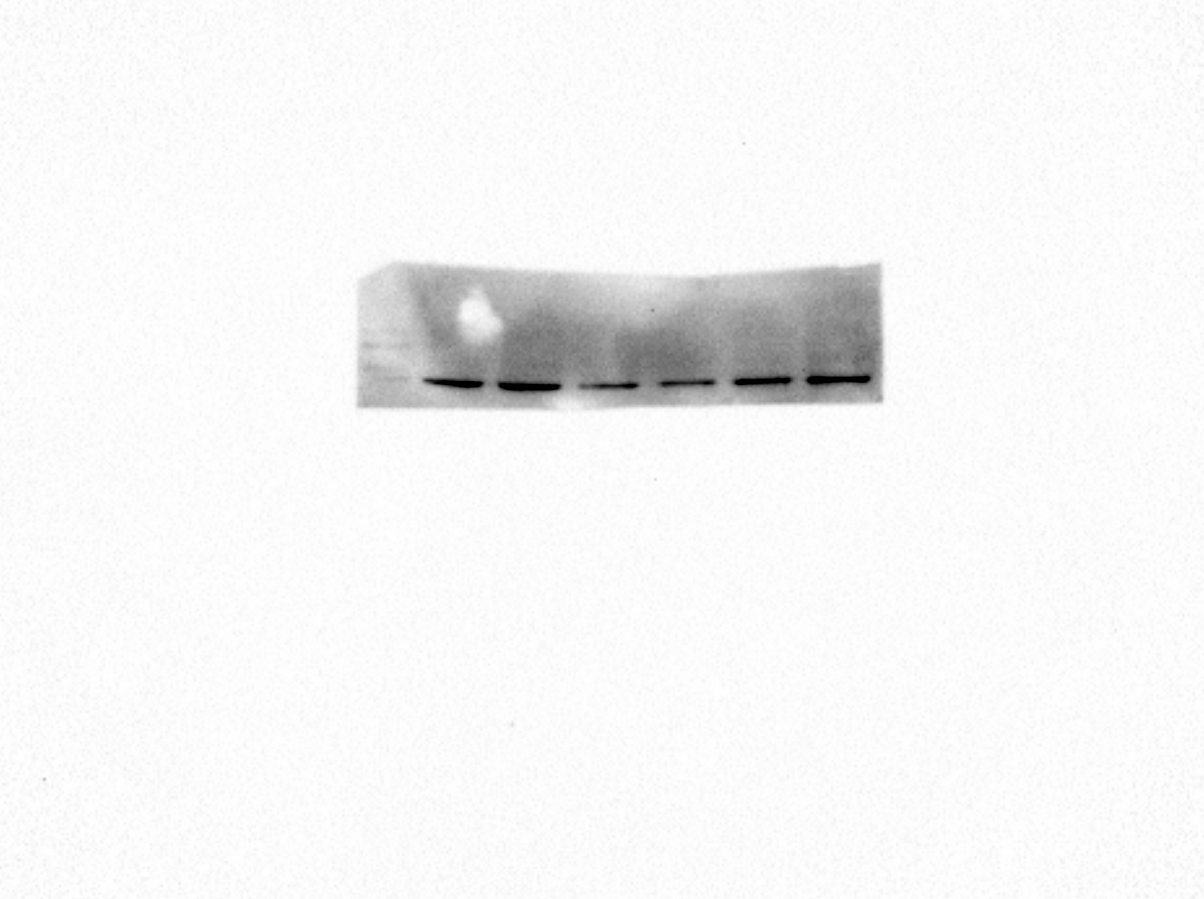


**(b) t-PERK**


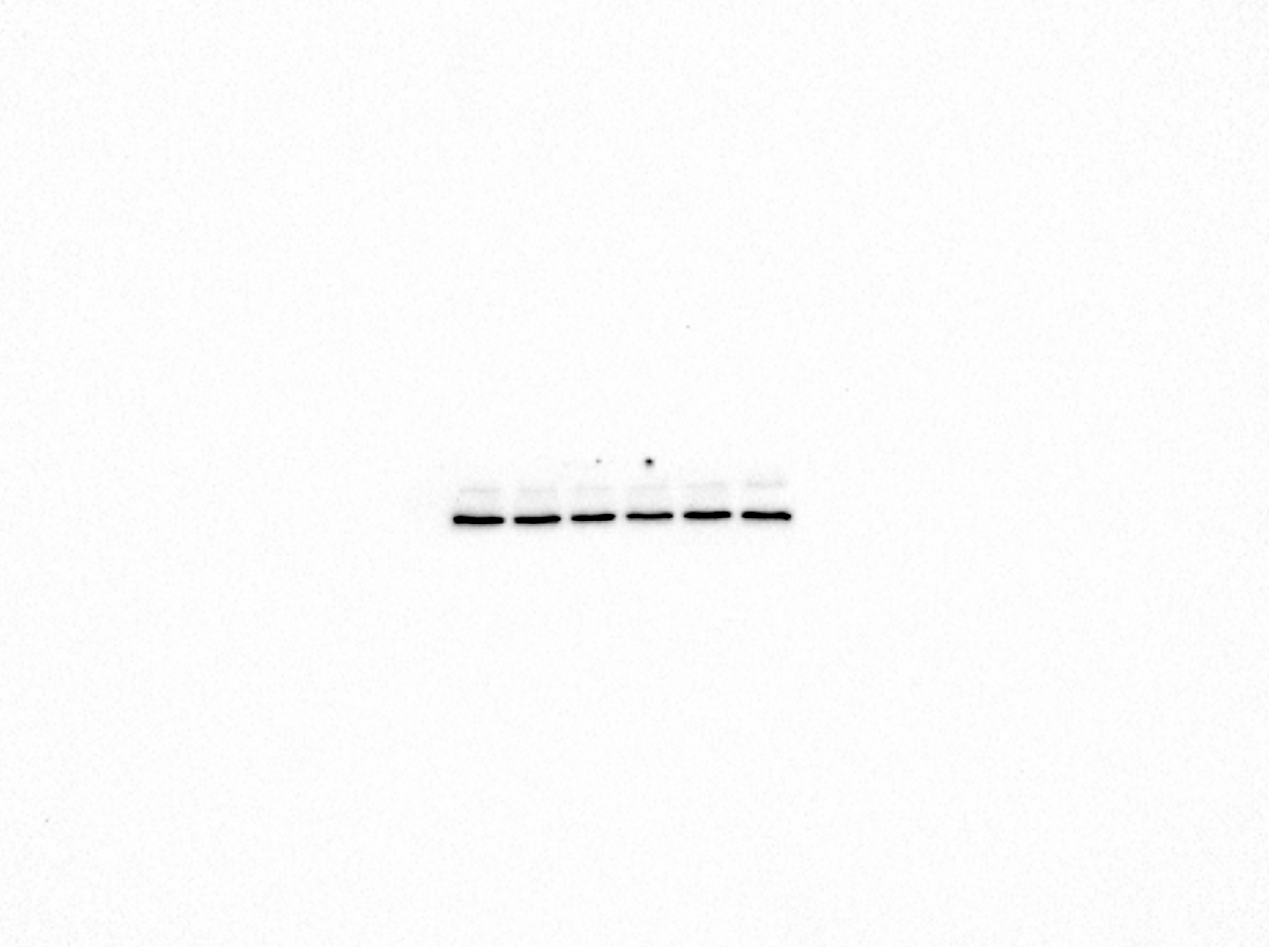


**(c) p-eIF2α**


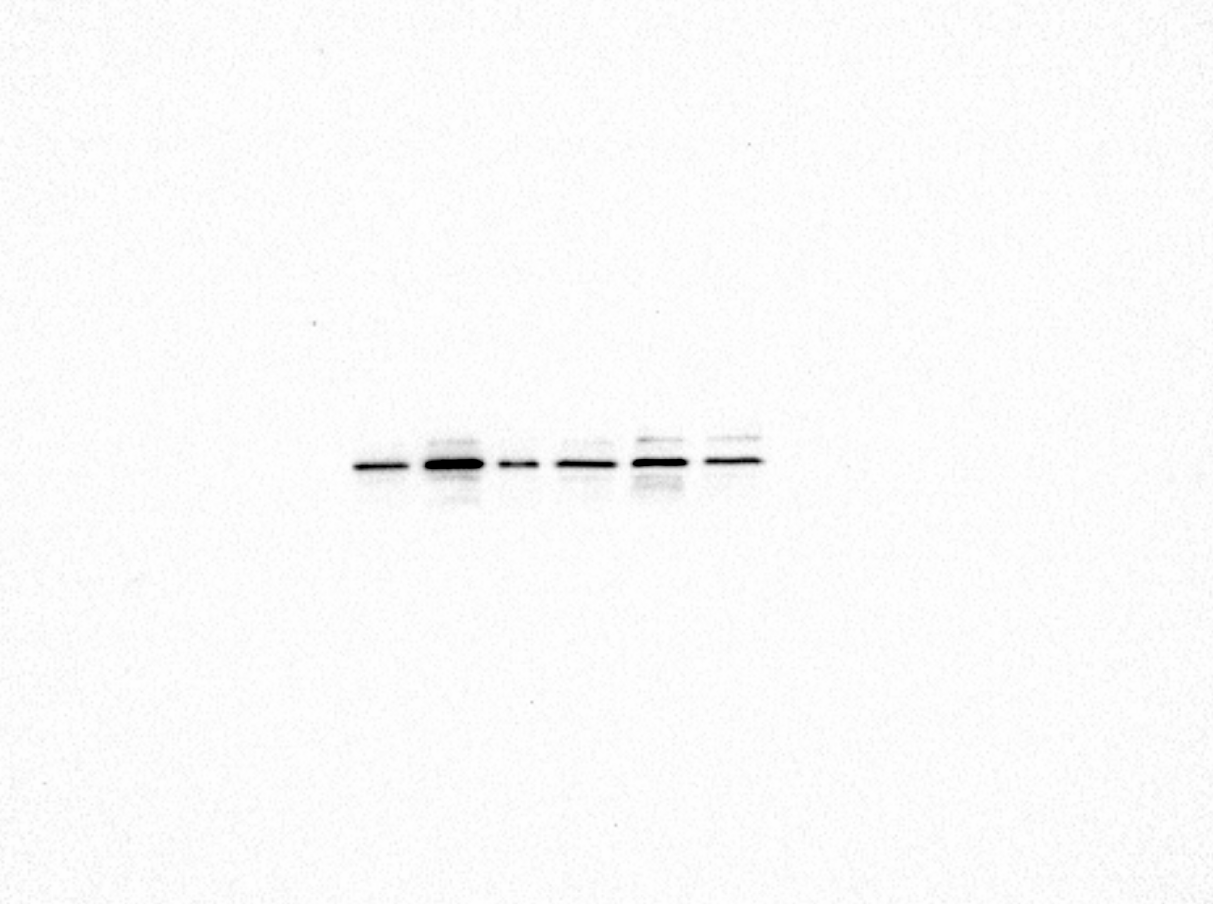


**(d) t-eIF2α**


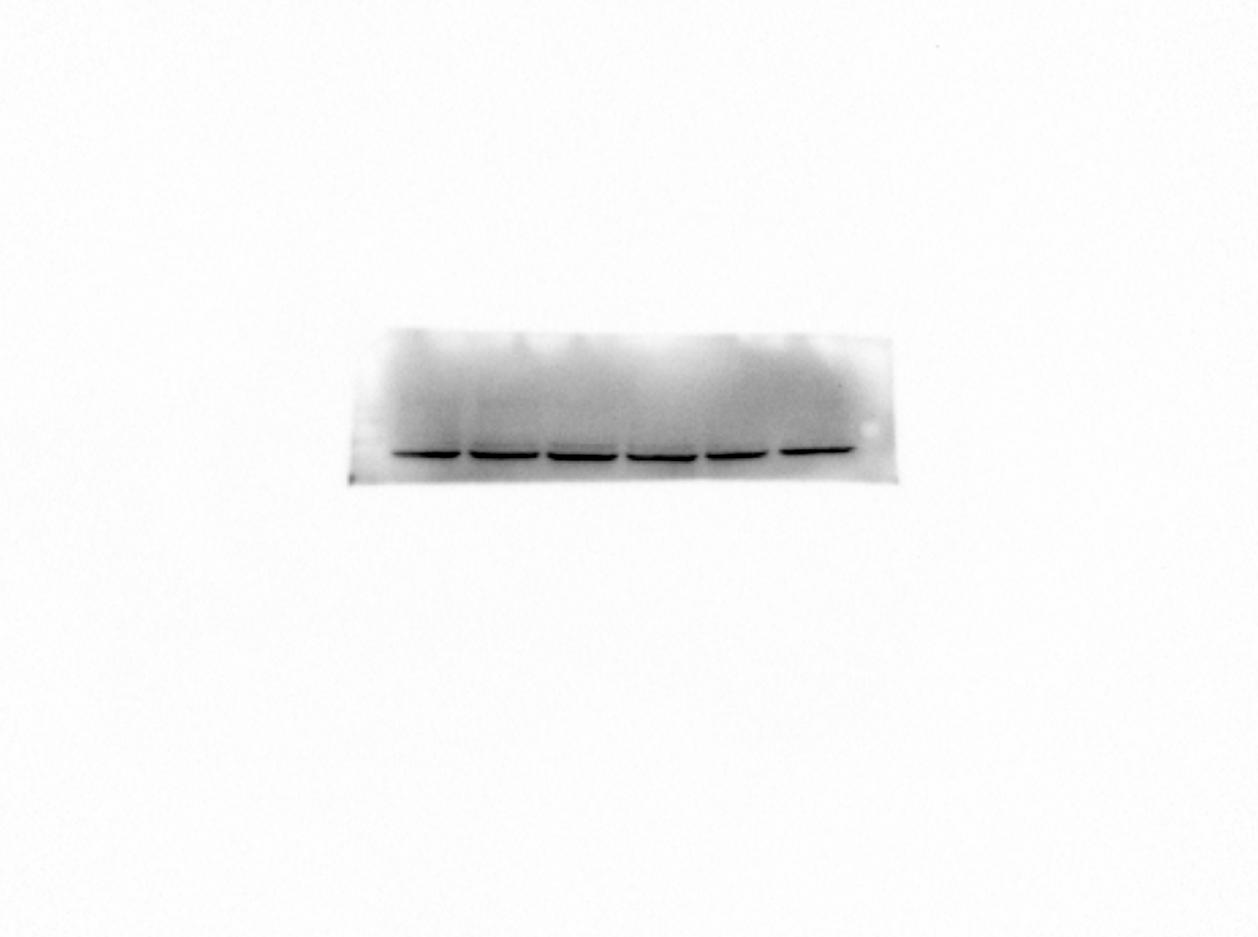


**(e) ATF4**


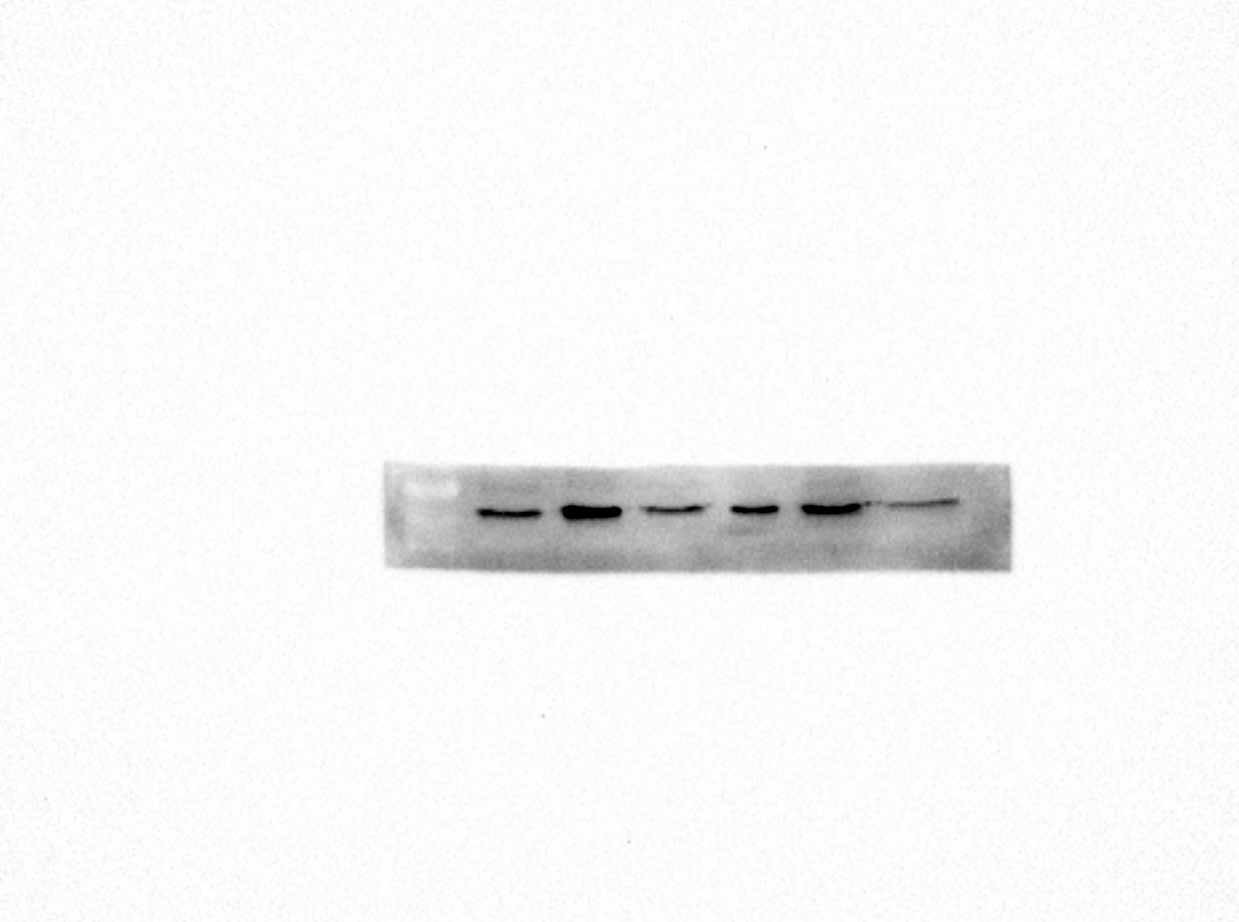


**(f) CHOP**


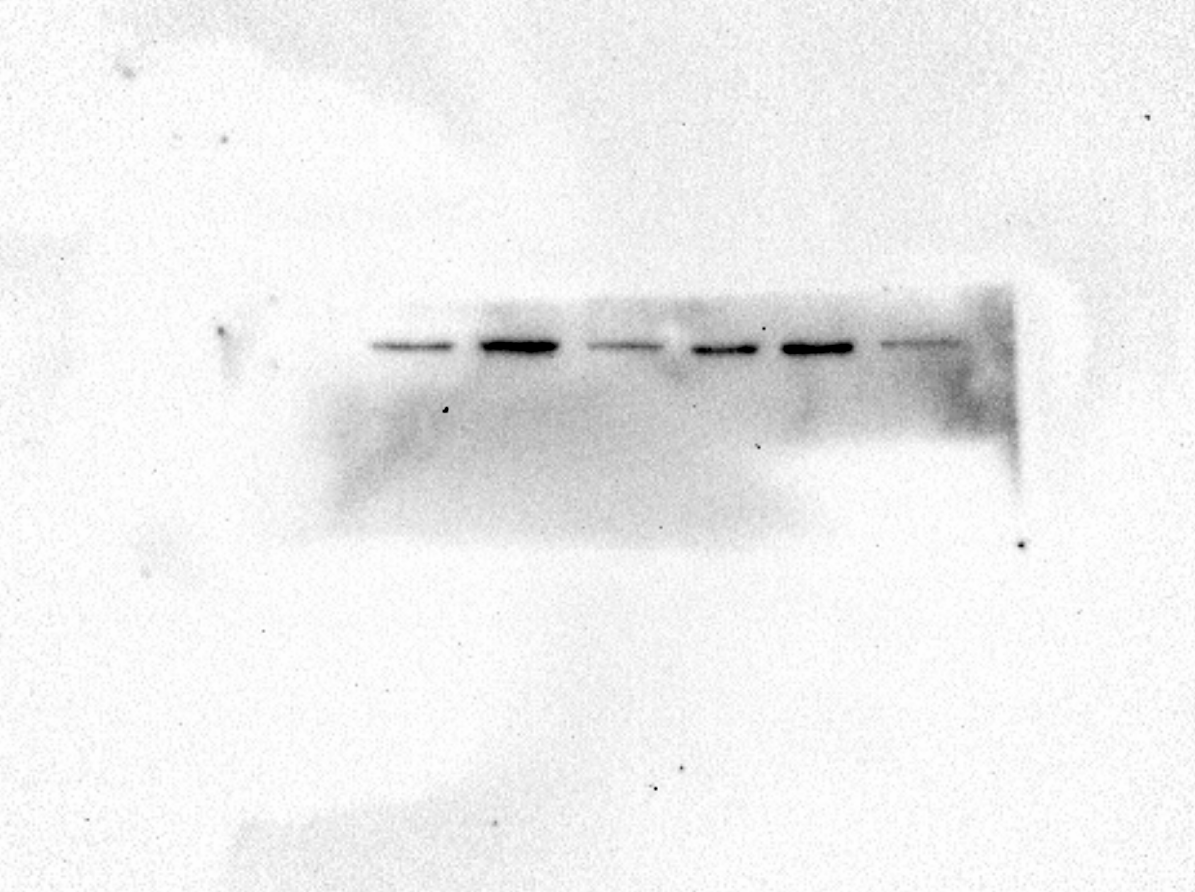


**(g) GAPDH**


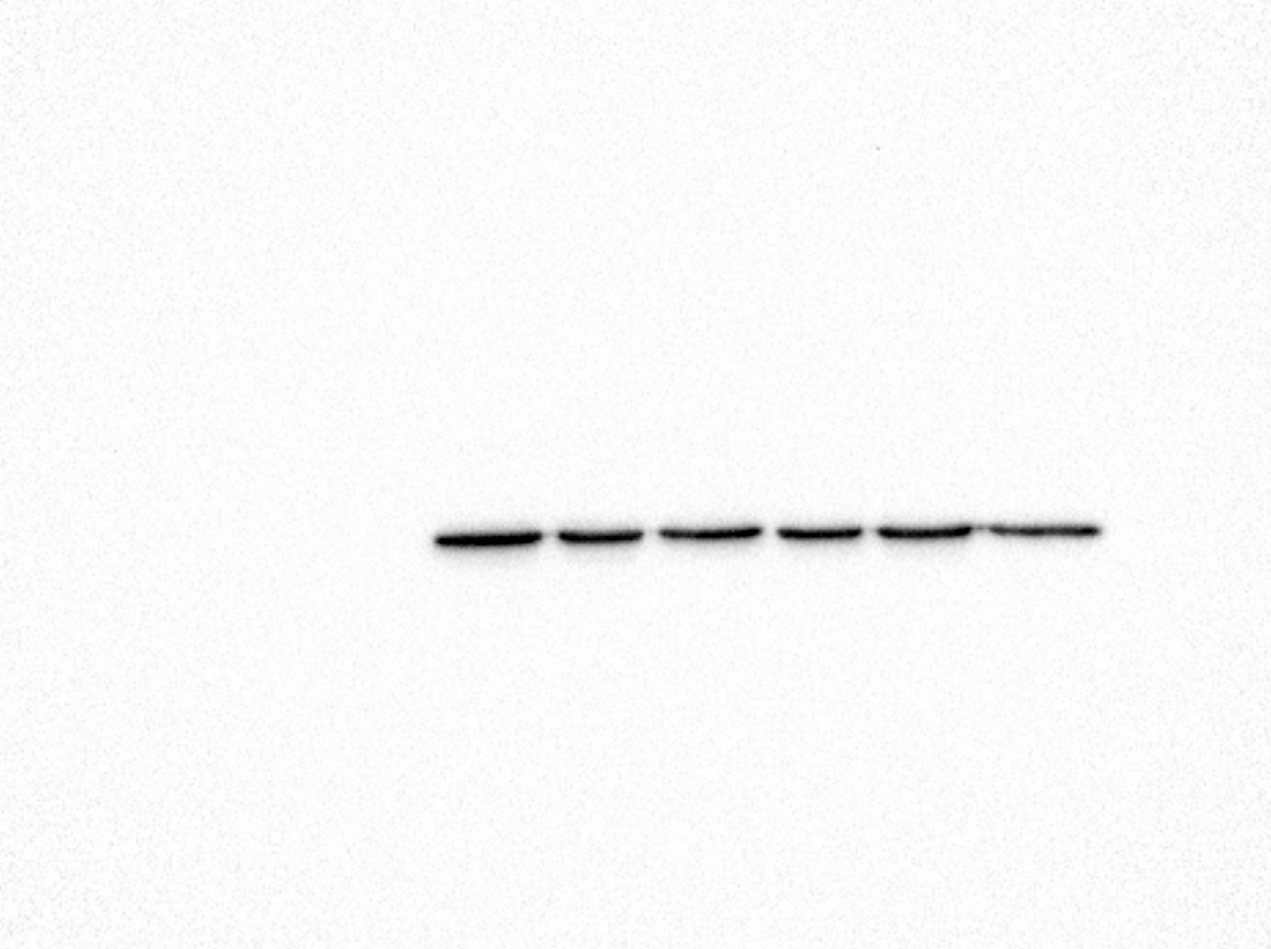

Supplement: Supplementary file 5 — Additional file 5. Original blot images of Fig. 5a. (a-g) Original blot images of p-PERK, t-PERK, p-eIF2α, t-eIF2α, ATF4, CHOP and GAPDH, respectively. [file 12866_2020_1986_MOESM5_ESM.docx]
