# Supplementary material for: The combined usage of Matrine and Osthole inhibited endoplasmic reticulum apoptosis induced by PCV2
Source: BMC Microbiol. 2020 Oct 12;20:303. doi: 10.1186/s12866-020-01986-2 (PMC7549248; doi:10.1186/s12866-020-01986-2)

**Original blot image of Fig. 5C and 5G**

**(a)** Cleaved-caspase 3 of Fig. 5C


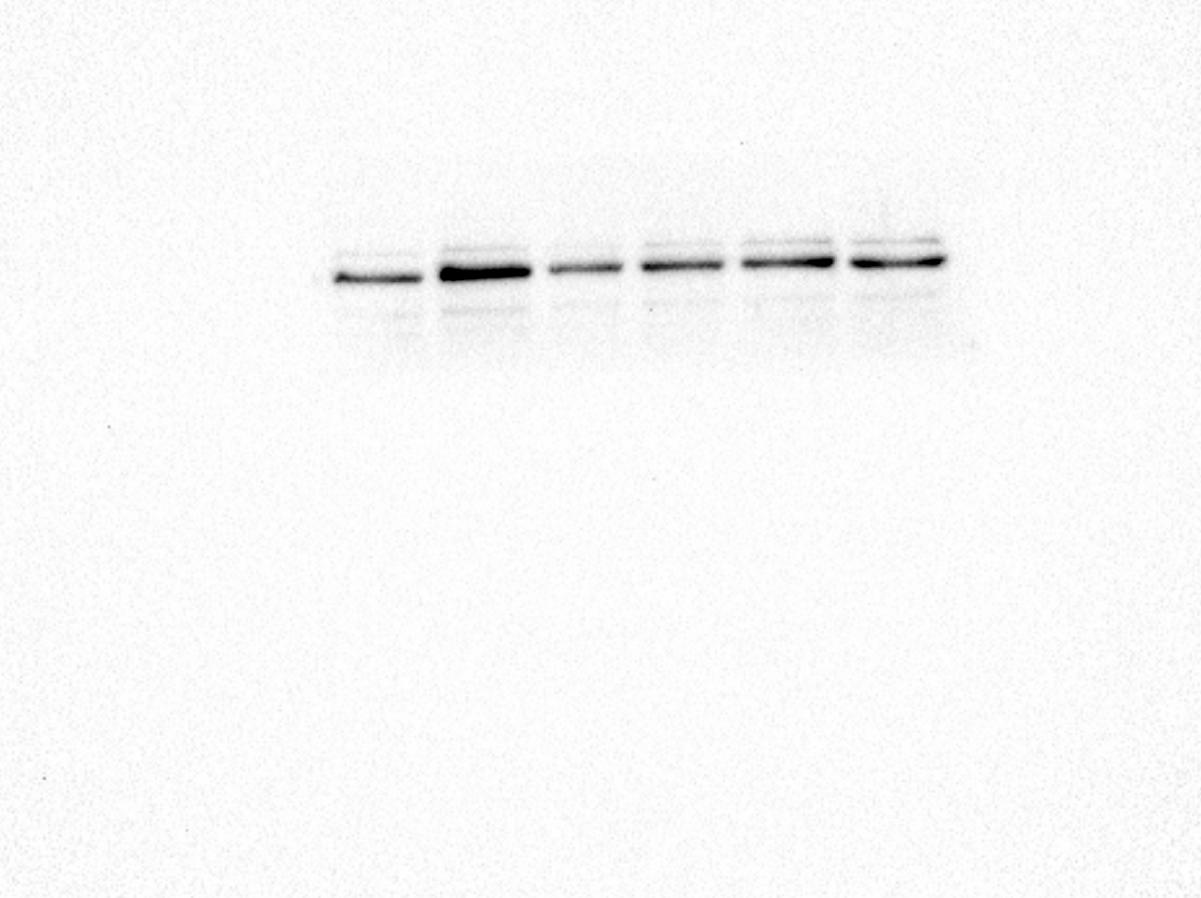


**(b)** GAPDH of Fig. 5C


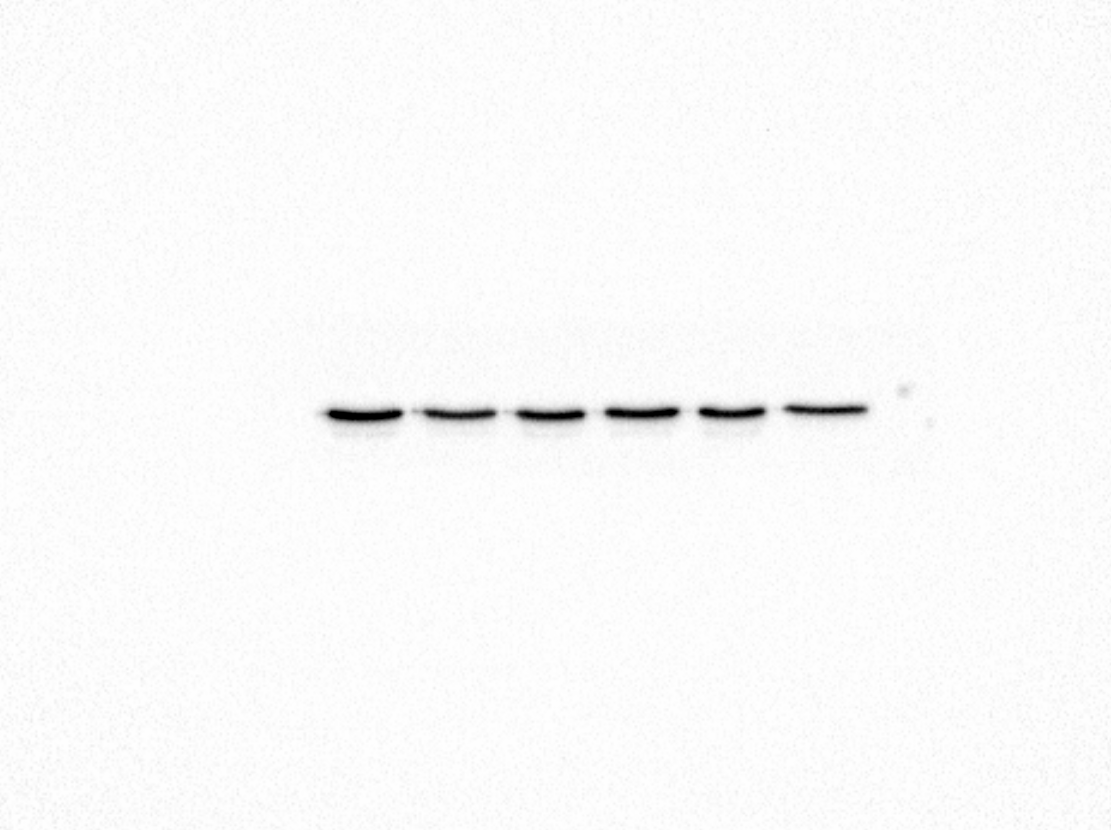


**(c)** Cleaved-caspase 9 of Fig. 5G


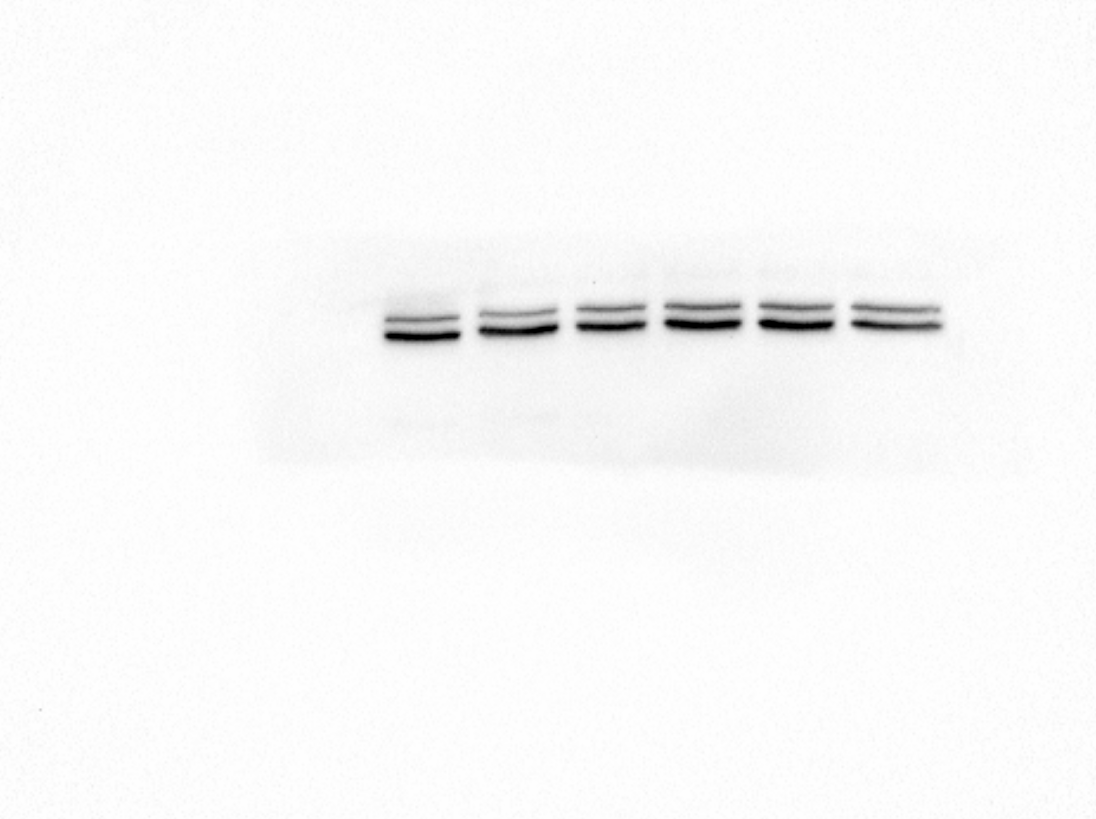


**(d)** Bcl-2 of Fig. 5G


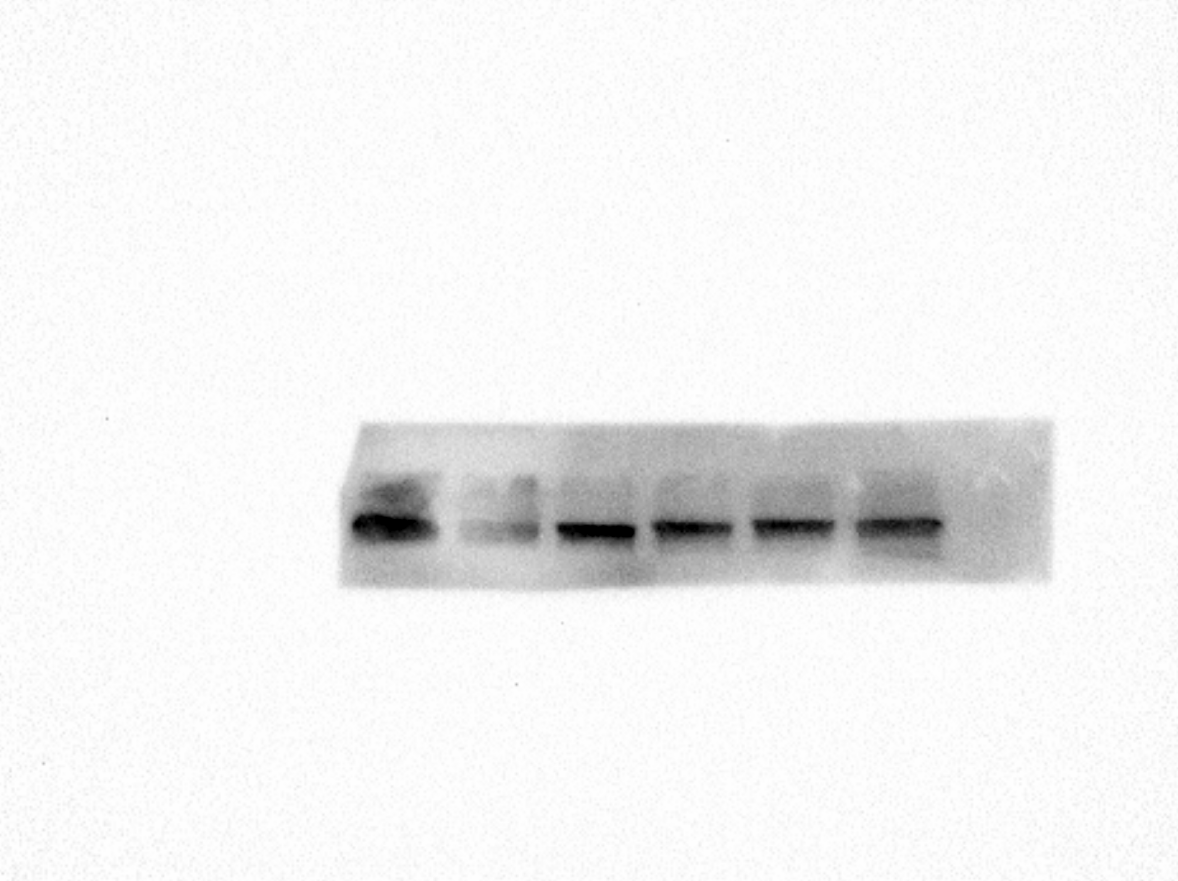


**(e)** Bax of Fig. 5G


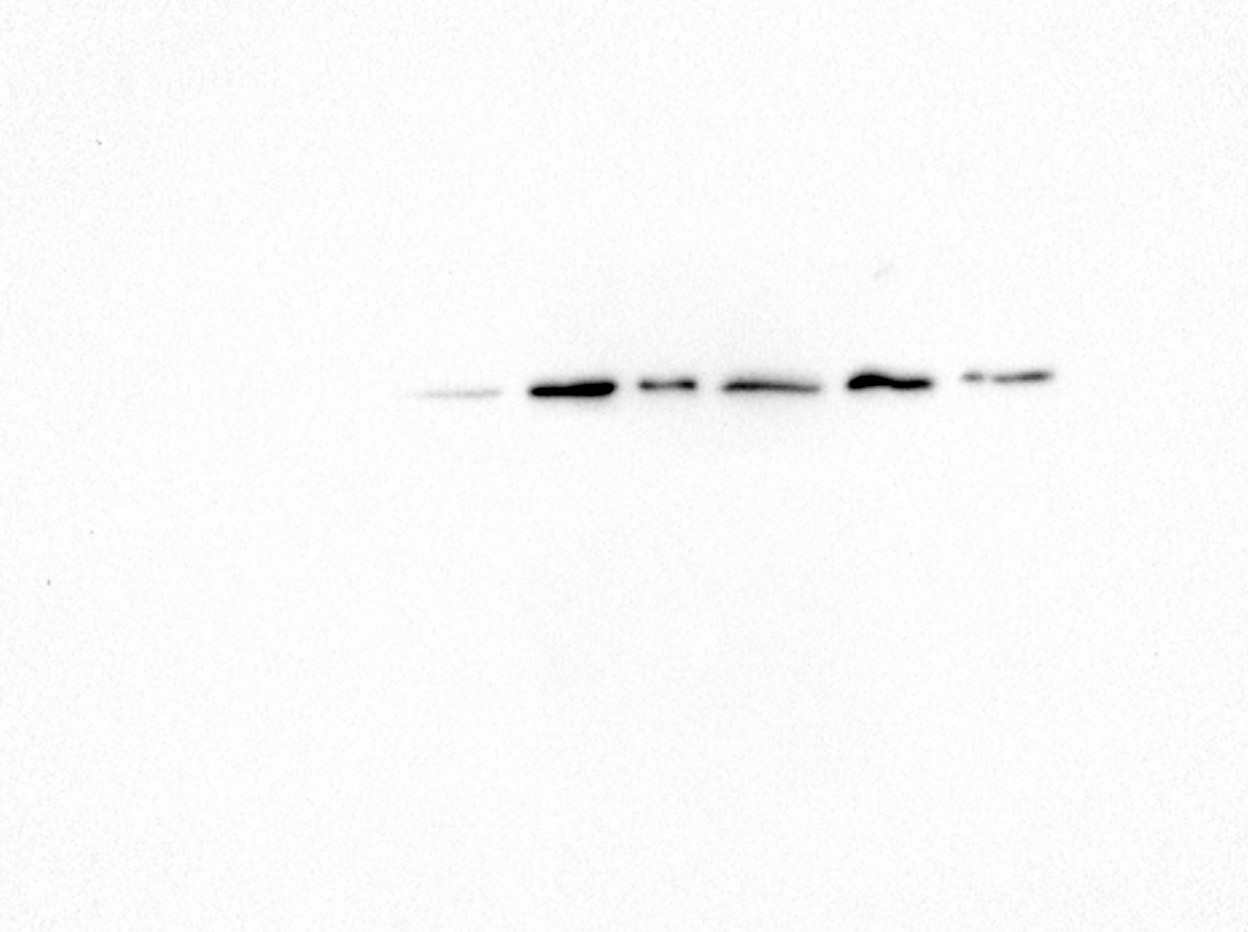


**(f)** GRP78 of Fig. 5G


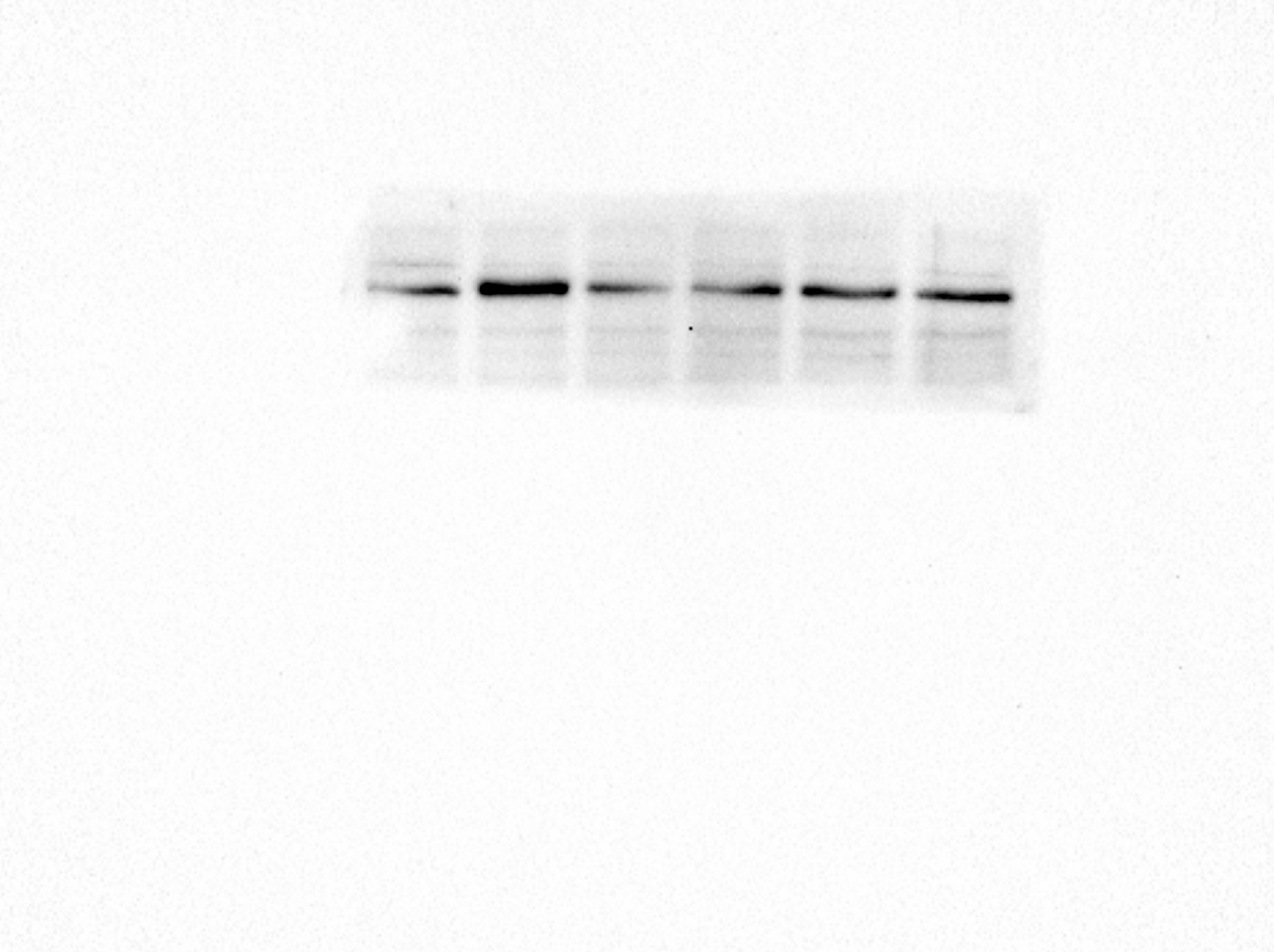


**(g)** GAPDH of Fig. 5G


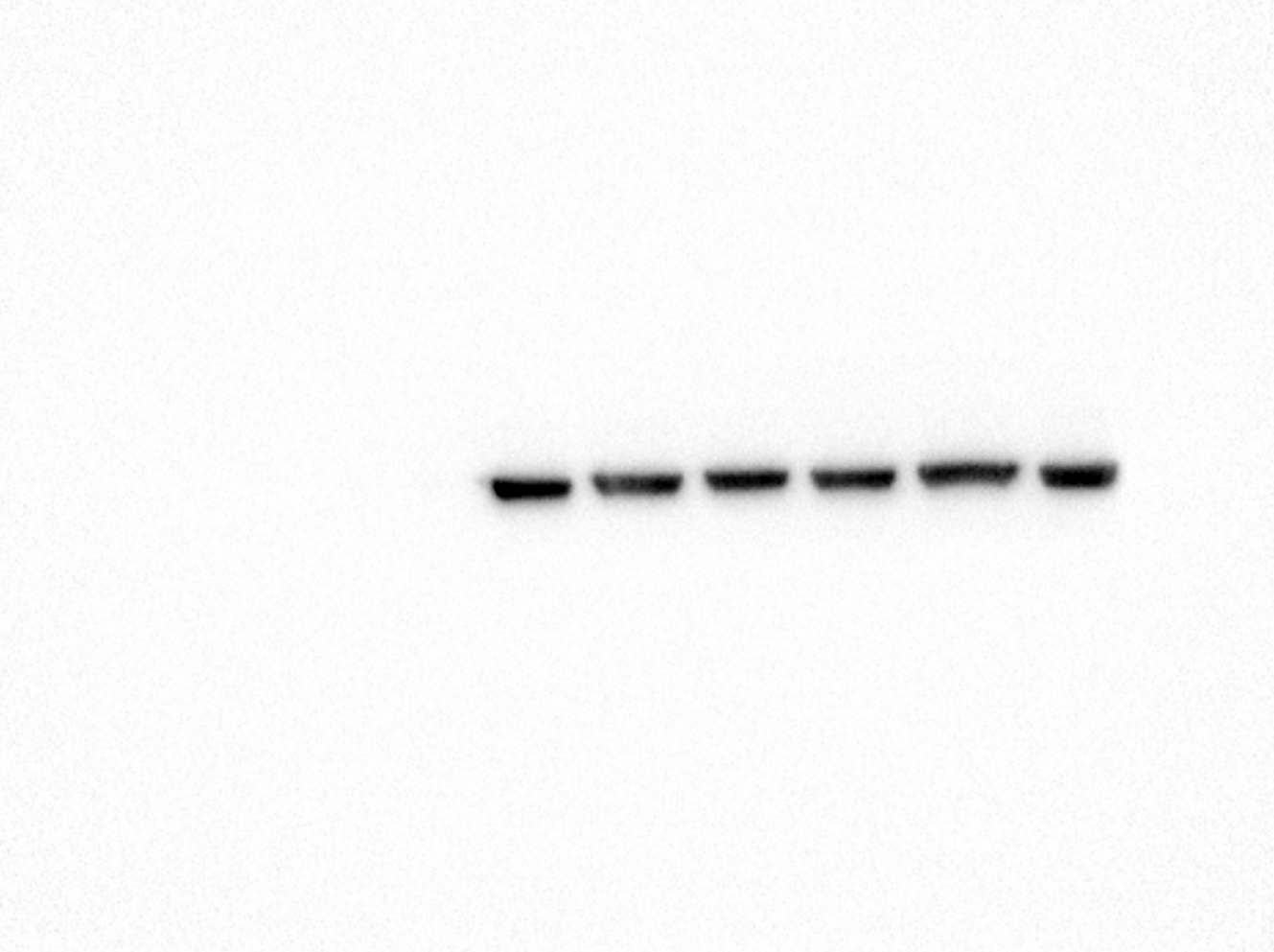

Supplement: Supplementary file 4 — Additional file 4. Original blot images of Fig. 4c and g. (a and b) Original blot images of cleaved caspase-3 and GAPDH in the Fig. 4c, respectively. (c and g) Original blot images of cleaved caspase-3 and GAPDH, respectively. (c-g) Original blot images of cleaved caspase-9, Bcl-2, Bax, GRP78, and GAPDH in the Fig. 4g, respectively. [file 12866_2020_1986_MOESM4_ESM.docx]
