# Supplementary material for: The combined usage of Matrine and Osthole inhibited endoplasmic reticulum apoptosis induced by PCV2
Source: BMC Microbiol. 2020 Oct 12;20:303. doi: 10.1186/s12866-020-01986-2 (PMC7549248; doi:10.1186/s12866-020-01986-2)

**Original blot image of Fig. 7A, 7C and 7H**

**(a**) Cap of Fig. 7A


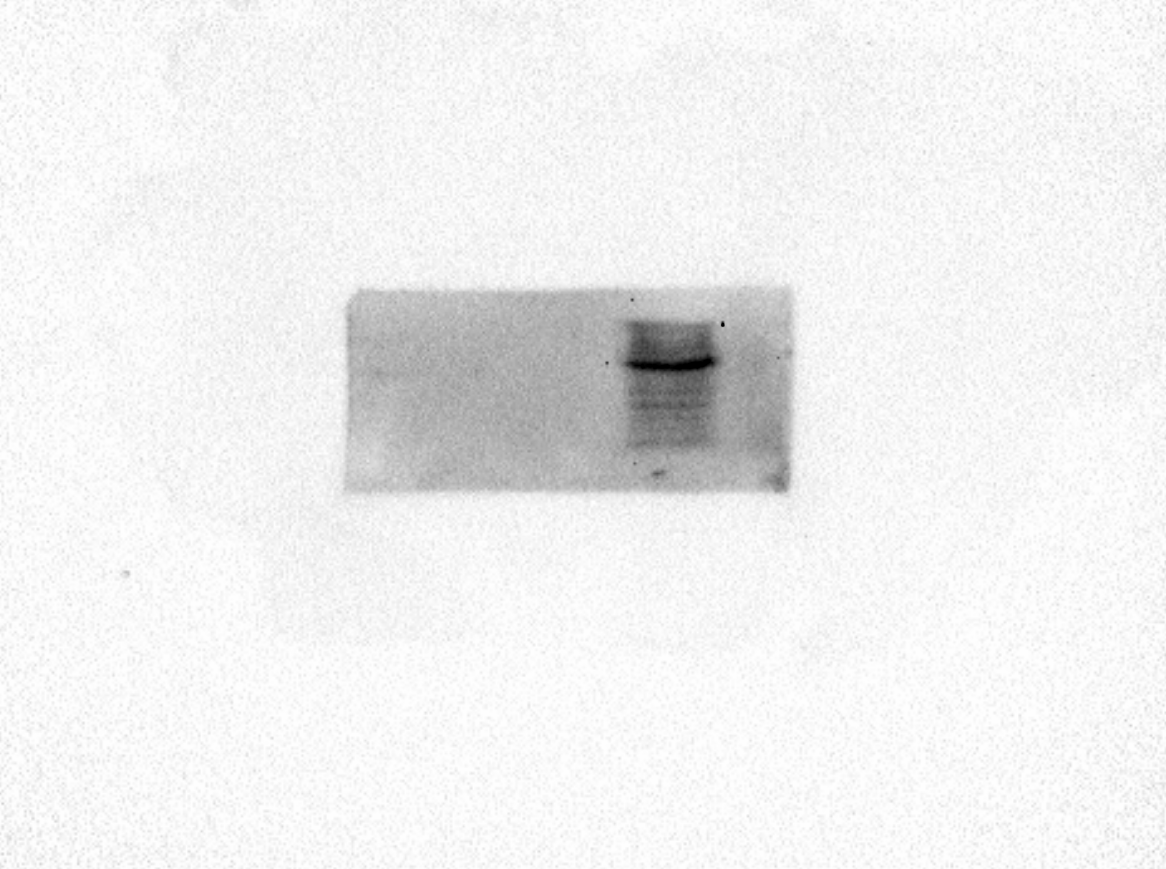


**(b)** GAPDH of Fig. 7A


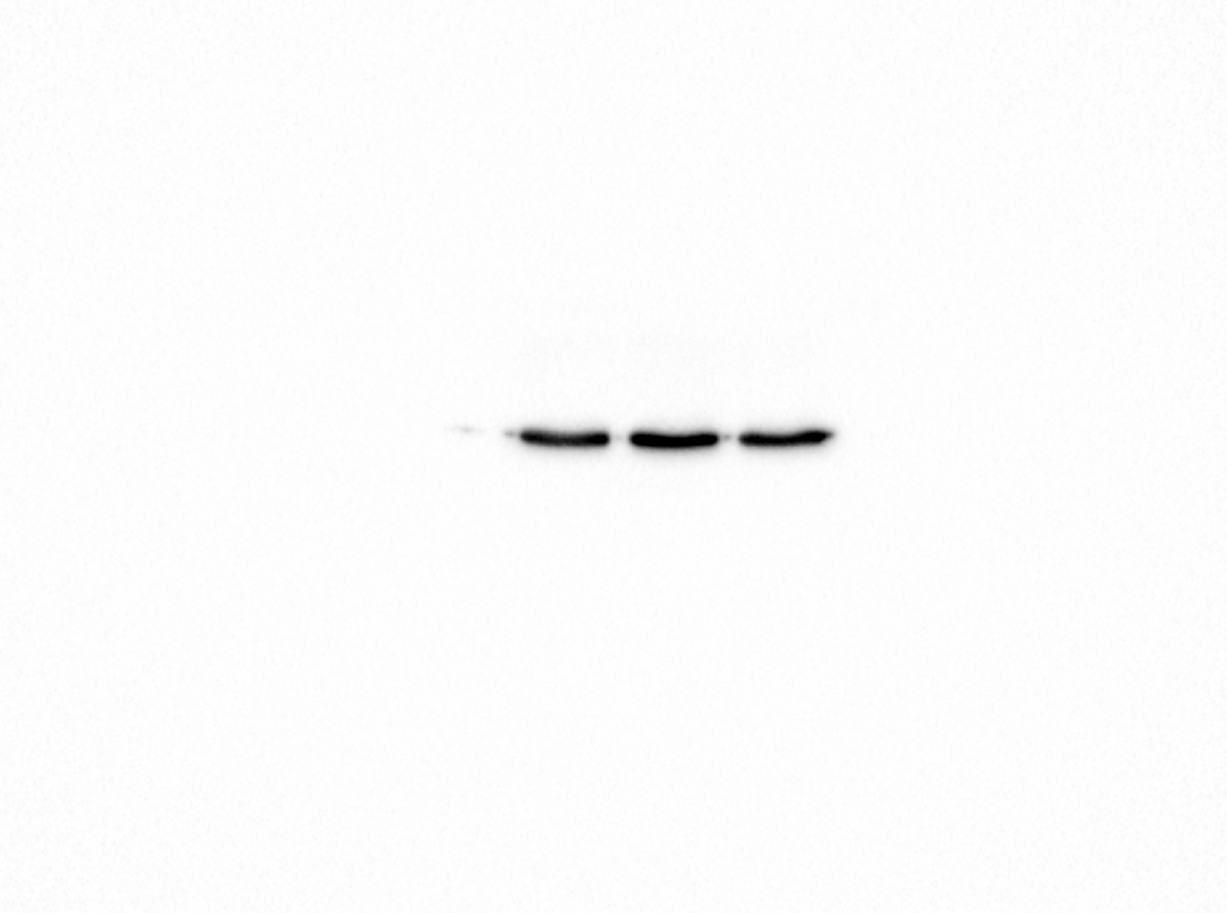


**(c)** Cap of Fig. 7C


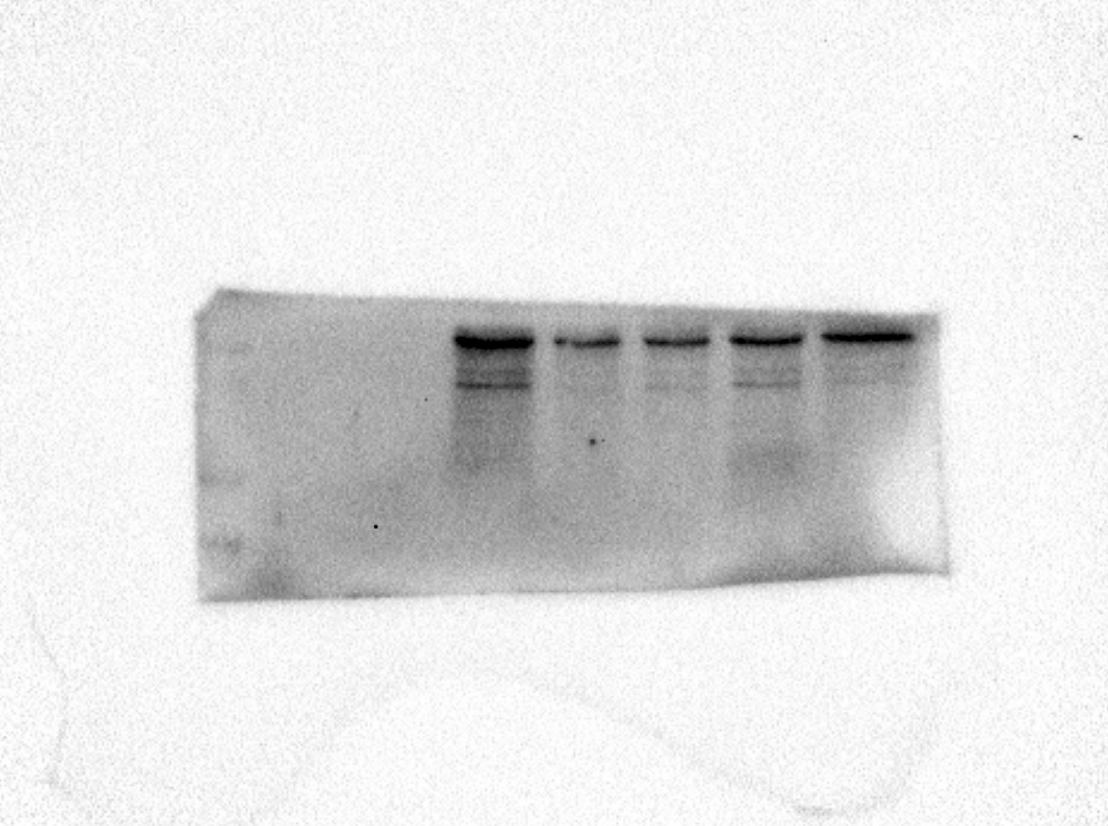


**(d)** GRP78 of Fig. 7C


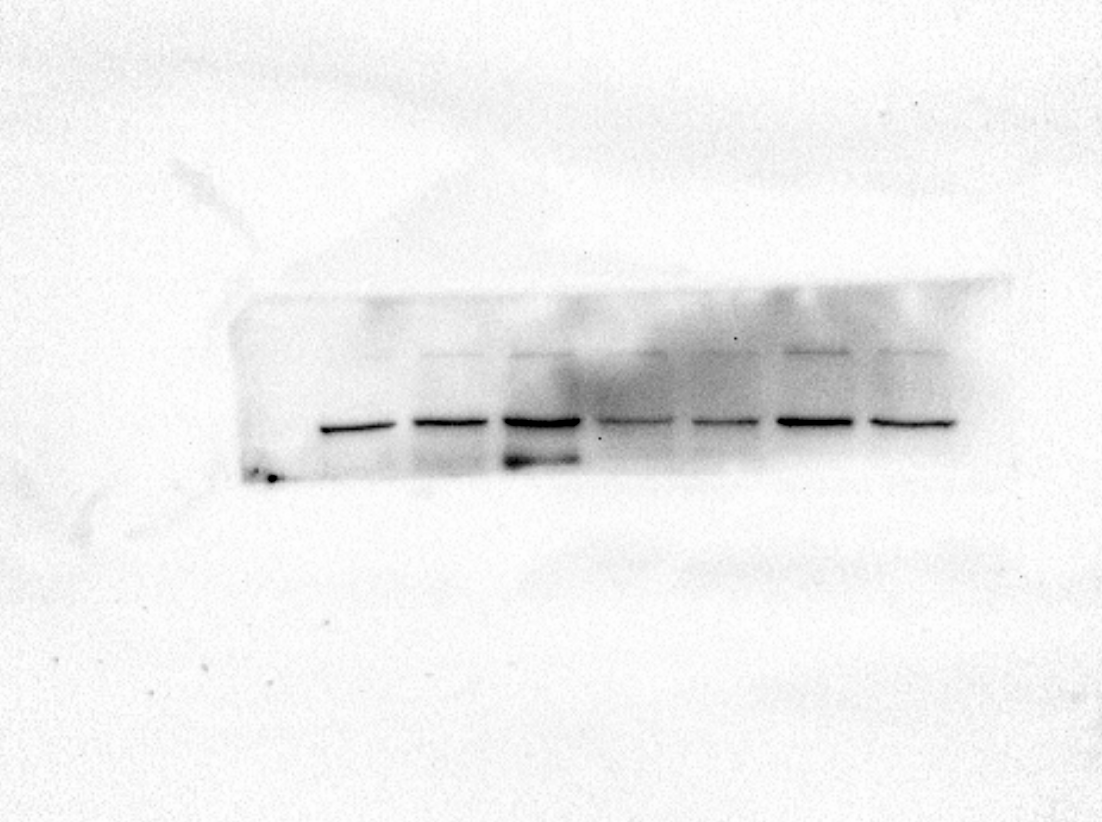


**(e)** Cleaved-caspase 3 of Fig. 7C


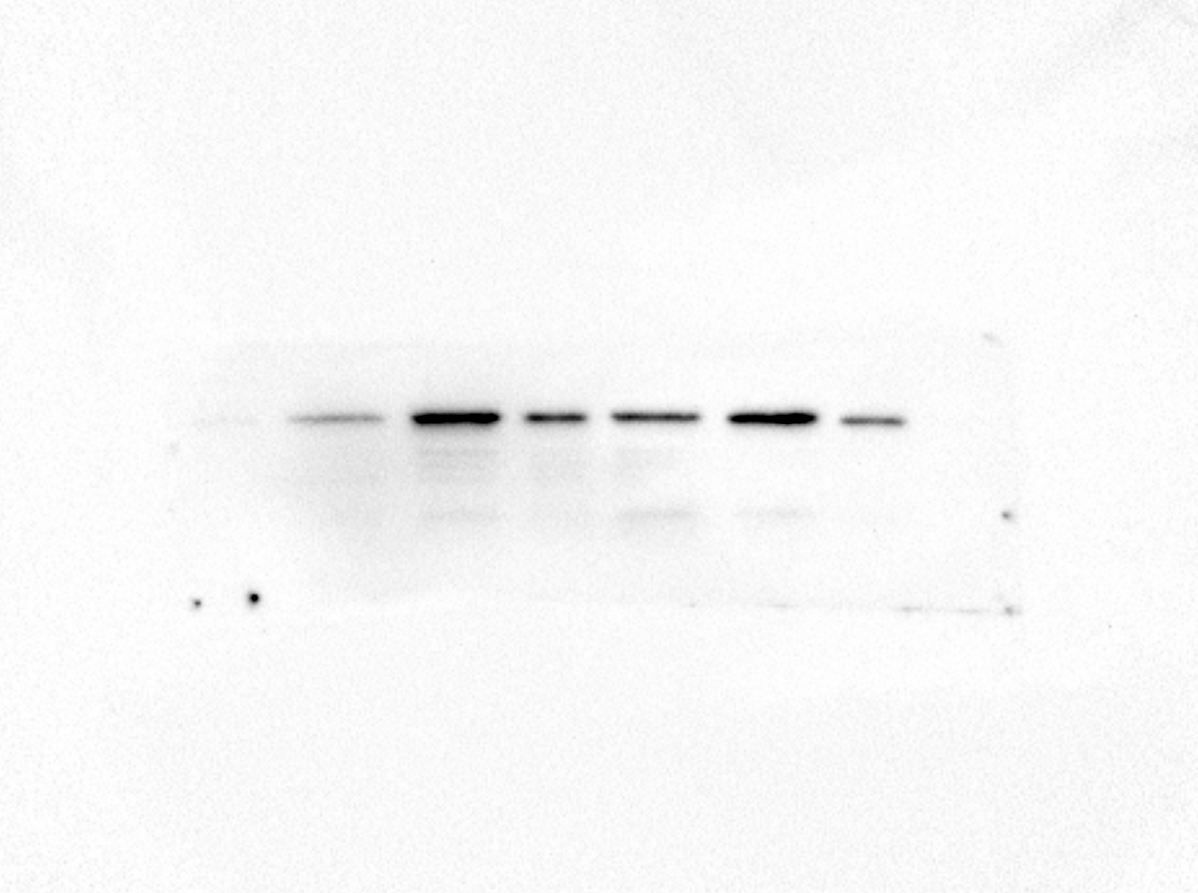


**(f)** Bcl-2 of Fig. 7C


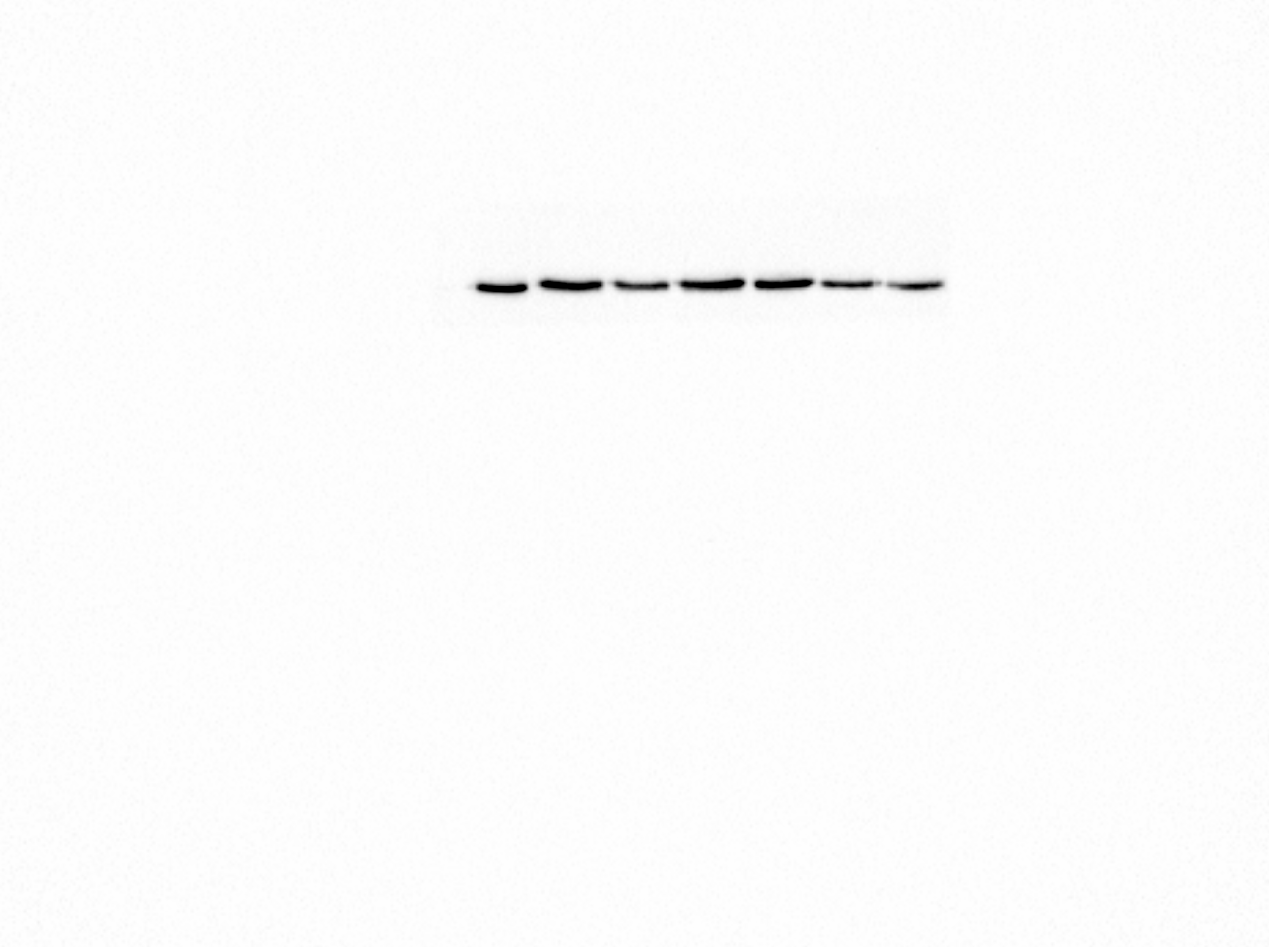


**(g)** GAPDH of Fig. 7C


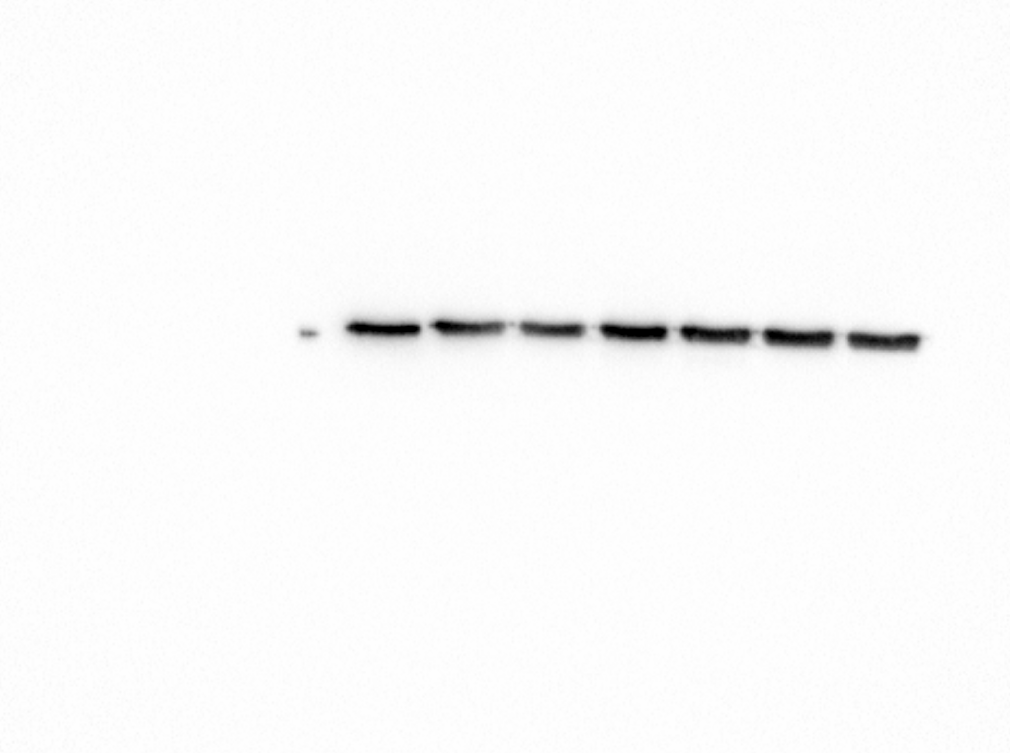


**(h)** p-PERK of Fig. 7H


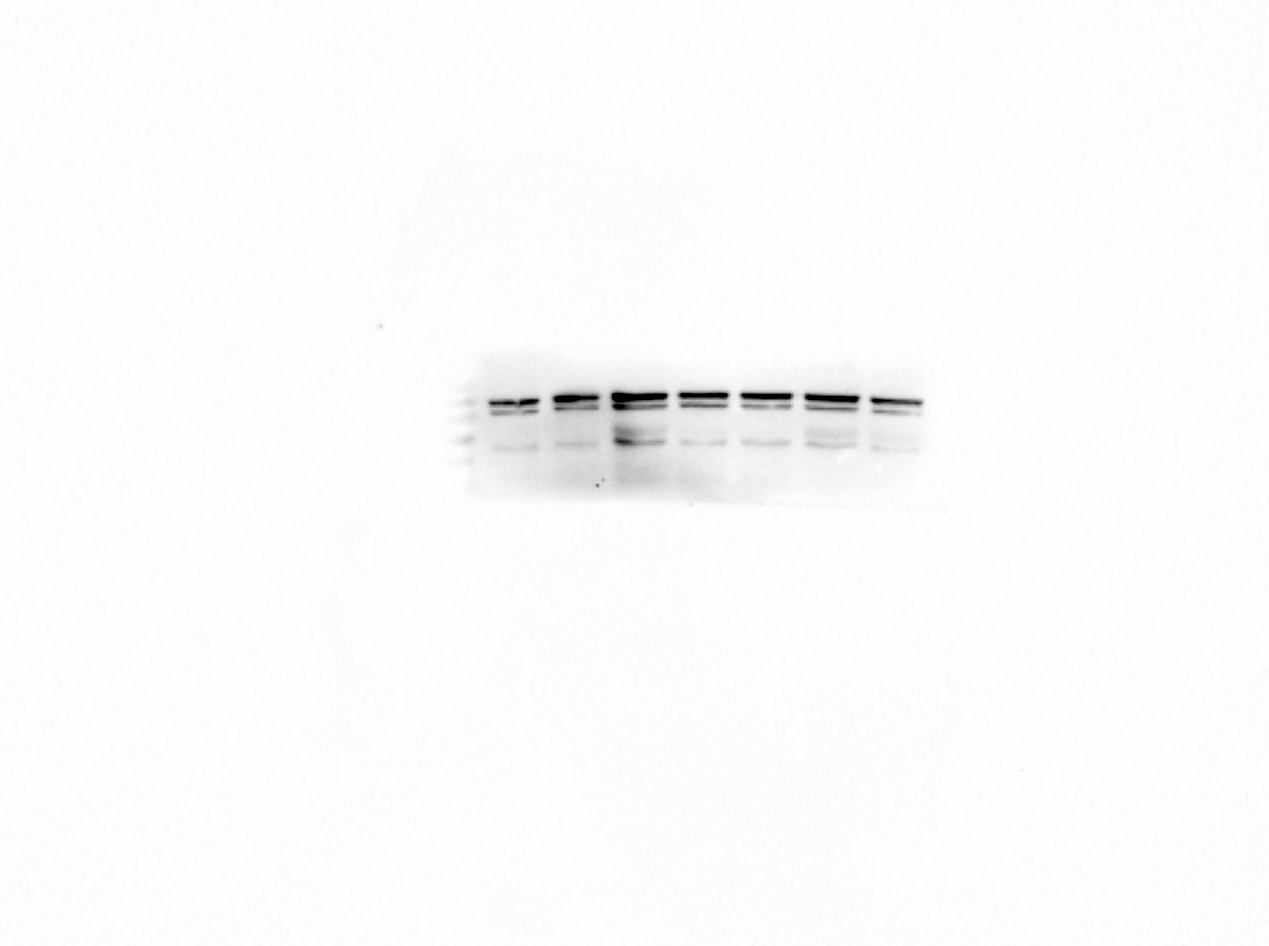


**(i)** t-PERK of Fig. 7H


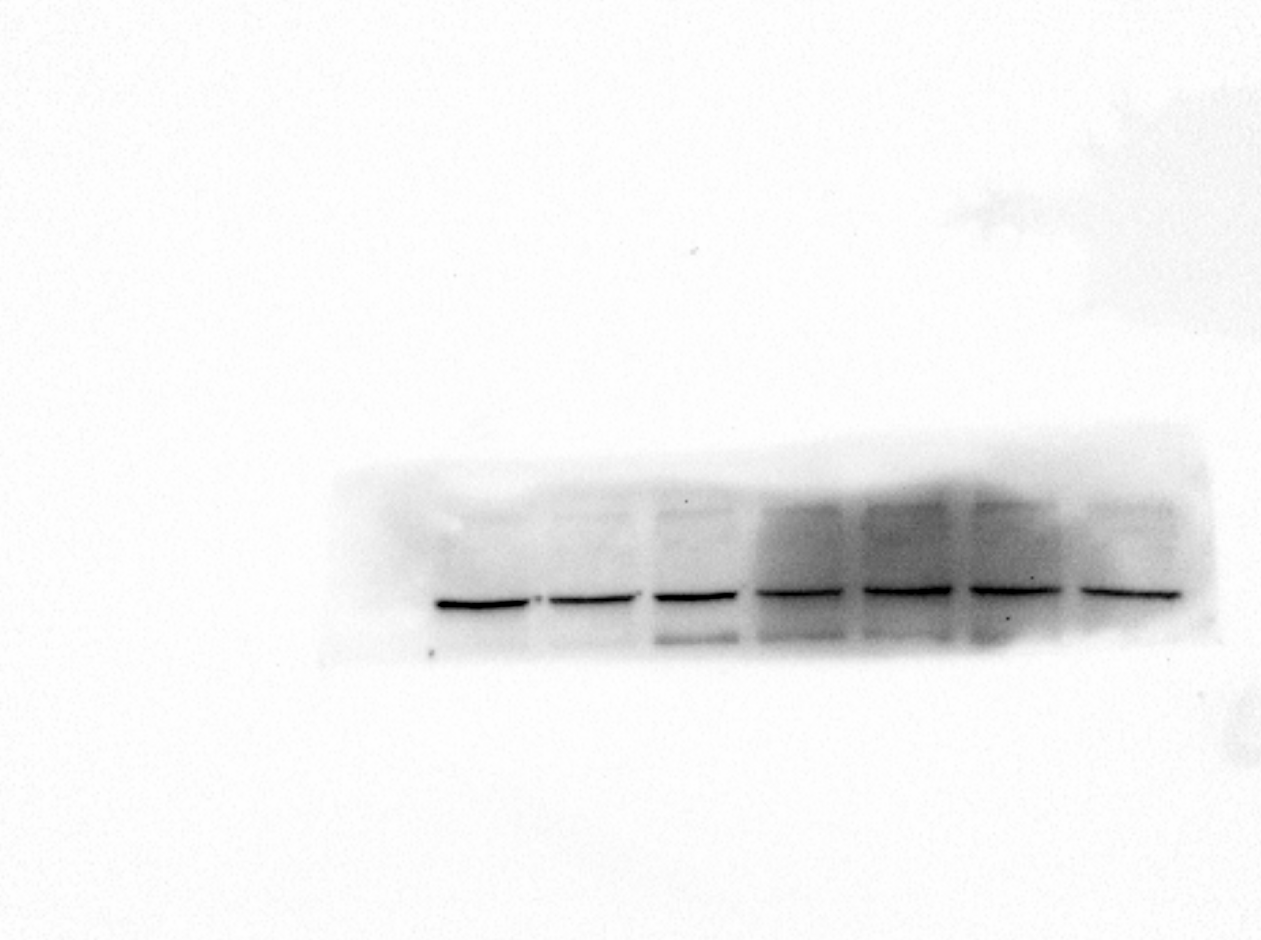


**(j)** p-eIF2α of Fig. 7H


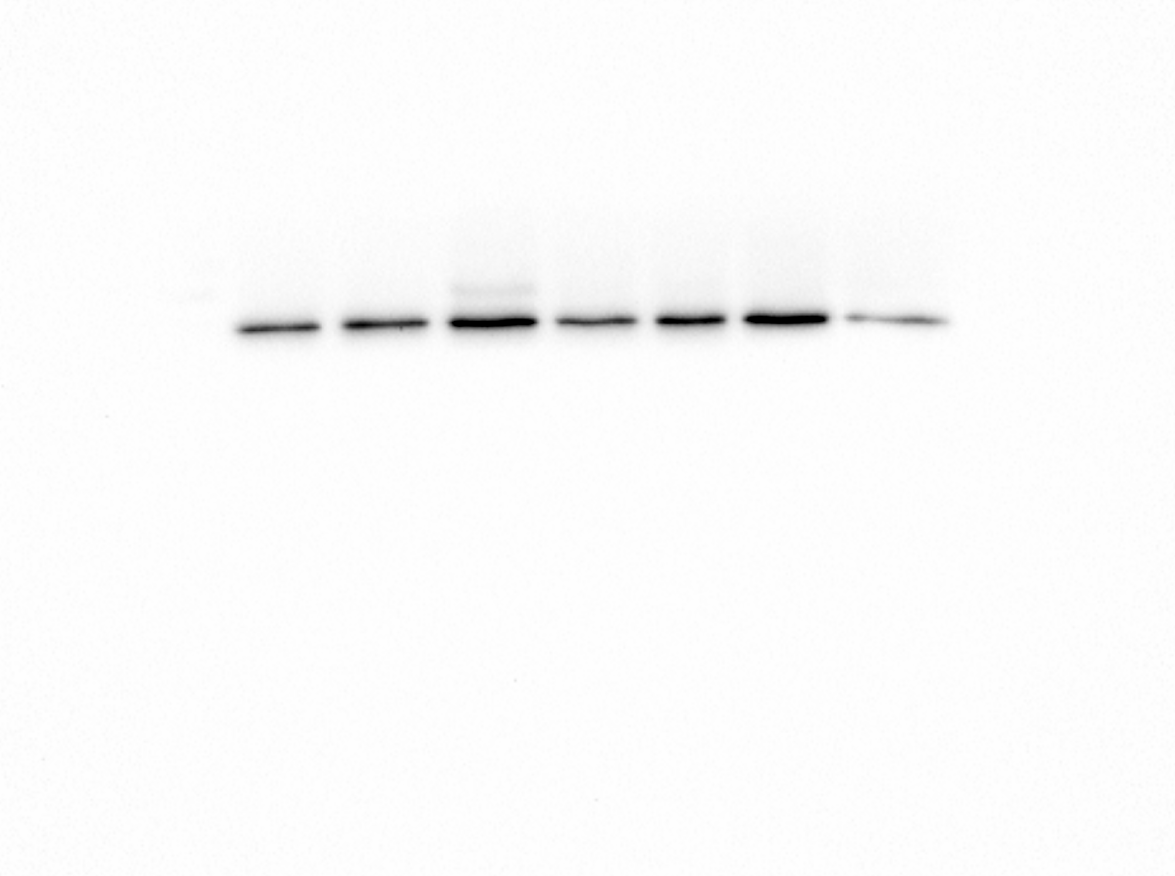


**(k)** t-eIF2α of Fig. 7H


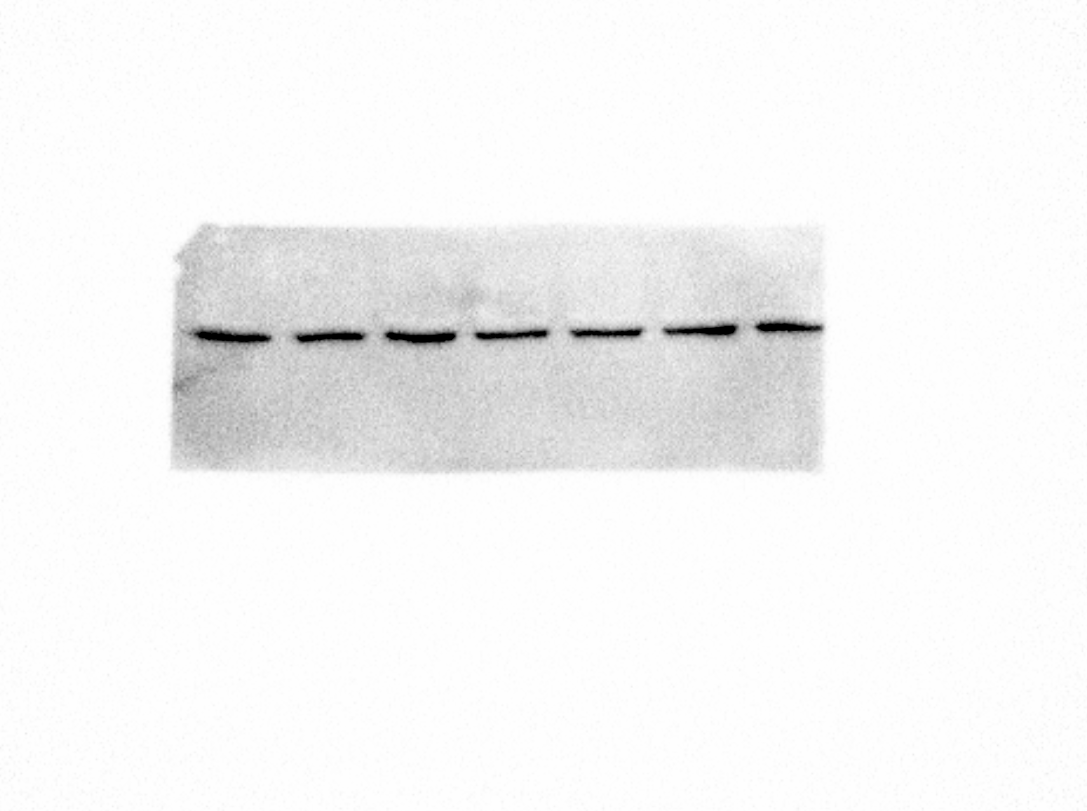


**(l)** ATF4 of Fig. 7H


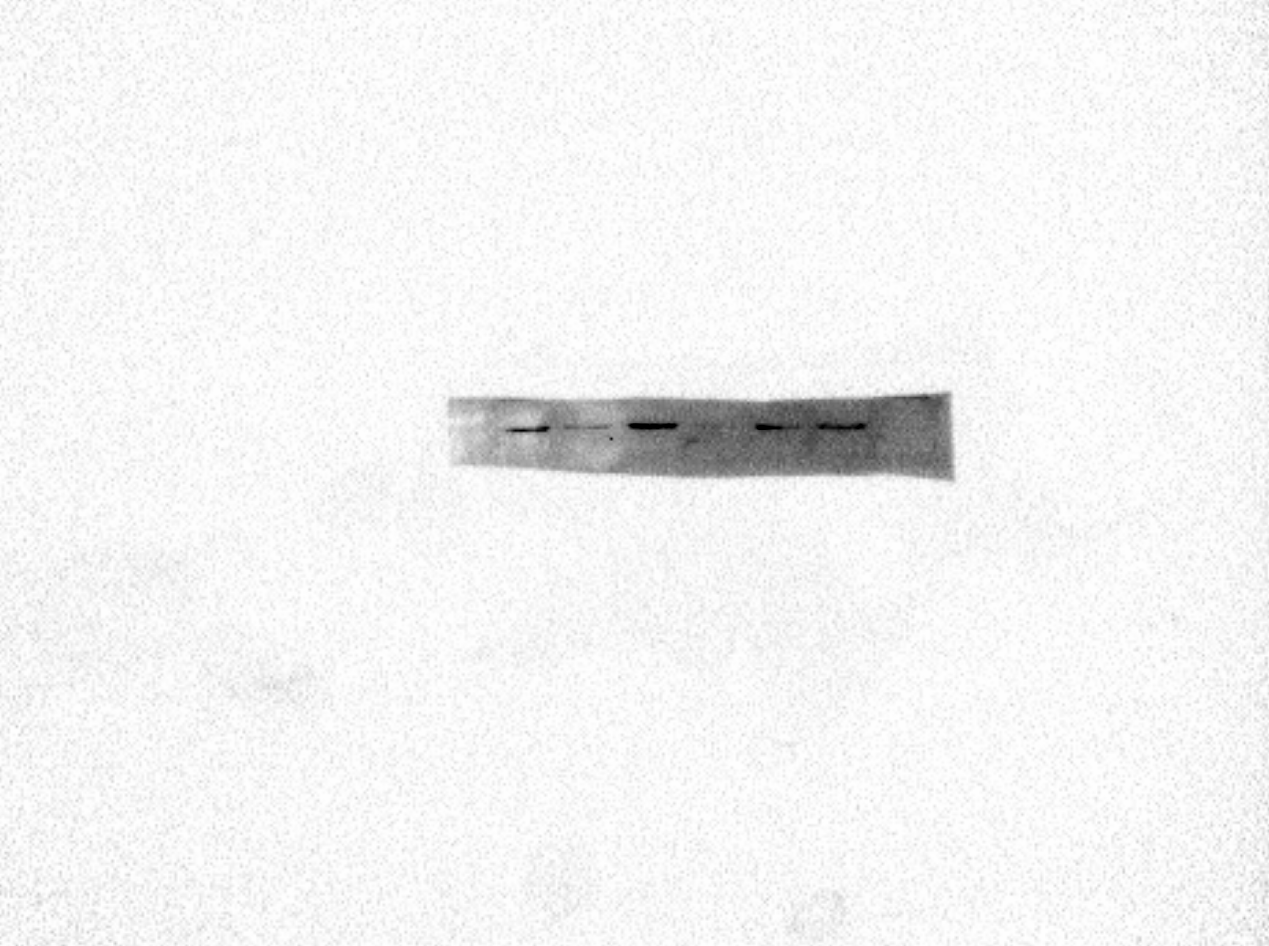


**(m)** CHOP of Fig. 7H


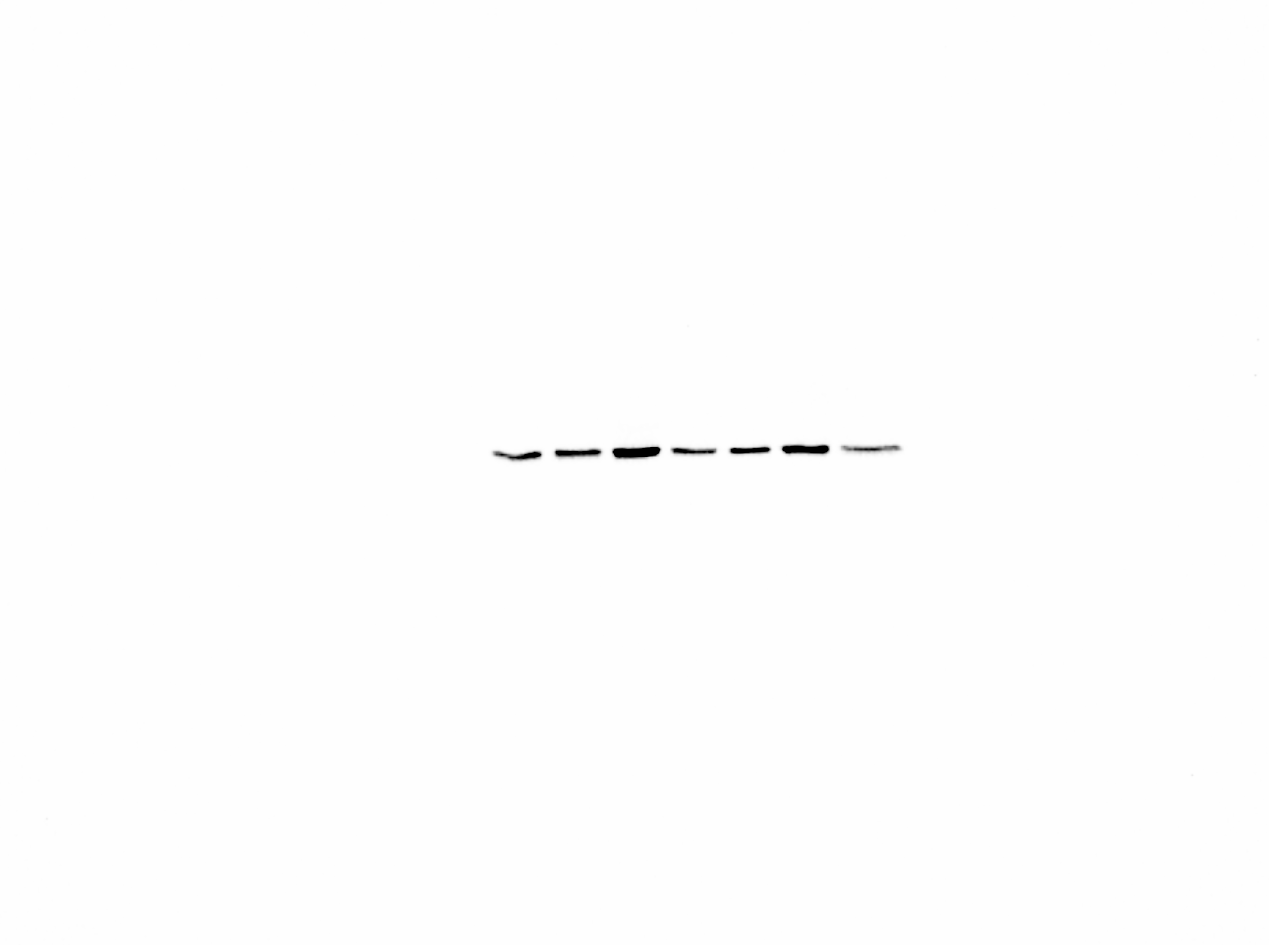


**(n)** Bax of Fig. 7H


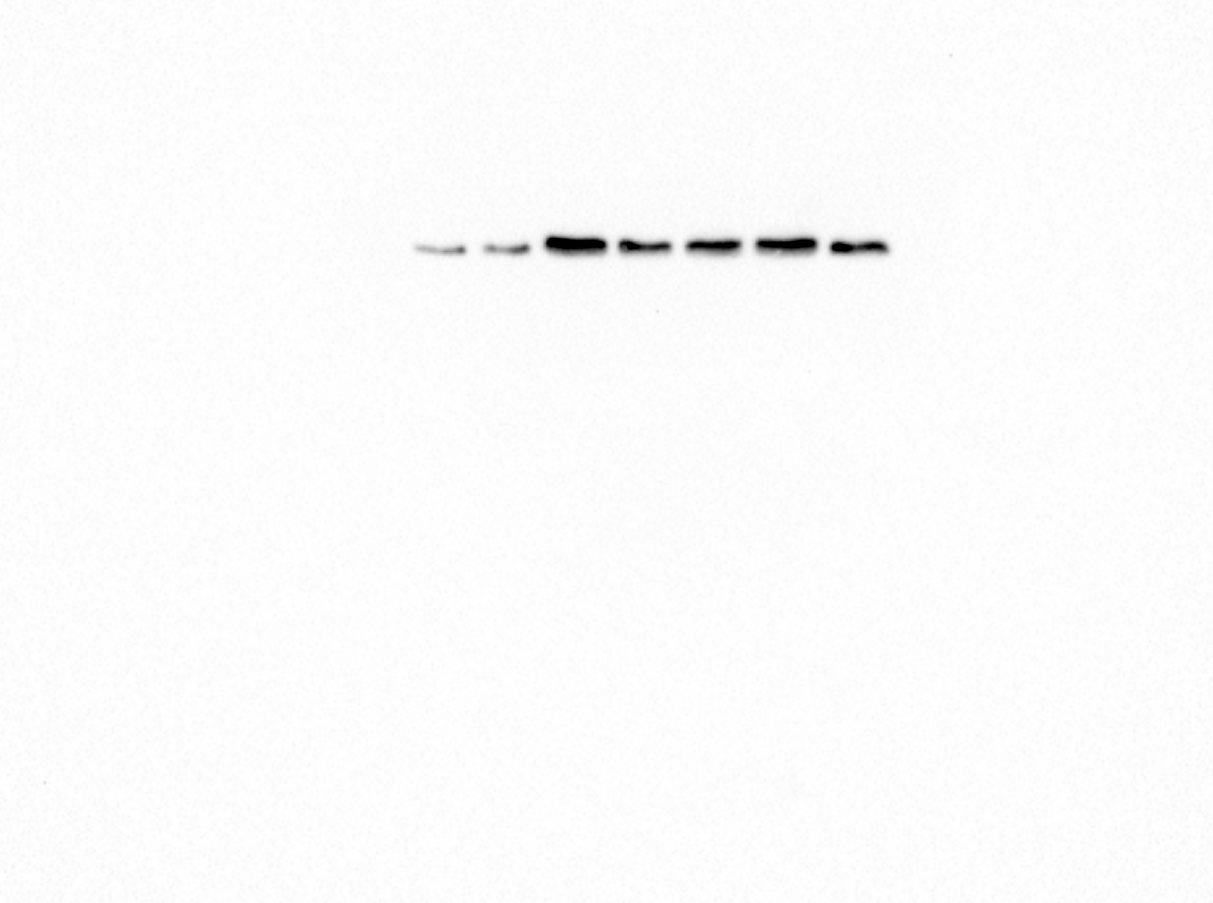


**(o)** GAPDH of Fig. 7H


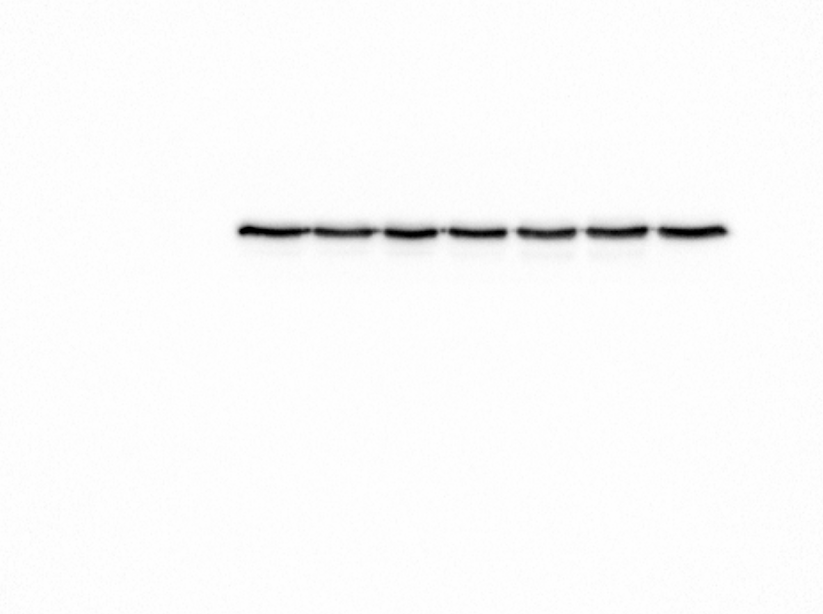

Supplement: Supplementary file 6 — Additional file 6. Original blot images of Fig. 6a, c and h. (a and b) Original blot images of Cap and GAPDH in the Fig. 6a, respectively. (C and g) Original blot images of Cap, GRP78, cleaved caspase-3, Bcl-2 and GAPDH in the Fig. 6c, respectively. (h-o) Original blot images of p-PERK, t-PERK, p-eIF2α, t-eIF2α, ATF4, CHOP, Bax and GAPDH in the Fig. 6c, respectively. [file 12866_2020_1986_MOESM6_ESM.docx]
